# Supplementary material for: Physical functioning in the lumbar spinal surgery population: A systematic review and narrative synthesis of outcome measures and measurement properties of the physical measures
Source: PLoS One. 2024 Aug 29;19(8):e0307004. doi: 10.1371/journal.pone.0307004 (PMC11361614; doi:10.1371/journal.pone.0307004)
Supplement: S1 Appendix — (DOCX) [file pone.0307004.s001.docx]

**S1 Appendix:** Search strategies

| **Contents** | **Page number** |
| --- | --- |
| **S1 Appendix:** Stage one and two search strategies | Stage one: 2  Stage two: 10 |

**Stage one**

**Databases used:** Medline (Ovid), Embase (Ovid), CINAHL (EBSCOhost), Scopus, Web of Science Core Collection, Health and Psychosocial Instruments (Ovid), PEDro

**Results summary:**

MEDLINE 9333

Embase  11175

CINAHL 4333

Scopus 9330

Web of Science 4906

HAPI 97

PEDro 270

Total after deduplication=18644

ProQuest Dissertations and Theses: 82

**Search notes:** Lines 6-11 and 21-29 (Medline) are a modified version of searches from the [NICE guideline](https://www.nice.org.uk/guidance/ng59/evidence/appendices-ag-pdf-2726157999) on low back pain and sciatica. Animal studies were removed from the search in Medline and Embase. No date or language limits were applied.

**MEDLINE (Ovid) run December 15, 2021 – N=9333**

1 lumbar vertebrae/ 56950

2 sciatica/ 5113

3 (lumbar vertebra* or lumbar spin* or lumbar dis#).tw,kf. 56838

4 (lumbar adj3 (stenosis or stenotic)).tw,kf. 4653

5 ((lumbar or lumbosacral or lumbo-sacral or low* back*) adj5 surg*).tw,kf. 11201

6 (lumbago or sciatica).tw,kf. 6273

7 (radiculopathy or radiculitis or radicular pain*).tw,kf. 9926

8 (nerve root* adj3 (pain* or avulsion or compress* or disorder* or pinch* or inflam* or imping* or irritat* or entrap* or trap*)).tw,kf. 2645

9 7 or 8 11927

10 (back$5 or lumbosacral or lumbo-sacral or lumbar).tw,kf. 443288

11 9 and 10 6480

12 or/1-6,11 98625

13 spinal fusion/ 28526

14 arthroplasty/ or arthroplasty, replacement/ or total disc replacement/ 16521

15 diskectomy/ or diskectomy, percutaneous/ 6405

16 laminectomy/ 10202

17 laminoplasty/ 588

18 foraminotomy/ 216

19 decompression, surgical/ 17037

20 su.fs. 2123323

21 ((disc or disk) adj3 (replac* or arthroplast*)).tw,kf. 2345

22 ((vertebra* or spine or spinal or lumbar) adj3 arthroplast*).tw,kf. 629

23 ((disc or discs or disk*) adj3 (remov* or excis* or surg* or resect* or replac*)).tw,kf. 6568

24 ((lumb* or lamina) adj3 (remov* or excis* or surg* or resect* or replac* or arthroplast*)).tw,kf. 9559

25 (spin* fusion or (lumbar adj2 fusion) or alif or plif or tlif or dlif or xlif or ollif or axlif or axialif or lif or arthrodesis or syndesis or spondylodesis or spondylosyndesis or artificial ankylosis or discectom* or diskectom* or laminectom* or laminotom* or laminoplast* or facetectom* or foraminotom* or fenestrat* or microdiscectom* or microdiskectom* or accutherm or disctrode or spinecath or transdiscal or apld or microdecompression or micro decompression or sequestrectom*).tw,kf. 67322

26 ((surg* or lumb* or disc or disk or intradiscal) adj3 decompress*).tw,kf. 11790

27 (micro* adj3 (surg* or endoscop* or laser)).tw,kf. 58142

28 ((disc or disk or intradiscal or intra discal or intervertebral or inter vertebral or percutaneous) adj3 (arthroplast* or biacuplast* or annuloplast* or electrothermal or thermomodulation)).tw,kf. 1155

29 (thermal adj3 procedure*).tw,kf. 683

30 or/13-29 2213964

31 physical functional performance/ 2100

32 cardiorespiratory fitness/ 2521

33 physical endurance/ 20087

34 "task performance and analysis"/ 32392

35 recovery of function/ 57367

36 disability evaluation/ 50411

37 work capacity evaluation/ 6190

38 lifting/ 2746

39 locomotion/ 27465

40 walking/ 37259

41 gait/ 31180

42 dependent ambulation/ 207

43 walking speed/ 2166

44 stair climbing/ 234

45 postural balance/ 26126

46 activities of daily living/ 69055

47 self care/ 34920

48 movement/ 77643

49 motor activity/ 99068

50 motor skills/ 25670

51 psychomotor performance/ 67280

52 range of motion, articular/ 56319

53 muscle strength/ 23548

54 health status/ 86880

55 functional status/ 851

56 exercise test/ 66422

57 walk test/ 2191

58 accelerometry/ 6728

59 ((daily or domestic or house or home) adj5 (activit* or task* or skill* or chore*)).tw,kf. 76882

60 (physical function* or functional activit* or independence or functional limitation* or activity limitation* or impairment* or performance* or endurance or acceleromet* or muscle strength or motor strength or mobility or postural balance or walk*).tw,kf. 1839881

61 (outcome* adj3 (clinical or physical or function*)).tw,kf. 313654

62 or/31-61 2651836

63 12 and 30 and 62 9664

64 63 not (exp animals/ not humans/) 9333

**Embase (Ovid) run December 15, 2021 – N=11175**

1 lumbar spine/ 54003

2 exp lumbar vertebra/ 24959

3 sciatica/ 2568

4 lumbar spinal stenosis/ 3072

5 (lumbar vertebra* or lumbar spin* or lumbar dis#).tw,kf. 83647

6 (lumbar adj3 (stenosis or stenotic)).tw,kf. 6210

7 ((lumbar or lumbosacral or lumbo-sacral or low* back*) adj5 surg*).tw,kf. 15057

8 (lumbago or sciatica).tw,kf. 8734

9 (radiculopathy or radiculitis or radicular pain*).tw,kf. 14795

10 (nerve root* adj3 (pain* or avulsion or compress* or disorder* or pinch* or inflam* or imping* or irritat* or entrap* or trap*)).tw,kf. 3819

11 9 or 10 17705

12 (back$5 or lumbosacral or lumbo-sacral or lumbar).tw,kf. 615882

13 11 and 12 9569

14 or/1-8,13 128279

15 exp spine fusion/ 35702

16 arthroplasty/ or replacement arthroplasty/ 23955

17 Spine surgery/ 28249

18 total disc replacement/ 1046

19 discectomy/ or percutaneous discectomy/ 5480

20 laminectomy/ 24969

21 laminoplasty/ 2898

22 foraminotomy/ 1009

23 spinal cord decompression/ 7153

24 su.fs. 2308976

25 ((disc or disk) adj3 (replac* or arthroplast*)).tw,kf. 3402

26 ((vertebra* or spine or spinal or lumbar) adj3 arthroplast*).tw,kf. 875

27 ((disc or discs or disk*) adj3 (remov* or excis* or surg* or resect* or replac*)).tw,kf. 8795

28 ((lumb* or lamina) adj3 (remov* or excis* or surg* or resect* or replac* or arthroplast*)).tw,kf. 12896

29 (spin* fusion or (lumbar adj2 fusion) or alif or plif or tlif or dlif or xlif or ollif or axlif or axialif or lif or arthrodesis or syndesis or spondylodesis or spondylosyndesis or artificial ankylosis or discectom* or diskectom* or laminectom* or laminotom* or laminoplast* or facetectom* or foraminotom* or fenestrat* or microdiscectom* or microdiskectom* or accutherm or disctrode or spinecath or transdiscal or apld or microdecompression or micro decompression or sequestrectom*).tw,kf. 90623

30 ((surg* or lumb* or disc or disk or intradiscal) adj3 decompress*).tw,kf. 16004

31 (micro* adj3 (surg* or endoscop* or laser)).tw,kf. 72488

32 ((disc or disk or intradiscal or intra discal or intervertebral or inter vertebral or percutaneous) adj3 (arthroplast* or biacuplast* or annuloplast* or electrothermal or thermomodulation)).tw,kf. 1670

33 (thermal adj3 procedure*).tw,kf. 901

34 or/15-33 2469731

35 physical performance/ 23889

36 cardiorespiratory fitness/ 6694

37 endurance/ 26328

38 task performance/ 150349

39 convalescence/ 57881

40 exp disability/ 183129

41 physical capacity/ or work capacity/ 27960

42 locomotion/ 79544

43 walking/ 77664

44 gait/ 61970

45 walking difficulty/ 13963

46 walking speed/ 19166

47 stair climbing/ 1590

48 body equilibrium/ 21510

49 daily life activity/ 100807

50 self care/ 66457

51 body movement/ or Limb movement/ or leg movement/ or Patient mobility/ or Physical mobility/ or spine mobility/ 36762

52 motor activity/ 51547

53 motor performance/ 85288

54 psychomotor performance/ 24624

55 "range of motion"/ 51694

56 muscle strength/ 72593

57 health status/ 138889

58 functional status/ 60752

59 exercise test/ 63712

60 walk test/ 4534

61 accelerometry/ 8615

62 ((daily or domestic or house or home) adj5 (activit* or task* or skill* or chore*)).tw,kf. 112178

63 (physical function* or functional activit* or independence or functional limitation* or activity limitation* or impairment* or performance* or endurance or acceleromet* or muscle strength or motor strength or mobility or postural balance or walk*).tw,kf. 2366216

64 (outcome* adj3 (clinical or physical or function*)).tw,kf. 488942

65 or/35-64 3620962

66 14 and 34 and 65 11467

67 66 not ((exp animal/ or nonhuman/) not exp human/) 11175

**CINAHL (EBSCOhost) run December 15, 2021 – N=4333**

| **#** | **Query** | **Limiters/Expanders** | **Results** |
| --- | --- | --- | --- |
| S60 | S12 AND S29 AND S59 | Search modes - Boolean/Phrase | 4,333 |
| S59 | S30 OR S31 OR S32 OR S33 OR S34 OR S35 OR S36 OR S37 OR S38 OR S39 OR S40 OR S41 OR S42 OR S43 OR S44 OR S45 OR S46 OR S47 OR S48 OR S49 OR S50 OR S51 OR S52 OR S53 OR S54 OR S55 OR S56 OR S57 OR S58 | Search modes - Boolean/Phrase | 820,326 |
| S58 | (outcome* N3 (clinical OR physical OR function*)) | Search modes - Boolean/Phrase | 116,278 |
| S57 | (physical function* OR functional activit* OR independence OR functional limitation* OR activity limitation* OR impairment* OR performance* OR endurance OR acceleromet* OR muscle strength OR motor strength OR mobility OR postural balance OR walk*) | Search modes - Boolean/Phrase | 522,395 |
| S56 | ((daily OR domestic OR house OR home) N5 (activit* OR task* OR skill* OR chore*)) | Search modes - Boolean/Phrase | 60,372 |
| S55 | (MH "Accelerometry") | Search modes - Boolean/Phrase | 4,991 |
| S54 | (MH "Exercise Test") | Search modes - Boolean/Phrase | 16,489 |
| S53 | (MH "Functional Status") | Search modes - Boolean/Phrase | 26,165 |
| S52 | (MH "Health Status") | Search modes - Boolean/Phrase | 58,561 |
| S51 | (MH "Muscle Strength") | Search modes - Boolean/Phrase | 20,638 |
| S50 | (MH "Range of Motion") | Search modes - Boolean/Phrase | 30,368 |
| S49 | (MH "Psychomotor Performance") | Search modes - Boolean/Phrase | 13,113 |
| S48 | (MH "Motor Skills") | Search modes - Boolean/Phrase | 11,652 |
| S47 | (MH "Motor Activity") | Search modes - Boolean/Phrase | 12,164 |
| S46 | (MH "Movement") | Search modes - Boolean/Phrase | 15,921 |
| S45 | (MH "Self Care") | Search modes - Boolean/Phrase | 42,653 |
| S44 | (MH "Activities of Daily Living") | Search modes - Boolean/Phrase | 35,249 |
| S43 | (MH "Balance, Postural") | Search modes - Boolean/Phrase | 17,952 |
| S42 | (MH "Stair Climbing") | Search modes - Boolean/Phrase | 899 |
| S41 | (MH "Walking Speed") | Search modes - Boolean/Phrase | 1,515 |
| S40 | (MH "Gait") | Search modes - Boolean/Phrase | 11,209 |
| S39 | (MH "Walking") | Search modes - Boolean/Phrase | 23,981 |
| S38 | (MH "Locomotion") | Search modes - Boolean/Phrase | 2,083 |
| S37 | (MH "Lifting") | Search modes - Boolean/Phrase | 2,842 |
| S36 | (MH "Work Capacity Evaluation") | Search modes - Boolean/Phrase | 1,704 |
| S35 | (MH "Disability Evaluation") | Search modes - Boolean/Phrase | 16,424 |
| S34 | (MH "Recovery") | Search modes - Boolean/Phrase | 35,915 |
| S33 | (MH "Task Performance and Analysis") | Search modes - Boolean/Phrase | 16,984 |
| S32 | (MH "Physical Endurance") | Search modes - Boolean/Phrase | 6,727 |
| S31 | (MH "Cardiorespiratory Fitness") | Search modes - Boolean/Phrase | 1,251 |
| S30 | (MH "Physical Performance") | Search modes - Boolean/Phrase | 6,052 |
| S29 | S13 OR S14 OR S15 OR S16 OR S17 OR S18 OR S19 OR S20 OR S21 OR S22 OR S23 OR S24 OR S25 OR S26 OR S27 OR S28 | Search modes - Boolean/Phrase | 416,971 |
| S28 | (thermal N3 procedure*) | Search modes - Boolean/Phrase | 121 |
| S27 | ((disc OR disk OR intradiscal OR intra discal OR intervertebral OR inter vertebral OR percutaneous) N3 (arthroplast* OR biacuplast* OR annuloplast* OR electrothermal OR thermomodulation)) | Search modes - Boolean/Phrase | 578 |
| S26 | (micro* N3 (surg* OR endoscop* OR laser)) | Search modes - Boolean/Phrase | 6,721 |
| S25 | ((surg* OR lumb* OR disc OR disk OR intradiscal) N3 decompress*) | Search modes - Boolean/Phrase | 7,148 |
| S24 | (spin* fusion OR (lumbar N2 fusion) OR alif OR plif OR tlif OR dlif OR xlif OR ollif OR axlif OR axialif OR lif OR arthrodesis OR syndesis OR spondylodesis OR spondylosyndesis OR artificial ankylosis OR discectom* OR diskectom* OR laminectom* OR laminotom* OR laminoplast* OR facetectom* OR foraminotom* OR fenestrat* OR microdiscectom* OR microdiskectom* OR accutherm OR disctrode OR spinecath OR transdiscal OR apld OR microdecompression OR micro decompression OR sequestrectom*) | Search modes - Boolean/Phrase | 24,205 |
| S23 | ((lumb* OR lamina) N3 (remov* OR excis* OR surg* OR resect* OR replac* OR arthroplast*)) | Search modes - Boolean/Phrase | 9,335 |
| S22 | ((disc OR discs OR disk*) N3 (remov* OR excis* OR surg* OR resect* OR replac*)) | Search modes - Boolean/Phrase | 4,212 |
| S21 | ((vertebra* OR spine OR spinal OR lumbar) N3 arthroplast*) | Search modes - Boolean/Phrase | 322 |
| S20 | ((disc OR disk) N3 (replac* OR arthroplast*)) | Search modes - Boolean/Phrase | 1,131 |
| S19 | MW "SU" | Search modes - Boolean/Phrase | 400,266 |
| S18 | (MH "Decompression, Surgical") | Search modes - Boolean/Phrase | 5,488 |
| S17 | (MH "Laminoplasty") | Search modes - Boolean/Phrase | 286 |
| S16 | (MH "Laminectomy") | Search modes - Boolean/Phrase | 2,209 |
| S15 | (MH "Diskectomy") | Search modes - Boolean/Phrase | 2,697 |
| S14 | (MH "Arthroplasty") OR (MH "Arthroplasty, Replacement") | Search modes - Boolean/Phrase | 7,725 |
| S13 | (MH "Spinal Fusion") | Search modes - Boolean/Phrase | 10,925 |
| S12 | S1 OR S2 OR S3 OR S4 OR S5 OR S6 OR S11 | Search modes - Boolean/Phrase | 33,296 |
| S11 | S9 AND S10 | Search modes - Boolean/Phrase | 3,454 |
| S10 | (back* OR lumbosacral OR lumbo-sacral OR lumbar) | Search modes - Boolean/Phrase | 929,608 |
| S9 | S7 OR S8 | Search modes - Boolean/Phrase | 5,104 |
| S8 | (nerve root* N3 (pain* OR avulsion OR compress* OR disorder* OR pinch* OR inflam* OR imping* OR irritat* OR entrap* OR trap*) | Search modes - Boolean/Phrase | 872 |
| S7 | (radiculopathy OR radiculitis OR radicular pain*) | Search modes - Boolean/Phrase | 4,564 |
| S6 | (lumbago OR sciatica) | Search modes - Boolean/Phrase | 2,479 |
| S5 | ((lumbar OR lumbosacral OR lumbo-sacral OR low* back*) N5 surg*) | Search modes - Boolean/Phrase | 10,273 |
| S4 | (lumbar N3 (stenosis OR stenotic)) | Search modes - Boolean/Phrase | 1,992 |
| S3 | (lumbar vertebra* OR lumbar spin* OR lumbar disk OR lumbar disc) | Search modes - Boolean/Phrase | 28,392 |
| S2 | (MH "Sciatica") | Search modes - Boolean/Phrase | 1,803 |
| S1 | (MH "Lumbar Vertebrae") | Search modes - Boolean/Phrase | 19,160 |

**Scopus run December 15, 2021 – N=9330**

(TITLE-ABS-KEY("lumbar vertebra*" OR "lumbar spin*" OR "lumbar disk" OR "lumbar disc") OR TITLE-ABS-KEY(lumbar W/3 (stenosis OR stenotic)) OR TITLE-ABS-KEY((lumbar OR lumbosacral OR "lumbo-sacral" OR "low* back*") W/5 surg*) OR TITLE-ABS-KEY(lumbago OR sciatica) OR (TITLE-ABS-KEY(radiculopathy OR radiculitis OR "radicular pain*") OR TITLE-ABS-KEY("nerve root*" W/3 (pain* OR avulsion OR compress* OR disorder* OR pinch* OR inflam* OR imping* OR irritat* OR entrap* OR trap*)) AND TITLE-ABS-KEY(back* OR lumbosacral OR "lumbo-sacral" OR lumbar))) AND (TITLE-ABS-KEY((disc OR disk) W/3 (replac* OR arthroplast*)) OR TITLE-ABS-KEY((vertebra* OR spine OR spinal OR lumbar) W/3 arthroplast*) OR TITLE-ABS-KEY((disc OR discs OR disk*) W/3 (remov* OR excis* OR surg* OR resect* OR replac*)) OR TITLE-ABS-KEY((lumb* OR lamina) W/3 (remov* OR excis* OR surg* OR resect* OR replac* OR arthroplast*)) OR TITLE-ABS-KEY(("spin* fusion" OR (lumbar W/2 fusion) OR alif OR plif OR tlif OR dlif OR xlif OR ollif OR axlif OR axialif OR lif OR arthrodesis OR syndesis OR spondylodesis OR spondylosyndesis OR "artificial ankylosis" OR discectom* OR diskectom* OR laminectom* OR laminotom* OR laminoplast* OR facetectom* OR foraminotom* OR fenestrat* OR microdiscectom* OR microdiskectom* OR accutherm OR disctrode OR spinecath OR transdiscal OR apld OR microdecompression OR "micro decompression" OR sequestrectom*)) OR TITLE-ABS-KEY((surg* OR lumb* OR disc OR disk OR intradiscal) W/3 decompress*) OR TITLE-ABS-KEY(micro* W/3 (surg* OR endoscop* OR laser)) OR TITLE-ABS-KEY((disc OR disk OR intradiscal OR "intra discal" OR intervertebral OR "inter vertebral" OR percutaneous) W/3 (arthroplast* OR biacuplast* OR annuloplast* OR electrothermal OR thermomodulation)) OR TITLE-ABS-KEY(thermal W/3 procedure*)) AND (TITLE-ABS-KEY((daily OR domestic OR house OR home) W/5 (activit* OR task* OR skill* OR chore*)) OR TITLE-ABS-KEY("physical function*" OR "functional activit*" OR independence OR "functional limitation*" OR "activity limitation*" OR impairment* OR performance* OR endurance OR acceleromet* OR "muscle strength" OR "motor strength" OR mobility OR "postural balance" OR walk*) OR TITLE-ABS-KEY(outcome* W/3 (clinical OR physical OR function*)))

**Web of Science run December 15, 2021 – N=4906**

(TS=("lumbar vertebra*" OR "lumbar spin*" OR "lumbar disk" OR "lumbar disc") OR TS=(lumbar NEAR/3 (stenosis OR stenotic)) OR TS=((lumbar OR lumbosacral OR "lumbo-sacral" OR "low* back*") NEAR/5 surg*) OR TS=(lumbago OR sciatica) OR (TS=(radiculopathy OR radiculitis OR "radicular pain*") OR TS=("nerve root*" NEAR/3 (pain* OR avulsion OR compress* OR disorder* OR pinch* OR inflam* OR imping* OR irritat* OR entrap* OR trap*))) AND TS=(back* OR lumbosacral OR "lumbo-sacral" OR lumbar)) AND (TS=((disc OR disk) NEAR/3 (replac* OR arthroplast*)) OR TS=((vertebra* OR spine OR spinal OR lumbar) NEAR/3 arthroplast*) OR TS=((disc OR discs OR disk*) NEAR/3 (remov* OR excis* OR surg* OR resect* OR replac*)) OR TS=((lumb* OR lamina) NEAR/3 (remov* OR excis* OR surg* OR resect* OR replac* OR arthroplast*)) OR TS=(("spin* fusion" OR (lumbar NEAR/2 fusion) OR alif OR plif OR tlif OR dlif OR xlif OR ollif OR axlif OR axialif OR lif OR arthrodesis OR syndesis OR spondylodesis OR spondylosyndesis OR "artificial ankylosis" OR discectom* OR diskectom* OR laminectom* OR laminotom* OR laminoplast* OR facetectom* OR foraminotom* OR fenestrat* OR microdiscectom* OR microdiskectom* OR accutherm OR disctrode OR spinecath OR transdiscal OR apld OR microdecompression OR "micro decompression" OR sequestrectom*)) OR TS=((surg* OR lumb* OR disc OR disk OR intradiscal) NEAR/3 decompress*) OR TS=(micro* NEAR/3 (surg* OR endoscop* OR laser)) OR TS=((disc OR disk OR intradiscal OR "intra discal" OR intervertebral OR "inter vertebral" OR percutaneous) NEAR/3 (arthroplast* OR biacuplast* OR annuloplast* OR electrothermal OR thermomodulation)) OR TS=(thermal NEAR/3 procedure*)) AND (TS=((daily OR domestic OR house OR home) NEAR/5 (activit* OR task* OR skill* OR chore*)) OR TS=("physical function*" OR "functional activit*" OR independence OR "functional limitation*" OR "activity limitation*" OR impairment* OR performance* OR endurance OR acceleromet* OR "muscle strength" OR "motor strength" OR mobility OR "postural balance" OR walk*) OR TS=(outcome* NEAR/3 (clinical OR physical OR function*)))

**Health and Psychosocial Instruments (Ovid) run December 15, 2021 – N=97**

1 (lumbar or spine or spinal or disc or disk or lumbosacral or lumbo-sacral or back* or lumbago or sciatica or stenosis or stenotic).mp,hw. 5071

2 surg*.mp,hw. 1971

3 (fusion or arthrodesis or syndesis or spondylodesis or spondylosyndesis or artificial ankylosis or discectom* or diskectom* or laminectom* or laminotom* or laminoplast* or facetectom* or foraminotom* or fenestrat* or microdiscectom* or microdiskectom* or accutherm or disctrode or spinecath or transdiscal or decompress* or microdecompression or micro decompression or sequestrectom* or arthroplast*).mp,hw. 181

4 2 or 3 2115

5 1 and 4 97

**PEDro run December 15, 2021 – N=270**

Abstract & Title: surg* lumbar

Body part: lumbar spine, sacroiliac joint or pelvis

Match all search terms (AND)

=202

Abstract & Title: Surg* spin*

Body part: lumbar spine, sacroiliac joint or pelvis

Match all search terms (AND)

=199

Deduplicated total=270

**Stage two**

**Databases used:** Medline (Ovid), Embase (Ovid), CINAHL (EBSCOhost), Scopus, Web of Science Core Collection, Health and Psychosocial Instruments (Ovid)

| **Results summary for May 27, 2022 search** | **Results summary for July 10, 2023 search** |
| --- | --- |
| MEDLINE 2435 | MEDLINE 215 |
| Embase  1254 | Embase  197 |
| CINAHL 933 | CINAHL 109 |
| Scopus 2202 | Scopus 198 |
| Web of Science 1485 | Web of Science 154 |
| HAPI 10 | HAPI 9 |
| Total after deduplication=4368 | Total after deduplication=407 |
|  |  |
| ProQuest D&T 53 | ProQuest D&T 2 |

**Search notes:** Lines 6-11 and 21-29 (Medline) are a modified version of searches from the [NICE guideline](https://www.nice.org.uk/guidance/ng59/evidence/appendices-ag-pdf-2726157999) on low back pain and sciatica. Animal studies were removed from the search in Medline and Embase. No date or language limits were applied.

The validated COSMIN highly sensitive search filter for finding studies on measurement properties for PubMed^1^ was translated for the selected databases and applied to the search:

Medline(Ovid) – Validated PubMed version translated to Medline (Ovid) by Maren

Embase (Ovid) – Used Embase.com Filter 1 (translation by E.P. Jansma, Medical Library, VU University, Amsterdam, The Netherlands) translated to Embase (Ovid) by Maren

CINAHL, Scopus, Web of Science, HAPI – Translated from Medline (Ovid) version by Maren

**MEDLINE (Ovid) run May 27, 2022 – N=2435**

1 lumbar vertebrae/ 58311

2 sciatica/ 5144

3 (lumbar vertebra* or lumbar spin* or lumbar dis#).tw,kf. 58262

4 (lumbar adj3 (stenosis or stenotic)).tw,kf. 4834

5 ((lumbar or lumbosacral or lumbo-sacral or low* back*) adj5 surg*).tw,kf. 11634

6 (lumbago or sciatica).tw,kf. 6378

7 (radiculopathy or radiculitis or radicular pain*).tw,kf. 10234

8 (nerve root* adj3 (pain* or avulsion or compress* or disorder* or pinch* or inflam* or imping* or irritat* or entrap* or trap*)).tw,kf. 2696

9 7 or 8 12266

10 (back$5 or lumbosacral or lumbo-sacral or lumbar).tw,kf. 456032

11 9 and 10 6676

12 or/1-6,11 100977

13 spinal fusion/ 29525

14 arthroplasty/ or arthroplasty, replacement/ or total disc replacement/ 16798

15 diskectomy/ or diskectomy, percutaneous/ 6608

16 laminectomy/ 10318

17 laminoplasty/ 642

18 foraminotomy/ 227

19 decompression, surgical/ 17371

20 su.fs. 2169862

21 ((disc or disk) adj3 (replac* or arthroplast*)).tw,kf. 2420

22 ((vertebra* or spine or spinal or lumbar) adj3 arthroplast*).tw,kf. 644

23 ((disc or discs or disk*) adj3 (remov* or excis* or surg* or resect* or replac*)).tw,kf. 6689

24 ((lumb* or lamina) adj3 (remov* or excis* or surg* or resect* or replac* or arthroplast*)).tw,kf. 9907

25 (spin* fusion or (lumbar adj2 fusion) or alif or plif or tlif or dlif or xlif or ollif or axlif or axialif or lif or arthrodesis or syndesis or spondylodesis or spondylosyndesis or artificial ankylosis or discectom* or diskectom* or laminectom* or laminotom* or laminoplast* or facetectom* or foraminotom* or fenestrat* or microdiscectom* or microdiskectom* or accutherm or disctrode or spinecath or transdiscal or apld or microdecompression or micro decompression or sequestrectom*).tw,kf. 69337

26 ((surg* or lumb* or disc or disk or intradiscal) adj3 decompress*).tw,kf. 12187

27 (micro* adj3 (surg* or endoscop* or laser)).tw,kf. 59644

28 ((disc or disk or intradiscal or intra discal or intervertebral or inter vertebral or percutaneous) adj3 (arthroplast* or biacuplast* or annuloplast* or electrothermal or thermomodulation)).tw,kf. 1188

29 (thermal adj3 procedure*).tw,kf. 707

30 or/13-29 2262726

31 exp "range of motion, articular"/ 58111

32 (range of motion or ROM).tw,kf. 45500

33 Schober.tw,kf. 337

34 (finger* adj3 (floor or toe or knee or fibular head)).tw,kf. 1183

35 (wrist crease adj3 floor).tw,kf. 2

36 (straight leg raise* or SLR or Lasegue*).tw,kf. 2680

37 (isometric strength or isokinetic strength).tw,kf. 3324

38 (motor control or movement control or functional movement screen* or FMS or sitting one leg knee extension or posterior pelvic tilt or waiter's bow or one leg stance).tw,kf. 25085

39 gait/ or gait analysis/ 33014

40 walking speed/ 2360

41 (spatiotemporal gait or spatio temporal gait or stride length or stride duration or gait speed or cadence or gait asymmetry or stance phase or swing phase or double limb support or single limb support).tw,kf. 17931

42 (inclinometer or goniometer or kyphometer or electromagnetic tracking).tw,kf. 4128

43 muscle strength dynamometer/ 1945

44 (dynamometer or manual muscle test or MedX or Cybex or Kin-Com or RehaGait or JAMAR).tw,kf. 8663

45 or/31-44 161581

46 (aerobic capacity or VO2* or bicycle ergomet* or maximal graded exercise* or steep ramp).tw,kf. 36033

47 (Biering Sorensen or modified Sorensen).tw,kf. 136

48 Roman chair.tw,kf. 32

49 (sternum adj3 (ground or floor)).tw,kf. 6

50 muscle endurance.tw,kf. 1679

51 ((back or body or trunk) adj3 endurance).tw,kf. 1182

52 prone bridge.tw,kf. 44

53 ((lower extremities or (hips and knees)) adj5 (90deg* or "90 degree*")).tw,kf. 32

54 (arch-up* or sit-up* or squat* or dumbbell press*).tw,kf. 8907

55 (double limb stance or single limb stance or stork stand* or flamingo balance or y-balance).tw,kf. 840

56 CTSIB.tw,kf. 61

57 (clinical test* adj2 sensory interaction adj2 balance).tw,kf. 156

58 (clinical test* adj2 sensory integration adj2 balance).tw,kf. 54

59 (Berg balance scale or Tinetti* or performance oriented mobility assessment* or tandem walk*).tw,kf. 3453

60 lower extremity motor coordination test*.tw,kf. 14

61 (chair adj3 (stand* or rise*)).tw,kf. 2754

62 (sit to stand or stand up or stand ups or roll*).tw,kf. 47143

63 (lie adj2 sit).tw,kf. 53

64 (bed adj2 chair).tw,kf. 344

65 step*.tw,kf. 760530

66 ((stand or standing) adj2 continuous).tw,kf. 71

67 functional capacity evaluation.tw,kf. 258

68 lifting/ 2801

69 (lift* or progressive isoinertial lifting evaluation or pile).tw,kf. 32256

70 (forward reach or functional reach).tw,kf. 1185

71 walk test/ 2386

72 (self-paced walk* or 4-meter walk* or 4-metre walk* or 5-meter walk* or 5-metre walk* or 10-meter walk* or 10-metre walk* or 15-meter walk* or 15-metre walk* or 50-meter walk* or 50-metre walk* or 50-foot walk* or 5-minute walk* or 6-minute walk* or treadmill or overground walk*).tw,kf. 42392

73 (walk adj3 hall*).tw,kf. 82

74 shuttle walk*.tw,kf. 760

75 (stair* adj2 climb*).tw,kf. 3242

76 ("timed up and go" or TUG or "8 foot up and go").tw,kf. 7405

77 (Physical capability assessment tool or PCAT or aggregated functional performance test or aggregated assessment of physical function or short physical performance battery or cumulated ambulation score or functional independence measure or Katz ADL index).tw,kf. 5869

78 (Activity measure for post-acute care 6 clicks or Activity measure for postacute care 6 clicks).tw,kf. 16

79 (Physiotherapy functional mobility profile or Barthel index).tw,kf. 6860

80 or/46-79 935713

81 lying.tw,kf. 27274

82 (time adj5 stand*).tw,kf. 19918

83 (constant postures or active postures or sedentary postures or sedentary activity or walking time or walking distance or claudication index or walking speed or daily walking events or light intensity or moderate intensity or vigorous intensity or activity count or gait cycles or gait posture index or physical activity).tw,kf. 165836

84 accelerometry/ 7037

85 wearable electronic devices/ or fitness trackers/ 7076

86 (acceleromet* or activity monitor or pedomet* or GPS or watch or smartwatch).tw,kf. 63469

87 (6WT adj3 app*).tw,kf. 10

88 or/81-87 265812

89 45 or 80 or 88 1299593

90 12 and 30 and 89 3226

91 instrumentation.fs. 688301

92 methods.fs. 4145501

93 validation study.pt. 109049

94 comparative study.pt. 1911105

95 psychometrics/ 84730

96 psychometr*.ti,ab,kf. 57751

97 (clinimetr* or clinometr*).mp. 1426

98 exp outcome assessment, health care/ 1286294

99 outcome assessment.ti,ab,kf. 7567

100 outcome measure*.mp. 268056

101 observer variation/ 44620

102 observer variation.ti,ab,kf. 1430

103 exp health status indicators/ 337260

104 exp "reproducibility of results"/ 448136

105 reproducib*.ti,ab,kf. 181383

106 discriminant analysis/ 11698

107 (reliab* or unreliab* or valid* or coefficient or homogeneity or homogeneous or internal consistency).ti,ab,kf. 1663742

108 (cronbach* and (alpha or alphas)).ti,ab,kf. 28289

109 (item and (correlation* or selection* or reduction*)).ti,ab,kf. 27245

110 (agreement or precision or imprecision or precise values).mp. 483335

111 (test and retest).ti,ab,kf. 31856

112 (stability or interrater or inter-rater or intrarater or intra-rater or intertester or inter-tester or intratester or intra-tester or interobserver or inter-observer or intraobserver or intra-observer or intertechnician or inter-technician or intratechnician or intra-technician or interexaminer or inter-examiner or intraexaminer or intra-examiner or interassay or inter-assay or intraassay or intra-assay or interindividual or inter-individual or intraindividual or intra-individual or interparticipant or inter-participant or intraparticipant or intra-participant or kappa or kappa's or kappas).ti,ab,kf. 702814

113 repeatab*.mp. 41214

114 ((replicab* or repeated) and (measure or measures or findings or result or results or test or tests)).mp. 227127

115 (generaliza* or generalisa* or concordance).ti,ab,kf. 109080

116 (intraclass and correlation).ti,ab,kf. 29534

117 (discriminative or known group or factor analysis or factor analyses or factor structure or factor structures or dimension* or subscale*).ti,ab,kf. 774240

118 (multitrait and scaling and (analysis or analyses)).ti,ab,kf. 149

119 (item discriminant or interscale correlation* or error or errors or individual variability or interval variability or rate variability).ti,ab,kf. 384943

120 (variability and (analysis or values)).ti,ab,kf. 116618

121 (uncertainty and (measurement or measuring)).ti,ab,kf. 9585

122 (sensitiv* or responsive*).ti,ab,kf. 1776996

123 (limit and detection).ti,ab,kf. 107840

124 (minimal detectable concentration or interpretab*).ti,ab,kf. 13513

125 ((minimal or minimally or clinical or clinically) and (important or significant or detectable) and (change or difference)).ti,ab,kf. 282051

126 (small* and (real or detectable) and (change or difference)).ti,ab,kf. 8768

127 (meaningful change or ceiling effect or floor effect or item response model or IRT or Rasch or differential item functioning or DIF or computer adaptive testing or item bank or cross-cultural equivalence).ti,ab,kf. 16465

128 or/91-127 10757949

129 90 and 128 2582

130 129 not (exp animals/ not humans/) 2435

For July 10, 2023 search, the following lines were added to the Medline search:
131 ("20220527" or "20220528" or "20220529" or 2022053* or 202206* or 202207* or 202208* or 202209* or 20221* or 2023*).dt,ez,da.

132 130 and 131

**Embase (Ovid) run May 27, 2022 – N=1254**

1 lumbar spine/ 55381

2 exp lumbar vertebra/ 25915

3 sciatica/ 2693

4 lumbar spinal stenosis/ 3274

5 (lumbar vertebra* or lumbar spin* or lumbar dis#).tw,kf. 85659

6 (lumbar adj3 (stenosis or stenotic)).tw,kf. 6458

7 ((lumbar or lumbosacral or lumbo-sacral or low* back*) adj5 surg*).tw,kf. 15601

8 (lumbago or sciatica).tw,kf. 8866

9 (radiculopathy or radiculitis or radicular pain*).tw,kf. 15205

10 (nerve root* adj3 (pain* or avulsion or compress* or disorder* or pinch* or inflam* or imping* or irritat* or entrap* or trap*)).tw,kf. 3898

11 9 or 10 18165

12 (back$5 or lumbosacral or lumbo-sacral or lumbar).tw,kf. 633593

13 11 and 12 9828

14 or/1-8,13 131711

15 exp spine fusion/ 36972

16 arthroplasty/ or replacement arthroplasty/ 24494

17 Spine surgery/ 29330

18 total disc replacement/ 1074

19 discectomy/ or percutaneous discectomy/ 5783

20 laminectomy/ 25688

21 laminoplasty/ 2992

22 foraminotomy/ 1056

23 spinal cord decompression/ 7374

24 su.fs. 2360674

25 ((disc or disk) adj3 (replac* or arthroplast*)).tw,kf. 3489

26 ((vertebra* or spine or spinal or lumbar) adj3 arthroplast*).tw,kf. 896

27 ((disc or discs or disk*) adj3 (remov* or excis* or surg* or resect* or replac*)).tw,kf. 8947

28 ((lumb* or lamina) adj3 (remov* or excis* or surg* or resect* or replac* or arthroplast*)).tw,kf. 13330

29 (spin* fusion or (lumbar adj2 fusion) or alif or plif or tlif or dlif or xlif or ollif or axlif or axialif or lif or arthrodesis or syndesis or spondylodesis or spondylosyndesis or artificial ankylosis or discectom* or diskectom* or laminectom* or laminotom* or laminoplast* or facetectom* or foraminotom* or fenestrat* or microdiscectom* or microdiskectom* or accutherm or disctrode or spinecath or transdiscal or apld or microdecompression or micro decompression or sequestrectom*).tw,kf. 93149

30 ((surg* or lumb* or disc or disk or intradiscal) adj3 decompress*).tw,kf. 16525

31 (micro* adj3 (surg* or endoscop* or laser)).tw,kf. 74335

32 ((disc or disk or intradiscal or intra discal or intervertebral or inter vertebral or percutaneous) adj3 (arthroplast* or biacuplast* or annuloplast* or electrothermal or thermomodulation)).tw,kf. 1717

33 (thermal adj3 procedure*).tw,kf. 933

34 or/15-33 2525718

35 "range of motion"/ 54399

36 (range of motion or ROM).tw,kf. 57826

37 (finger* adj3 (floor or toe or knee or fibular head)).tw,kf. 1811

38 (wrist crease adj3 floor).tw,kf. 2

39 (Schober or straight leg raise* or SLR or Lasegue* or isometric strength or isokinetic strength or motor control or movement control or functional movement screen* or FMS or sitting one leg knee extension or posterior pelvic tilt or waiter's bow or one leg stance).tw,kf. 43264

40 gait/ or gait analysis system/ 64633

41 (spatiotemporal gait or spatio temporal gait or stride length or stride duration).tw,kf. 6017

42 exp walking parameters/ 25874

43 goniometer/ or exp goniometry/ 5641

44 (gait speed or cadence or gait asymmetry or stance phase or swing phase or double limb support or single limb support or inclinometer or goniometer or kyphometer or electromagnetic tracking).tw,kf. 26820

45 exp dynamometer/ 9396

46 (dynamometer or manual muscle test or MedX or Cybex or Kin-Com or RehaGait or JAMAR).tw,kf. 13104

47 or/35-46 223714

48 bicycle ergometry/ or bicycle ergometer/ 14256

49 maximal oxygen uptake/ 1293

50 (aerobic capacity or VO2* or bicycle ergomet* or maximal graded exercise* or steep ramp or muscle endurance or Biering Sorensen or modified Sorensen or Roman chair).tw,kf. 54375

51 (sternum adj3 (ground or floor)).tw,kf. 11

52 ((back or body or trunk) adj3 endurance).tw,kf. 1435

53 prone bridge.tw,kf. 57

54 ((lower extremities or (hips and knees)) adj5 (90deg* or "90 degree*")).tw,kf. 55

55 "squatting (exercise)"/ 179

56 (arch-up* or sit-up* or squat* or dumbbell press* or double limb stance or single limb stance or stork stand* or flamingo balance or y-balance).tw,kf. 11425

57 CTSIB.tw,kf. 89

58 (clinical test* adj2 sensory interaction adj2 balance).tw,kf. 236

59 (clinical test* adj2 sensory integration adj2 balance).tw,kf. 72

60 berg balance scale/ 3262

61 performance oriented mobility assessment/ 598

62 (Berg balance scale or Tinetti* or performance oriented mobility assessment* or tandem walk* or lower extremity motor coordination test*).tw,kf. 5456

63 (chair adj3 (stand* or rise*)).tw,kf. 4222

64 (sit to stand or stand up or stand ups or roll*).tw,kf. 60310

65 (lie adj2 sit).tw,kf. 86

66 (bed adj2 chair).tw,kf. 620

67 step*.tw,kf. 963801

68 ((stand or standing) adj2 continuous).tw,kf. 90

69 functional capacity evaluation.tw,kf. 331

70 (lift* or progressive isoinertial lifting evaluation or pile or forward reach or functional reach).tw,kf. 43386

71 walk test/ or exp shuttle walk test/ or six minute walk test/ 19332

72 (self-paced walk* or 4-meter walk* or 4-metre walk* or 5-meter walk* or 5-metre walk* or 10-meter walk* or 10-metre walk* or 15-meter walk* or 15-metre walk* or 50-meter walk* or 50-metre walk* or 50-foot walk* or 5-minute walk* or 6-minute walk* or treadmill or overground walk*).tw,kf. 61982

73 (walk adj3 hall*).tw,kf. 168

74 shuttle walk*.tw,kf. 1514

75 (stair* adj2 climb*).tw,kf. 5132

76 "timed up and go test"/ 5321

77 Functional Independence Measure/ 4797

78 katz index/ 787

79 ("timed up and go" or TUG or "8 foot up and go" or Physical capability assessment tool or PCAT or aggregated functional performance test or aggregated assessment of physical function or short physical performance battery or cumulated ambulation score or functional independence measure or Katz ADL index).tw,kf. 19592

80 Activity measure for post-acute care 6 clicks.tw,kf. 19

81 Activity measure for postacute care 6 clicks.tw,kf. 1

82 Barthel index/ 9957

83 (Physiotherapy functional mobility profile or Barthel index).tw,kf. 10641

84 or/48-83 1223028

85 recumbency/ 9693

86 lying.tw,kf. 32884

87 (time adj5 stand*).tw,kf. 30008

88 low intensity exercise/ or moderate intensity exercise/ or high intensity exercise/ 1158

89 accelerometer/ or accelerometry/ 22372

90 (constant postures or active postures or sedentary postures or sedentary activity or walking time or walking distance or claudication index or walking speed or daily walking events or light intensity or moderate intensity or vigorous intensity or activity count or gait cycles or gait posture index or physical activity or acceleromet*).tw,kf. 235324

91 wearable computer/ or activity tracker/ or pedometer/ or smart watch/ 5473

92 (activity monitor or pedomet* or GPS or watch or smartwatch).tw,kf. 62111

93 (6WT adj3 app*).tw,kf. 10

94 or/85-93 359883

95 47 or 84 or 94 1702183

96 14 and 34 and 95 3677

97 exp intermethod comparison/ 283427

98 exp data collection method/ 1268179

99 exp validation study/ 96357

100 exp feasibility study/ 164635

101 exp pilot study/ 185715

102 exp psychometry/ 107201

103 exp reproducibility/ 245447

104 reproducib*.ti,ab. 235632

105 audit.ti,ab. 83849

106 psychometr*.ti,ab. 67175

107 (clinimetr* or clinometr*).ti,ab. 1920

108 exp observer variation/ 20714

109 observer variation.ti,ab. 1781

110 exp discriminant analysis/ 23802

111 exp validity/ 120968

112 (reliab* or valid* or coefficient or internal consistency).ti,ab. 2041729

113 (cronbach* and (alpha or alphas)).ti,ab. 33926

114 (item correlation or item correlations or item selection or item selections or item reduction or item reductions).ti,ab. 3001

115 (agreement or precision or imprecision or precise values).ti,ab. 571809

116 test-retest.ti,ab. 36535

117 (test and retest).ti,ab. 38630

118 (reliab* and (test or retest)).ti,ab. 143439

119 (stability or interrater or inter-rater or intrarater or intra-rater or intertester or inter-tester or intratester or intra-tester or interobserver or inter-observer or intraobserver or intra-observer or intertechnician or inter-technician or intratechnician or intra-technician or interexaminer or inter-examiner or intraexaminer or intra-examiner or interassay or inter-assay or intraassay or intra-assay or interindividual or inter-individual or intraindividual or intra-individual or interparticipant or inter-participant or intraparticipant or intra-participant or kappa or kappa's or kappas).ti,ab. 818031

120 coefficient of variation.ti,ab. 33394

121 repeatab*.ti,ab. 50325

122 ((replicab* or repeated) and (measure or measures or findings or result or results or test or tests)).ti,ab. 339563

123 (generaliza* or generalisa* or concordance).ti,ab. 148411

124 (intraclass and correlation).ti,ab. 35407

125 (discriminative or known group or factor analysis or factor analyses or factor structure or factor structures or dimensionality or subscale*).ti,ab. 177481

126 (multitrait scaling analysis or multitrait scaling analyses).ti,ab. 110

127 (item discriminant or interscale correlation or interscale correlations).ti,ab. 313

128 ((error or errors) and (measure* or correlat* or evaluat* or accuracy or accurate or precision or mean)).ti,ab. 285106

129 (individual variability or interval variability or rate variability).ti,ab. 44777

130 (individual variability or interval variability or rate variability or variability analysis).ti,ab. 45544

131 (uncertainty and (measurement or measuring)).ti,ab. 10599

132 standard error of measurement.ti,ab. 2808

133 (sensitiv* or responsive*).ti,ab. 2281475

134 (limit and detection).ti,ab. 125540

135 (minimal detectable concentration or interpretab*).ti,ab. 16476

136 (small* and (real or detectable) and (change or difference)).ti,ab. 14249

137 (meaningful change or minimal important change or minimal important difference or minimally important change or minimally important difference or minimal detectable change or minimal detectable difference or minimally detectable change or minimally detectable difference or minimal real change or minimal real difference or minimally real change or minimally real difference or ceiling effect or floor effect or item response model or IRT or Rasch or differential item functioning or DIF or computer adaptive testing or item bank or cross-cultural equivalence).ti,ab. 26438

138 or/97-137 7333682

139 96 and 138 1336

140 139 not ((exp animal/ or nonhuman/) not exp human/) 1254

For July 10, 2023 search, the following line was added to the Embase search:

141 limit 140 to dc=20220527-20230710

**CINAHL (EBSCOhost) run May 27, 2022 – N=933**

| **#** | **Query** | **Limiters/Expanders** | **Results** |
| --- | --- | --- | --- |
| S125 | S87 AND S124 | Search modes - Boolean/Phrase | 933 |
| S124 | S88 OR S89 OR S90 OR S91 OR S92 OR S93 OR S94 OR S95 OR S96 OR S97 OR S98 OR S99 OR S100 OR S101 OR S102 OR S103 OR S104 OR S105 OR S106 OR S107 OR S108 OR S109 OR S110 OR S111 OR S112 OR S113 OR S114 OR S115 OR S116 OR S117 OR S118 OR S119 OR S120 OR S121 OR S122 OR S123 | Search modes - Boolean/Phrase | 1,534,850 |
| S123 | TI (meaningful change OR ceiling effect OR floor effect OR item response model OR IRT OR Rasch OR differential item functioning OR DIF OR computer adaptive testing OR item bank OR cross-cultural equivalence) OR AB (meaningful change OR ceiling effect OR floor effect OR item response model OR IRT OR Rasch OR differential item functioning OR DIF OR computer adaptive testing OR item bank OR cross-cultural equivalence) | Search modes - Boolean/Phrase | 10,364 |
| S122 | TI (small* AND (real OR detectable) AND (change OR difference)) OR AB (small* AND (real OR detectable) AND (change OR difference)) | Search modes - Boolean/Phrase | 2,812 |
| S121 | TI ((minimal OR minimally OR clinical OR clinically) AND (important OR significant OR detectable) AND (change OR difference)) OR AB ((minimal OR minimally OR clinical OR clinically) AND (important OR significant OR detectable) AND (change OR difference)) | Search modes - Boolean/Phrase | 136,341 |
| S120 | TI (minimal detectable concentration OR interpretab*) OR AB (minimal detectable concentration OR interpretab*) | Search modes - Boolean/Phrase | 2,516 |
| S119 | TI (limit AND detection) OR AB (limit AND detection) | Search modes - Boolean/Phrase | 3,722 |
| S118 | TI (sensitiv* OR responsive*) OR AB (sensitiv* OR responsive*) | Search modes - Boolean/Phrase | 215,486 |
| S117 | TI (uncertainty AND (measurement OR measuring)) OR AB (uncertainty AND (measurement OR measuring)) | Search modes - Boolean/Phrase | 1,365 |
| S116 | TI (variability AND (analysis OR values)) OR AB (variability AND (analysis OR values)) | Search modes - Boolean/Phrase | 22,841 |
| S115 | TI (item discriminant OR interscale correlation* OR error OR errors OR individual variability OR interval variability OR rate variability) OR AB (item discriminant OR interscale correlation* OR error OR errors OR individual variability OR interval variability OR rate variability) | Search modes - Boolean/Phrase | 80,396 |
| S114 | TI (multitrait AND scaling AND (analysis OR analyses)) OR AB (multitrait AND scaling AND (analysis OR analyses)) | Search modes - Boolean/Phrase | 61 |
| S113 | TI (discriminative OR known group OR factor analysis OR factor analyses OR factor structure OR factor structures OR dimension* OR subscale*) OR AB (discriminative OR known group OR factor analysis OR factor analyses OR factor structure OR factor structures OR dimension* OR subscale*) | Search modes - Boolean/Phrase | 171,938 |
| S112 | TI (intraclass AND correlation) OR AB (intraclass AND correlation) | Search modes - Boolean/Phrase | 13,435 |
| S111 | TI (generaliza* OR generalisa* OR concordance) OR AB (generaliza* OR generalisa* OR concordance) | Search modes - Boolean/Phrase | 27,910 |
| S110 | ((replicab* OR repeated) AND (measure OR measures OR findings OR result OR results OR test OR tests)) | Search modes - Boolean/Phrase | 96,690 |
| S109 | repeatab* | Search modes - Boolean/Phrase | 4,617 |
| S108 | TI (stability OR interrater OR inter-rater OR intrarater OR intra-rater OR intertester OR inter-tester OR intratester OR intra-tester OR interobserver OR inter-observer OR intraobserver OR intra-observer OR intertechnician OR inter-technician OR intratechnician OR intra-technician OR interexaminer OR inter-examiner OR intraexaminer OR intra-examiner OR interassay OR inter-assay OR intraassay OR intra-assay OR interindividual OR inter-individual OR intraindividual OR intra-individual OR interparticipant OR inter-participant OR intraparticipant OR intra-participant OR kappa OR kappa's OR kappas) OR AB (stability OR interrater OR inter-rater OR intrarater OR intra-rater OR intertester OR inter-tester OR intratester OR intra-tester OR interobserver OR inter-observer OR intraobserver OR intra-observer OR intertechnician OR inter-technician OR intratechnician OR intra-technician OR interexaminer OR inter-examiner OR intraexaminer OR intra-examiner OR interassay OR inter-assay OR intraassay OR intra-assay OR interindividual OR inter-individual OR intraindividual OR intra-individual OR interparticipant OR inter-participant OR intraparticipant OR intra-participant OR kappa OR kappa's OR kappas) | Search modes - Boolean/Phrase | 83,369 |
| S107 | TI (test AND retest) OR AB (test AND retest) | Search modes - Boolean/Phrase | 15,050 |
| S106 | (agreement OR precision OR imprecision OR precise values) | Search modes - Boolean/Phrase | 74,802 |
| S105 | TI (item and (correlation* or selection* or reduction*)) OR AB (item and (correlation* or selection* or reduction*)) | Search modes - Boolean/Phrase | 17,790 |
| S104 | TI (cronbach* and (alpha or alphas)) OR AB (cronbach* and (alpha or alphas)) | Search modes - Boolean/Phrase | 12,247 |
| S103 | (MH "Measurement Error+") | Search modes - Boolean/Phrase | 1,093 |
| S102 | (MH "Reliability and Validity+") | Search modes - Boolean/Phrase | 271,010 |
| S101 | TI (reliab* OR unreliab* OR valid* OR coefficient OR homogeneity OR homogeneous OR internal consistency) OR AB (reliab* OR unreliab* OR valid* OR coefficient OR homogeneity OR homogeneous OR internal consistency) | Search modes - Boolean/Phrase | 345,216 |
| S100 | (MH "Discriminant Analysis") | Search modes - Boolean/Phrase | 3,352 |
| S99 | TI reproducib* OR AB reproducib* | Search modes - Boolean/Phrase | 19,578 |
| S98 | (MH "Reproducibility of Results") | Search modes - Boolean/Phrase | 70,164 |
| S97 | (MH "Health Status Indicators") | Search modes - Boolean/Phrase | 12,027 |
| S96 | TI observer variation OR AB observer variation | Search modes - Boolean/Phrase | 351 |
| S95 | outcome measure* | Search modes - Boolean/Phrase | 142,560 |
| S94 | TI outcome assessment OR AB outcome assessment | Search modes - Boolean/Phrase | 14,725 |
| S93 | (MH "Outcome Assessment") | Search modes - Boolean/Phrase | 48,319 |
| S92 | (clinimetr* OR clinometr*) | Search modes - Boolean/Phrase | 710 |
| S91 | TI psychometr* OR AB psychometr* | Search modes - Boolean/Phrase | 28,325 |
| S90 | (MH "Psychometrics") | Search modes - Boolean/Phrase | 30,942 |
| S89 | (MH "Comparative Studies") | Search modes - Boolean/Phrase | 437,125 |
| S88 | (MH "Validation Studies") | Search modes - Boolean/Phrase | 345,754 |
| S87 | S12 AND S29 AND S86 | Search modes - Boolean/Phrase | 1,539 |
| S86 | S42 OR S76 OR S85 | Search modes - Boolean/Phrase | 368,216 |
| S85 | S77 OR S78 OR S79 OR S80 OR S81 OR S82 OR S83 OR S84 | Search modes - Boolean/Phrase | 137,415 |
| S84 | TI (6WT N3 app*) OR AB (6WT N3 app*) | Search modes - Boolean/Phrase | 3 |
| S83 | TI (activity monitor OR pedomet* OR GPS OR watch OR smartwatch) OR AB (activity monitor OR pedomet* OR GPS OR watch OR smartwatch) | Search modes - Boolean/Phrase | 28,459 |
| S82 | (MH "Fitness Trackers") OR (MH "Pedometers") | Search modes - Boolean/Phrase | 1,739 |
| S81 | TI (constant postures OR active postures OR sedentary postures OR sedentary activity OR walking time OR walking distance OR claudication index OR walking speed OR daily walking events OR light intensity OR moderate intensity OR vigorous intensity OR activity count OR gait cycles OR gait posture index OR physical activity OR acceleromet*) OR AB (constant postures OR active postures OR sedentary postures OR sedentary activity OR walking time OR walking distance OR claudication index OR walking speed OR daily walking events OR light intensity OR moderate intensity OR vigorous intensity OR activity count OR gait cycles OR gait posture index OR physical activity OR acceleromet*) | Search modes - Boolean/Phrase | 91,631 |
| S80 | (MH "Accelerometers") OR (MH "Accelerometry") | Search modes - Boolean/Phrase | 7,590 |
| S79 | (MH "Exercise Intensity") | Search modes - Boolean/Phrase | 11,443 |
| S78 | TI (time N5 stand*) OR AB (time N5 stand*) | Search modes - Boolean/Phrase | 7,634 |
| S77 | TI lying or AB lying | Search modes - Boolean/Phrase | 3,442 |
| S76 | S43 OR S44 OR S45 OR S46 OR S47 OR S48 OR S49 OR S50 OR S51 OR S52 OR S53 OR S54 OR S55 OR S56 OR S57 OR S58 OR S59 OR S60 OR S61 OR S62 OR S63 OR S64 OR S65 OR S66 OR S67 OR S68 OR S69 OR S70 OR S71 OR S72 OR S73 OR S74 OR S75 | Search modes - Boolean/Phrase | 186,535 |
| S75 | TI (Physiotherapy functional mobility profile OR Barthel index) OR AB (Physiotherapy functional mobility profile OR Barthel index) | Search modes - Boolean/Phrase | 3,737 |
| S74 | (MH "Barthel Index") | Search modes - Boolean/Phrase | 6,427 |
| S73 | TI Activity measure for postacute care 6 clicks OR AB Activity measure for postacute care 6 clicks | Search modes - Boolean/Phrase | 2 |
| S72 | TI Activity measure for post-acute care 6 clicks OR AB Activity measure for post-acute care 6 clicks | Search modes - Boolean/Phrase | 6 |
| S71 | TI ("timed up and go" OR TUG OR "8 foot up and go" OR Physical capability assessment tool OR PCAT OR aggregated functional performance test OR aggregated assessment of physical function OR short physical performance battery OR cumulated ambulation score OR functional independence measure OR Katz ADL index) OR AB ("timed up and go" OR TUG OR "8 foot up and go" OR Physical capability assessment tool OR PCAT OR aggregated functional performance test OR aggregated assessment of physical function OR short physical performance battery OR cumulated ambulation score OR functional independence measure OR Katz ADL index) | Search modes - Boolean/Phrase | 7,563 |
| S70 | TI (stair* N2 climb*) OR AB (stair* N2 climb*) | Search modes - Boolean/Phrase | 1,617 |
| S69 | (MH "Stair Climbing") | Search modes - Boolean/Phrase | 909 |
| S68 | TI shuttle walk* OR AB shuttle walk* | Search modes - Boolean/Phrase | 345 |
| S67 | TI (walk N3 hall*) OR AB (walk N3 hall*) | Search modes - Boolean/Phrase | 38 |
| S66 | TI (self-paced walk* OR 4-meter walk* OR 4-metre walk* OR 5-meter walk* OR 5-metre walk* OR 10-meter walk* OR 10-metre walk* OR 15-meter walk* OR 15-metre walk* OR 50-meter walk* OR 50-metre walk* OR 50-foot walk* OR 5-minute walk* OR 6-minute walk* OR treadmill OR overground walk*) OR AB (self-paced walk* OR 4-meter walk* OR 4-metre walk* OR 5-meter walk* OR 5-metre walk* OR 10-meter walk* OR 10-metre walk* OR 15-meter walk* OR 15-metre walk* OR 50-meter walk* OR 50-metre walk* OR 50-foot walk* OR 5-minute walk* OR 6-minute walk* OR treadmill OR overground walk*) | Search modes - Boolean/Phrase | 13,801 |
| S65 | TI (lift* OR progressive isoinertial lifting evaluation OR pile OR forward reach OR functional reach) OR AB (lift* OR progressive isoinertial lifting evaluation OR pile OR forward reach OR functional reach) | Search modes - Boolean/Phrase | 10,562 |
| S64 | (MH "Reaching") | Search modes - Boolean/Phrase | 678 |
| S63 | (MH "Lifting") | Search modes - Boolean/Phrase | 2,853 |
| S62 | TI functional capacity evaluation OR AB functional capacity evaluation | Search modes - Boolean/Phrase | 402 |
| S61 | TI ((stand OR standing) N2 continuous) OR AB (stand OR standing) N2 continuous) | Search modes - Boolean/Phrase | 28 |
| S60 | TI step* OR AB step* | Search modes - Boolean/Phrase | 118,512 |
| S59 | (MH "Step") | Search modes - Boolean/Phrase | 1,177 |
| S58 | TI (bed N2 chair) OR AB (bed N2 chair) | Search modes - Boolean/Phrase | 248 |
| S57 | TI (lie N2 sit) OR AB (lie N2 sit) | Search modes - Boolean/Phrase | 26 |
| S56 | TI (sit to stand OR stand up OR stand ups OR roll*) OR AB (sit to stand OR stand up OR stand ups OR roll*) | Search modes - Boolean/Phrase | 14,195 |
| S55 | TI (chair N3 (stand* OR rise*)) OR AB (chair N3 (stand* OR rise*)) | Search modes - Boolean/Phrase | 1,633 |
| S54 | TI (Berg balance scale OR Tinetti* OR performance oriented mobility assessment* OR tandem walk* OR lower extremity motor coordination test*) OR AB (Berg balance scale OR Tinetti* OR performance oriented mobility assessment* OR tandem walk* OR lower extremity motor coordination test*) | Search modes - Boolean/Phrase | 2,403 |
| S53 | TI (clinical test* N2 sensory integration N2 balance) OR AB (clinical test* N2 sensory integration N2 balance) | Search modes - Boolean/Phrase | 39 |
| S52 | TI (clinical test* N2 sensory interaction N2 balance) OR AB (clinical test* N2 sensory interaction N2 balance) | Search modes - Boolean/Phrase | 106 |
| S51 | TI CTSIB OR AB CTSIB | Search modes - Boolean/Phrase | 49 |
| S50 | TI (arch-up* OR sit-up* OR squat* OR dumbbell press* OR double limb stance OR single limb stance OR stork stand* OR flamingo balance OR y-balance) OR AB (arch-up* OR sit-up* OR squat* OR dumbbell press* OR double limb stance OR single limb stance OR stork stand* OR flamingo balance OR y-balance) | Search modes - Boolean/Phrase | 4,654 |
| S49 | (MH "One Leg Stand") | Search modes - Boolean/Phrase | 219 |
| S48 | TI ((lower extremities OR (hips AND knees)) N5 (90deg* OR "90 degree*")) OR AB ((lower extremities OR (hips AND knees)) N5 (90deg* OR "90 degree*")) | Search modes - Boolean/Phrase | 54 |
| S47 | TI prone bridge OR AB prone bridge | Search modes - Boolean/Phrase | 41 |
| S46 | TI ((back OR body OR trunk) N3 endurance) OR AB ((back OR body OR trunk) N3 endurance) | Search modes - Boolean/Phrase | 823 |
| S45 | TI (sternum N3 (ground OR floor)) OR AB (sternum N3 (ground OR floor)) | Search modes - Boolean/Phrase | 4 |
| S44 | TI (aerobic capacity OR VO2* OR bicycle ergomet* OR maximal graded exercise* OR steep ramp OR muscle endurance OR Biering Sorensen OR modified Sorensen OR Roman chair) OR AB (aerobic capacity OR VO2* OR bicycle ergomet* OR maximal graded exercise* OR steep ramp OR muscle endurance OR Biering Sorensen OR modified Sorensen OR Roman chair) | Search modes - Boolean/Phrase | 11,460 |
| S43 | (MH "Aerobic Capacity") | Search modes - Boolean/Phrase | 2,436 |
| S42 | S30 OR S31 OR S32 OR S33 OR S34 OR S35 OR S36 OR S37 OR S38 OR S39 OR S40 OR S41 | Search modes - Boolean/Phrase | 82,059 |
| S41 | TI (dynamometer OR manual muscle test OR MedX OR Cybex OR Kin-Com OR RehaGait OR JAMAR) OR AB (dynamometer OR manual muscle test OR MedX OR Cybex OR Kin-Com OR RehaGait OR JAMAR) | Search modes - Boolean/Phrase | 4,298 |
| S40 | (MH "Dynamometry") | Search modes - Boolean/Phrase | 7,764 |
| S39 | TI (gait speed OR cadence OR gait asymmetry OR stance phase OR swing phase OR double limb support OR single limb support OR inclinometer OR goniometer OR kyphometer OR electromagnetic tracking) OR AB (gait speed OR cadence OR gait asymmetry OR stance phase OR swing phase OR double limb support OR single limb support OR inclinometer OR goniometer OR kyphometer OR electromagnetic tracking) | Search modes - Boolean/Phrase | 10,489 |
| S38 | (MH "Goniometry") | Search modes - Boolean/Phrase | 3,241 |
| S37 | (MH "Walking Speed") | Search modes - Boolean/Phrase | 1,644 |
| S36 | TI (spatiotemporal gait OR spatio temporal gait OR stride length OR stride duration) OR AB (spatiotemporal gait OR spatio temporal gait OR stride length OR stride duration) | Search modes - Boolean/Phrase | 2,388 |
| S35 | (MH "Gait") OR (MH "Gait Analysis") | Search modes - Boolean/Phrase | 16,852 |
| S34 | TI (Schober OR straight leg raise* OR SLR OR Lasegue* OR isometric strength OR isokinetic strength OR motor control OR movement control OR functional movement screen* OR FMS OR sitting one leg knee extension OR posterior pelvic tilt OR waiter's bow OR one leg stance) OR AB (Schober OR straight leg raise* OR SLR OR Lasegue* OR isometric strength OR isokinetic strength OR motor control OR movement control OR functional movement screen* OR FMS OR sitting one leg knee extension OR posterior pelvic tilt OR waiter's bow OR one leg stance) | Search modes - Boolean/Phrase | 15,544 |
| S33 | TI (wrist crease N3 floor) OR AB (wrist crease N3 floor) | Search modes - Boolean/Phrase | 3 |
| S32 | TI (finger* N3 (floor OR toe OR knee OR fibular head)) OR AB (finger* N3 (floor OR toe OR knee OR fibular head)) | Search modes - Boolean/Phrase | 607 |
| S31 | TI (range of motion OR ROM) OR AB (range of motion OR ROM) | Search modes - Boolean/Phrase | 22,563 |
| S30 | (MH "Range of Motion") | Search modes - Boolean/Phrase | 30,999 |
| S29 | S13 OR S14 OR S15 OR S16 OR S17 OR S18 OR S19 OR S20 OR S21 OR S22 OR S23 OR S24 OR S25 OR S26 OR S27 OR S28 | Search modes - Boolean/Phrase | 430,214 |
| S28 | (thermal N3 procedure*) | Search modes - Boolean/Phrase | 123 |
| S27 | ((disc OR disk OR intradiscal OR intra discal OR intervertebral OR inter vertebral OR percutaneous) N3 (arthroplast* OR biacuplast* OR annuloplast* OR electrothermal OR thermomodulation)) | Search modes - Boolean/Phrase | 584 |
| S26 | (micro* N3 (surg* OR endoscop* OR laser)) | Search modes - Boolean/Phrase | 6,905 |
| S25 | ((surg* OR lumb* OR disc OR disk OR intradiscal) N3 decompress*) | Search modes - Boolean/Phrase | 7,332 |
| S24 | (spin* fusion OR (lumbar N2 fusion) OR alif OR plif OR tlif OR dlif OR xlif OR ollif OR axlif OR axialif OR lif OR arthrodesis OR syndesis OR spondylodesis OR spondylosyndesis OR artificial ankylosis OR discectom* OR diskectom* OR laminectom* OR laminotom* OR laminoplast* OR facetectom* OR foraminotom* OR fenestrat* OR microdiscectom* OR microdiskectom* OR accutherm OR disctrode OR spinecath OR transdiscal OR apld OR microdecompression OR micro decompression OR sequestrectom*) | Search modes - Boolean/Phrase | 24,940 |
| S23 | ((lumb* OR lamina) N3 (remov* OR excis* OR surg* OR resect* OR replac* OR arthroplast*)) | Search modes - Boolean/Phrase | 9,697 |
| S22 | ((disc OR discs OR disk*) N3 (remov* OR excis* OR surg* OR resect* OR replac*)) | Search modes - Boolean/Phrase | 4,312 |
| S21 | ((vertebra* OR spine OR spinal OR lumbar) N3 arthroplast*) | Search modes - Boolean/Phrase | 336 |
| S20 | ((disc OR disk) N3 (replac* OR arthroplast*)) | Search modes - Boolean/Phrase | 1,151 |
| S19 | MW "SU" | Search modes - Boolean/Phrase | 413,117 |
| S18 | (MH "Decompression, Surgical") | Search modes - Boolean/Phrase | 5,608 |
| S17 | (MH "Laminoplasty") | Search modes - Boolean/Phrase | 304 |
| S16 | (MH "Laminectomy") | Search modes - Boolean/Phrase | 2,253 |
| S15 | (MH "Diskectomy") | Search modes - Boolean/Phrase | 2,765 |
| S14 | (MH "Arthroplasty") OR (MH "Arthroplasty, Replacement") | Search modes - Boolean/Phrase | 7,865 |
| S13 | (MH "Spinal Fusion") | Search modes - Boolean/Phrase | 11,284 |
| S12 | S1 OR S2 OR S3 OR S4 OR S5 OR S6 OR S11 | Search modes - Boolean/Phrase | 34,178 |
| S11 | S9 AND S10 | Search modes - Boolean/Phrase | 3,532 |
| S10 | (back* OR lumbosacral OR lumbo-sacral OR lumbar) | Search modes - Boolean/Phrase | 967,459 |
| S9 | S7 OR S8 | Search modes - Boolean/Phrase | 5,210 |
| S8 | (nerve root* N3 (pain* OR avulsion OR compress* OR disorder* OR pinch* OR inflam* OR imping* OR irritat* OR entrap* OR trap*) | Search modes - Boolean/Phrase | 887 |
| S7 | (radiculopathy OR radiculitis OR radicular pain*) | Search modes - Boolean/Phrase | 4,660 |
| S6 | (lumbago OR sciatica) | Search modes - Boolean/Phrase | 2,519 |
| S5 | ((lumbar OR lumbosacral OR lumbo-sacral OR low* back*) N5 surg*) | Search modes - Boolean/Phrase | 10,647 |
| S4 | (lumbar N3 (stenosis OR stenotic)) | Search modes - Boolean/Phrase | 2,072 |
| S3 | (lumbar vertebra* OR lumbar spin* OR lumbar disk OR lumbar disc) | Search modes - Boolean/Phrase | 29,172 |
| S2 | (MH "Sciatica") | Search modes - Boolean/Phrase | 1,822 |
| S1 | (MH "Lumbar Vertebrae") | Search modes - Boolean/Phrase | 19,716 |

For July 10, 2023 search, the following line was added to the CINAHL search:

S126 EM 20220527- OR ZD "in process"

**Scopus run May 27, 2022 – N=2202**

((TITLE-ABS-KEY("lumbar vertebra*" OR "lumbar spin*" OR "lumbar disk" OR "lumbar disc") OR TITLE-ABS-KEY(lumbar W/3 (stenosis OR stenotic)) OR TITLE-ABS-KEY((lumbar OR lumbosacral OR "lumbo-sacral" OR "low* back*") W/5 surg*) OR TITLE-ABS-KEY(lumbago OR sciatica) OR (TITLE-ABS-KEY(radiculopathy OR radiculitis OR "radicular pain*") OR TITLE-ABS-KEY("nerve root*" W/3 (pain* OR avulsion OR compress* OR disorder* OR pinch* OR inflam* OR imping* OR irritat* OR entrap* OR trap*)) AND TITLE-ABS-KEY(back* OR lumbosacral OR "lumbo-sacral" OR lumbar))) AND (TITLE-ABS-KEY((disc OR disk) W/3 (replac* OR arthroplast*)) OR TITLE-ABS-KEY((vertebra* OR spine OR spinal OR lumbar) W/3 arthroplast*) OR TITLE-ABS-KEY((disc OR discs OR disk*) W/3 (remov* OR excis* OR surg* OR resect* OR replac*)) OR TITLE-ABS-KEY((lumb* OR lamina) W/3 (remov* OR excis* OR surg* OR resect* OR replac* OR arthroplast*)) OR TITLE-ABS-KEY(("spin* fusion" OR (lumbar W/2 fusion) OR alif OR plif OR tlif OR dlif OR xlif OR ollif OR axlif OR axialif OR lif OR arthrodesis OR syndesis OR spondylodesis OR spondylosyndesis OR "artificial ankylosis" OR discectom* OR diskectom* OR laminectom* OR laminotom* OR laminoplast* OR facetectom* OR foraminotom* OR fenestrat* OR microdiscectom* OR microdiskectom* OR accutherm OR disctrode OR spinecath OR transdiscal OR apld OR microdecompression OR "micro decompression" OR sequestrectom*)) OR TITLE-ABS-KEY((surg* OR lumb* OR disc OR disk OR intradiscal) W/3 decompress*) OR TITLE-ABS-KEY(micro* W/3 (surg* OR endoscop* OR laser)) OR TITLE-ABS-KEY((disc OR disk OR intradiscal OR "intra discal" OR intervertebral OR "inter vertebral" OR percutaneous) W/3 (arthroplast* OR biacuplast* OR annuloplast* OR electrothermal OR thermomodulation)) OR TITLE-ABS-KEY(thermal W/3 procedure*))) AND (TITLE-ABS-KEY("range of motion" OR ROM) OR TITLE-ABS-KEY(finger* W/3 (floor OR toe OR knee OR "fibular head")) OR TITLE-ABS-KEY(wrist crease W/3 floor) OR TITLE-ABS-KEY(Schober OR "straight leg raise*" OR SLR OR Lasegue* OR "isometric strength" OR "isokinetic strength" OR "motor control" OR "movement control" OR "functional movement screen*" OR FMS OR "sitting one leg knee extension" OR "posterior pelvic tilt" OR "waiter's bow" OR "one leg stance") OR TITLE-ABS-KEY("gait analysis" OR "spatiotemporal gait" OR "spatio temporal gait" OR "stride length" OR "stride duration" OR "walking speed" OR "gait speed" OR cadence OR "gait asymmetry" OR "stance phase" OR "swing phase" OR "double limb support" OR "single limb support" OR inclinometer OR goniometer OR kyphometer OR "electromagnetic tracking") OR TITLE-ABS-KEY(dynamometer OR "manual muscle test" OR MedX OR Cybex OR Kin-Com OR RehaGait OR JAMAR) OR TITLE-ABS-KEY("aerobic capacity" OR VO2* OR "bicycle ergomet*" OR "maximal graded exercise*" OR "steep ramp" OR "muscle endurance" OR "Biering Sorensen" OR "modified Sorensen" OR "Roman chair") OR TITLE-ABS-KEY(sternum W/3 (ground OR floor)) OR TITLE-ABS-KEY((back OR body OR trunk) W/3 endurance) OR TITLE-ABS-KEY("prone bridge") OR TITLE-ABS-KEY(("lower extremities" OR (hips AND knees)) W/5 (90deg* OR "90 degree*")) OR TITLE-ABS-KEY(arch-up* OR sit-up* OR squat* OR "dumbbell press*" OR "double limb stance" OR "single limb stance" OR "stork stand*" OR "flamingo balance" OR y-balance OR CTSIB) OR TITLE-ABS-KEY(("clinical test*" W/2 "sensory interaction" W/2 balance) OR ("clinical test*" W/2 "sensory integration" W/2 balance)) OR TITLE-ABS-KEY(("Berg balance scale" OR Tinetti* OR "performance oriented mobility assessment*" OR "tandem walk*" OR "lower extremity motor coordination test*")) OR TITLE-ABS-KEY(chair W/3 (stand* OR rise*)) OR TITLE-ABS-KEY("sit to stand" OR "stand up" OR "stand ups" OR roll*) OR TITLE-ABS-KEY(lie W/2 sit) OR TITLE-ABS-KEY(bed W/2 chair) OR TITLE-ABS-KEY(step*) OR TITLE-ABS-KEY((stand or standing) W/2 continuous) OR TITLE-ABS-KEY("functional capacity evaluation" OR lift* OR "progressive isoinertial lifting evaluation" OR pile OR "forward reach" OR "functional reach") OR TITLE-ABS-KEY("walk test*" OR "self-paced walk*" OR "4-meter walk*" OR "4-metre walk*" OR "5-meter walk*" OR "5-metre walk*" OR "10-meter walk*" OR "10-metre walk*" OR "15-meter walk*" OR "15-metre walk*" OR "50-meter walk*" OR "50-metre walk*" OR "50-foot walk*" OR "5-minute walk*" OR "6-minute walk*" OR treadmill OR "overground walk*" OR "shuttle walk") OR TITLE-ABS-KEY(walk W/3 hall*) OR TITLE-ABS-KEY(stair* W/2 climb*) OR TITLE-ABS-KEY("timed up and go" OR TUG OR "8 foot up and go" OR "Physical capability assessment tool" OR PCAT OR "aggregated functional performance test" OR "aggregated assessment of physical function" OR "short physical performance battery" OR "cumulated ambulation score" OR "functional independence measure" OR "Katz ADL index" OR "Katz index") OR TITLE-ABS-KEY("Activity measure for post-acute care 6 clicks" OR "Activity measure for postacute care 6 clicks" OR "Physiotherapy functional mobility profile" OR "Barthel index") OR (TITLE-ABS-KEY(lying) OR TITLE-ABS-KEY(time W/5 stand*) OR TITLE-ABS-KEY("constant postures" OR "active postures" OR "sedentary postures" OR "sedentary activity" OR "walking time" OR "walking distance" OR "claudication index" OR "daily walking events" OR "light intensity" OR "moderate intensity" OR "vigorous intensity" OR "activity count" OR "gait cycles" OR "gait posture index" OR "physical activity" OR acceleromet*) OR TITLE-ABS-KEY("wearable electronic devices" OR "fitness tracker*" OR "activity monitor*" OR pedomet* OR gps OR watch OR "smart watch") OR TITLE-ABS-KEY(6wt W/3 app*))) AND ((KEY(instrumentation OR methods) OR TITLE-ABS-KEY("validation study" OR "comparative study") OR TITLE-ABS-KEY(psychometr* OR clinimetr* OR clinometr*) OR TITLE-ABS-KEY("outcome assessment" OR "outcome measure*" OR "observer variation" OR "health status indicators") OR TITLE-ABS-KEY(reproducib* OR "discriminant analysis" ) OR TITLE-ABS (reliab* OR unreliab* OR valid* OR coefficient OR homogeneity OR homogeneous OR "internal consistency") OR TITLE-ABS((cronbach* AND (alpha OR alphas))) OR TITLE-ABS(item AND (correlation* OR selection* OR reduction*))) OR TITLE-ABS-KEY((agreement OR precision OR imprecision OR precise values)) OR TITLE-ABS(test AND retest) OR TITLE-ABS(stability OR interrater OR "inter-rater" OR intrarater OR "intra-rater" OR intertester OR "inter-tester" OR intratester OR "intra-tester" OR interobserver OR "inter-observer" OR intraobserver OR "intra-observer" OR intertechnician OR "inter-technician" OR intratechnician OR "intra-technician" OR interexaminer OR "inter-examiner" OR intraexaminer OR "intra-examiner" OR interassay OR "inter-assay" OR intraassay OR "intra-assay" OR interindividual OR "inter-individual" OR intraindividual OR "intra-individual" OR interparticipant OR "inter-participant" OR intraparticipant OR "intra-participant" OR kappa OR kappa's OR kappas) OR TITLE-ABS-KEY(repeatab*) OR TITLE-ABS((replicab* OR repeated) AND (measure OR measures OR findings OR result OR results OR test OR tests)) OR TITLE-ABS(generaliza* OR generalisa* OR concordance) OR TITLE-ABS(intraclass AND correlation) OR TITLE-ABS((discriminative OR "known group" OR "factor analysis" OR "factor analyses" OR "factor structure" OR "factor structures" OR dimension* OR subscale*)) OR TITLE-ABS((multitrait AND scaling AND (analysis OR analyses))) OR TITLE-ABS(("item discriminant" OR "interscale correlation*" OR error OR errors OR "individual variability" OR "interval variability" OR "rate variability")) OR TITLE-ABS((variability AND (analysis OR values))) OR TITLE-ABS((uncertainty AND (measurement OR measuring))) OR TITLE-ABS((sensitiv* OR responsive*)) OR TITLE-ABS((limit AND detection)) OR TITLE-ABS(("minimal detectable concentration" OR interpretab*)) OR TITLE-ABS(((minimal OR minimally OR clinical OR clinically) AND (important OR significant OR detectable) AND (change OR difference))) OR TITLE-ABS((small* AND (real OR detectable) AND (change OR difference))) OR TITLE-ABS(("meaningful change" OR "ceiling effect" OR "floor effect" OR "item response model" OR IRT OR Rasch OR "differential item functioning" OR DIF OR "computer adaptive testing" OR "item bank" OR "cross-cultural equivalence")))

For July 10, 2023 search, the following line was added to the Scopus search:

AND ORIG-LOAD-DATE AFT 20220527

**Web of Science run May 27, 2022 – N=1485**

(TS=("lumbar vertebra*" OR "lumbar spin*" OR "lumbar disk" OR "lumbar disc") OR TS=(lumbar NEAR/3 (stenosis OR stenotic)) OR TS=((lumbar OR lumbosacral OR "lumbo-sacral" OR "low* back*") NEAR/5 surg*) OR TS=(lumbago OR sciatica) OR (TS=(radiculopathy OR radiculitis OR "radicular pain*") OR TS=("nerve root*" NEAR/3 (pain* OR avulsion OR compress* OR disorder* OR pinch* OR inflam* OR imping* OR irritat* OR entrap* OR trap*))) AND TS=(back* OR lumbosacral OR "lumbo-sacral" OR lumbar)) AND (TS=((disc OR disk) NEAR/3 (replac* OR arthroplast*)) OR TS=((vertebra* OR spine OR spinal OR lumbar) NEAR/3 arthroplast*) OR TS=((disc OR discs OR disk*) NEAR/3 (remov* OR excis* OR surg* OR resect* OR replac*)) OR TS=((lumb* OR lamina) NEAR/3 (remov* OR excis* OR surg* OR resect* OR replac* OR arthroplast*)) OR TS=(("spin* fusion" OR (lumbar NEAR/2 fusion) OR alif OR plif OR tlif OR dlif OR xlif OR ollif OR axlif OR axialif OR lif OR arthrodesis OR syndesis OR spondylodesis OR spondylosyndesis OR "artificial ankylosis" OR discectom* OR diskectom* OR laminectom* OR laminotom* OR laminoplast* OR facetectom* OR foraminotom* OR fenestrat* OR microdiscectom* OR microdiskectom* OR accutherm OR disctrode OR spinecath OR transdiscal OR apld OR microdecompression OR "micro decompression" OR sequestrectom*)) OR TS=((surg* OR lumb* OR disc OR disk OR intradiscal) NEAR/3 decompress*) OR TS=(micro* NEAR/3 (surg* OR endoscop* OR laser)) OR TS=((disc OR disk OR intradiscal OR "intra discal" OR intervertebral OR "inter vertebral" OR percutaneous) NEAR/3 (arthroplast* OR biacuplast* OR annuloplast* OR electrothermal OR thermomodulation)) OR TS=(thermal NEAR/3 procedure*)) AND (TS=("range of motion" OR ROM) OR TS=(finger* NEAR/3 (floor OR toe OR knee OR "fibular head")) OR TS=(wrist crease NEAR/3 floor) OR TS=(Schober OR "straight leg raise*" OR SLR OR Lasegue* OR "isometric strength" OR "isokinetic strength" OR "motor control" OR "movement control" OR "functional movement screen*" OR FMS OR "sitting one leg knee extension" OR "posterior pelvic tilt" OR "waiter's bow" OR "one leg stance") OR TS=("gait analysis" OR "spatiotemporal gait" OR "spatio temporal gait" OR "stride length" OR "stride duration" OR "walking speed" OR "gait speed" OR cadence OR "gait asymmetry" OR "stance phase" OR "swing phase" OR "double limb support" OR "single limb support" OR inclinometer OR goniometer OR kyphometer OR "electromagnetic tracking") OR TS=(dynamometer OR "manual muscle test" OR MedX OR Cybex OR Kin-Com OR RehaGait OR JAMAR) OR TS=("aerobic capacity" OR VO2* OR "bicycle ergomet*" OR "maximal graded exercise*" OR "steep ramp" OR "muscle endurance" OR "Biering Sorensen" OR "modified Sorensen" OR "Roman chair") OR TS=(sternum NEAR/3 (ground OR floor)) OR TS=((back OR body OR trunk) NEAR/3 endurance) OR TS=("prone bridge") OR TS=(("lower extremities" OR (hips OR knees)) NEAR/5 (90deg* OR "90 degree*")) OR TS=(arch-up* OR sit-up* OR squat* OR "dumbbell press*" OR "double limb stance" OR "single limb stance" OR "stork stand*" OR "flamingo balance" OR y-balance OR CTSIB) OR TS=(("clinical test*" NEAR/2 "sensory interaction" NEAR/2 balance) OR ("clinical test*" NEAR/2 "sensory integration" NEAR/2 balance)) OR TS=(("Berg balance scale" OR Tinetti* OR "performance oriented mobility assessment*" OR "tandem walk*" OR "lower extremity motor coordination test*")) OR TS=(chair NEAR/3 (stand* OR rise*)) OR TS=("sit to stand" OR "stand up" OR "stand ups" OR roll*) OR TS=(lie NEAR/2 sit) OR TS=(bed NEAR/2 chair) OR TS=(step*) OR TS=((stand or standing) NEAR/2 continuous) OR TS=("functional capacity evaluation" OR lift* OR "progressive isoinertial lifting evaluation" OR pile OR "forward reach" OR "functional reach") OR TS=("walk test*" OR "self-paced walk*" OR "4-meter walk*" OR "4-metre walk*" OR "5-meter walk*" OR "5-metre walk*" OR "10-meter walk*" OR "10-metre walk*" OR "15-meter walk*" OR "15-metre walk*" OR "50-meter walk*" OR "50-metre walk*" OR "50-foot walk*" OR "5-minute walk*" OR "6-minute walk*" OR treadmill OR "overground walk*" OR "shuttle walk") OR TS=(walk NEAR/3 hall*) OR TS=(stair* NEAR/2 climb*) OR TS=("timed up and go" OR TUG OR "8 foot up and go" OR "Physical capability assessment tool" OR PCAT OR "aggregated functional performance test" OR "aggregated assessment of physical function" OR "short physical performance battery" OR "cumulated ambulation score" OR "functional independence measure" OR "Katz ADL index" OR "Katz index") OR TS=("Activity measure for post-acute care 6 clicks" OR "Activity measure for postacute care 6 clicks" OR "Physiotherapy functional mobility profile" OR "Barthel index") OR (TS=(lying) OR TS=(time NEAR/5 stand*) OR TS=("constant postures" OR "active postures" OR "sedentary postures" OR "sedentary activity" OR "walking time" OR "walking distance" OR "claudication index" OR "daily walking events" OR "light intensity" OR "moderate intensity" OR "vigorous intensity" OR "activity count" OR "gait cycles" OR "gait posture index" OR "physical activity" OR acceleromet*) OR TS=("wearable electronic devices" OR "fitness tracker*" OR "activity monitor*" OR pedomet* OR gps OR watch OR "smart watch") OR TS=(6wt NEAR/3 app*))) AND (TS=(instrumentation OR methods) OR TS=("validation study" OR "comparative study") OR TS=(psychometr* OR clinimetr* OR clinometr*) OR TS=("outcome assessment" OR "outcome measure*" OR "observer variation" OR "health status indicators") OR TS=(reproducib* OR "discriminant analysis" ) OR TI=(reliab* OR unreliab* OR valid* OR coefficient OR homogeneity OR homogeneous OR "internal consistency") OR AB=(reliab* OR unreliab* OR valid* OR coefficient OR homogeneity OR homogeneous OR "internal consistency") OR TI=((cronbach* AND (alpha OR alphas))) OR AB=((cronbach* AND (alpha OR alphas))) OR TI=(item AND (correlation* OR selection* OR reduction*)) OR AB=(item AND (correlation* OR selection* OR reduction*)) OR TS=(agreement OR precision OR imprecision OR precise values) OR TI=(test AND retest) OR AB=(test AND retest) OR TI=(stability OR interrater OR "inter-rater" OR intrarater OR "intra-rater" OR intertester OR "inter-tester" OR intratester OR "intra-tester" OR interobserver OR "inter-observer" OR intraobserver OR "intra-observer" OR intertechnician OR "inter-technician" OR intratechnician OR "intra-technician" OR interexaminer OR "inter-examiner" OR intraexaminer OR "intra-examiner" OR interassay OR "inter-assay" OR intraassay OR "intra-assay" OR interindividual OR "inter-individual" OR intraindividual OR "intra-individual" OR interparticipant OR "inter-participant" OR intraparticipant OR "intra-participant" OR kappa OR kappa's OR kappas) OR AB=(stability OR interrater OR "inter-rater" OR intrarater OR "intra-rater" OR intertester OR "inter-tester" OR intratester OR "intra-tester" OR interobserver OR "inter-observer" OR intraobserver OR "intra-observer" OR intertechnician OR "inter-technician" OR intratechnician OR "intra-technician" OR interexaminer OR "inter-examiner" OR intraexaminer OR "intra-examiner" OR interassay OR "inter-assay" OR intraassay OR "intra-assay" OR interindividual OR "inter-individual" OR intraindividual OR "intra-individual" OR interparticipant OR "inter-participant" OR intraparticipant OR "intra-participant" OR kappa OR kappa's OR kappas) OR TS=(repeatab*) OR TS=((replicab* OR repeated) AND (measure OR measures OR findings OR result OR results OR test OR tests)) OR TI=(generaliza* OR generalisa* OR concordance) OR AB=(generaliza* OR generalisa* OR concordance) OR TI=(intraclass AND correlation) OR AB=(intraclass AND correlation) OR TI=((discriminative OR "known group" OR "factor analysis" OR "factor analyses" OR "factor structure" OR "factor structures" OR dimension* OR subscale*)) OR AB=((discriminative OR "known group" OR "factor analysis" OR "factor analyses" OR "factor structure" OR "factor structures" OR dimension* OR subscale*)) OR TI=((multitrait AND scaling AND (analysis OR analyses))) OR AB=((multitrait AND scaling AND (analysis OR analyses))) OR TI=(("item discriminant" OR "interscale correlation*" OR error OR errors OR "individual variability" OR "interval variability" OR "rate variability")) OR AB=(("item discriminant" OR "interscale correlation*" OR error OR errors OR "individual variability" OR "interval variability" OR "rate variability")) OR TI=((variability AND (analysis OR values))) OR AB=((variability AND (analysis OR values))) OR TI=((uncertainty AND (measurement OR measuring))) OR AB=((uncertainty AND (measurement OR measuring))) OR TI=((sensitiv* OR responsive*)) OR AB=((sensitiv* OR responsive*)) OR TI=((limit AND detection)) OR AB=((limit AND detection)) OR TI=(("minimal detectable concentration" OR interpretab*)) OR AB=(("minimal detectable concentration" OR interpretab*)) OR TI=(((minimal OR minimally OR clinical OR clinically) AND (important OR significant OR detectable) AND (change OR difference))) OR AB=(((minimal OR minimally OR clinical OR clinically) AND (important OR significant OR detectable) AND (change OR difference))) OR TI=((small* AND (real OR detectable) AND (change OR difference))) OR AB=((small* AND (real OR detectable) AND (change OR difference))) OR TI=("meaningful change" OR "ceiling effect" OR "floor effect" OR "item response model" OR IRT OR Rasch OR "differential item functioning" OR DIF OR "computer adaptive testing" OR "item bank" OR "cross-cultural equivalence") OR AB=("meaningful change" OR "ceiling effect" OR "floor effect" OR "item response model" OR IRT OR Rasch OR "differential item functioning" OR DIF OR "computer adaptive testing" OR "item bank" OR "cross-cultural equivalence"))

For July 10, 2023 search, the following line was added to the Scopus search:

AND LD=(2022-05-27/2023-07-10)

**Health and Psychosocial Instruments (Ovid) run May 27, 2022 – N=10**

1 (lumbar or spine or spinal or disc or disk or lumbosacral or lumbo-sacral or back* or lumbago or sciatica or stenosis or stenotic).mp,hw. 5089

2 surg*.mp,hw. 1997

3 (fusion or arthrodesis or syndesis or spondylodesis or spondylosyndesis or artificial ankylosis or discectom* or diskectom* or laminectom* or laminotom* or laminoplast* or facetectom* or foraminotom* or fenestrat* or microdiscectom* or microdiskectom* or accutherm or disctrode or spinecath or transdiscal or decompress* or microdecompression or micro decompression or sequestrectom* or arthroplast*).mp,hw. 195

4 or/2-3 2150

5 1 and 4 97

6 (instrumentation or methods or validation study or comparative study or psychometr* or clinimetr* or clinometr* or outcome assessment or outcome measure* or observer variation or health status indicators or reproducib* or discriminant analysis or reliab* or unreliab* or valid* or coefficient or homogeneity or homogeneous or internal consistency or stability or interrater or inter-rater or intrarater or intra-rater or intertester or inter-tester or intratester or intra-tester or interobserver or inter-observer or intraobserver or intra-observer or intertechnician or inter-technician or intratechnician or intra-technician or interexaminer or inter-examiner or intraexaminer or intra-examiner or interassay or inter-assay or intraassay or intra-assay or interindividual or inter-individual or intraindividual or intra-individual or interparticipant or inter-participant or intraparticipant or intra-participant or kappa or kappa's or kappas or repeatab* or generaliza* or generalisa* or concordance or discriminative or known group or factor analysis or factor analyses or factor structure or factor structures or dimension* or subscale* or item discriminant or interscale correlation* or error or errors or individual variability or interval variability or rate variability or sensitiv* or responsive* or minimal detectable concentration or interpretab* or meaningful change or ceiling effect or floor effect or item response model or IRT or Rasch or differential item functioning or DIF or computer adaptive testing or item bank or cross-cultural equivalence).mp,va,ry. 39895

7 (cronbach* and (alpha or alphas)).mp,va,ry. 2549

8 (item and (correlation* or selection* or reduction*)).mp,va,ry. 511

9 (agreement or precision or imprecision or precise values).mp,va,ry. 1039

10 (test and retest).mp,va,ry. 2108

11 ((replicab* or repeated) and (measure or measures or findings or result or results or test or tests)).mp,va,ry. 129

12 (intraclass and correlation).mp,va,ry. 380

13 (multitrait and scaling and (analysis or analyses)).mp,va,ry. 0

14 (variability and (analysis or values)).mp,va,ry. 13

15 (uncertainty and (measurement or measuring)).mp,va,ry. 26

16 (limit and detection).mp,va,ry. 0

17 ((minimal or minimally or clinical or clinically) and (important or significant or detectable) and (change or difference)).mp,va,ry. 156

18 (small* and (real or detectable) and (change or difference)).mp,va,ry. 31

19 or/6-18 41003

20 5 and 19 10

For July 10, 2023 search, the following lines were added to the HAPI search:

21 ("202205" or "220206" or "220207" or "202208" or "202209" or "202210" or "202211" or "202212" or 2023*).up.

22 20 and 21

**ProQuest Dissertations & Theses run May 27, 2022 – N=53**

(((((noft("lumbar vertebra*") OR noft("lumbar spin*") OR noft("lumbar disk") OR noft("lumbar disc")) OR (noft(lumbar) NEAR/3 (noft(stenosis) OR noft(stenotic))) OR ((noft(lumbar) OR noft(lumbosacral) OR noft("lumbo-sacral") OR noft("low* back*")) NEAR/5 noft(surg*)) OR (noft(lumbago) OR noft(sciatica)) OR ((noft(radiculopathy) OR noft(radiculitis) OR noft("radicular pain*")) OR (noft("nerve root*") NEAR/3 (noft(pain*) OR noft(avulsion) OR noft(compress*) OR noft(disorder*) OR noft(pinch*) OR noft(inflam*) OR noft(imping*) OR noft(irritat*) OR noft(entrap*) OR noft(trap*)))) AND (noft(back*) OR noft(lumbosacral) OR noft("lumbo-sacral") OR noft(lumbar)))) AND (((noft(disc) OR noft(disk)) NEAR/3 (noft(replac*) OR noft(arthroplast*))) OR ((noft(vertebra*) OR noft(spine) OR noft(spinal) OR noft(lumbar)) NEAR/3 noft(arthroplast*)) OR ((noft(disc) OR noft(discs) OR noft(disk*)) NEAR/3 (noft(remov*) OR noft(excis*) OR noft(surg*) OR noft(resect*) OR noft(replac*))) OR ((noft(lumb*) OR noft(lamina)) NEAR/3 (noft(remov*) OR noft(excis*) OR noft(surg*) OR noft(resect*) OR noft(replac*) OR noft(arthroplast*))) OR ((noft("spin* fusion") OR (noft(lumbar) NEAR/2 noft(fusion)) OR noft(alif) OR noft(plif) OR noft(tlif) OR noft(dlif) OR noft(xlif) OR noft(ollif) OR noft(axlif) OR noft(axialif) OR noft(lif) OR noft(arthrodesis) OR noft(syndesis) OR noft(spondylodesis) OR noft(spondylosyndesis) OR noft("artificial ankylosis") OR noft(discectom*) OR noft(diskectom*) OR noft(laminectom*) OR noft(laminotom*) OR noft(laminoplast*) OR noft(facetectom*) OR noft(foraminotom*) OR noft(fenestrat*) OR noft(microdiscectom*) OR noft(microdiskectom*) OR noft(accutherm) OR noft(disctrode) OR noft(spinecath) OR noft(transdiscal) OR noft(apld) OR noft(microdecompression) OR noft("micro decompression") OR noft(sequestrectom*))) OR ((noft(surg*) OR noft(lumb*) OR noft(disc) OR noft(disk) OR noft(intradiscal)) NEAR/3 noft(decompress*)) OR (noft(micro*) NEAR/3 (noft(surg*) OR noft(endoscop*) OR noft(laser))) OR ((noft(disc) OR noft(disk) OR noft(intradiscal) OR noft("intra discal") OR noft(intervertebral) OR noft("inter vertebral") OR noft(percutaneous)) NEAR/3 (noft(arthroplast*) OR noft(biacuplast*) OR noft(annuloplast*) OR noft(electrothermal) OR noft(thermomodulation))) OR (noft(thermal) NEAR/3 noft(procedure*)))) AND ((noft(instrumentation) OR noft(methods)) OR (noft("validation study") OR noft("comparative study")) OR (noft(psychometr*) OR noft(clinimetr*) OR noft(clinometr*)) OR (noft("outcome assessment") OR (noft("outcome measure") OR noft("outcome measures")) OR noft("observer variation") OR noft("health status indicators")) OR (noft(reproducib*) OR noft("discriminant analysis") ) OR (noft(reliab*) OR noft(unreliab*) OR noft(valid*) OR noft(coefficient) OR noft(homogeneity) OR noft(homogeneous) OR noft("internal consistency")) OR (noft(reliab*) OR noft(unreliab*) OR noft(valid*) OR noft(coefficient) OR noft(homogeneity) OR noft(homogeneous) OR noft("internal consistency")) OR ((noft(cronbach*) AND (noft(alpha) OR noft(alphas)))) OR ((noft(cronbach*) AND (noft(alpha) OR noft(alphas)))) OR (noft(item) AND (noft(correlation*) OR noft(selection*) OR noft(reduction*))) OR (noft(item) AND (noft(correlation*) OR noft(selection*) OR noft(reduction*))) OR (noft(agreement) OR noft(precision) OR noft(imprecision) OR noft(precise values)) OR (noft(test) AND noft(retest)) OR (noft(test) AND noft(retest)) OR (noft(stability) OR noft(interrater) OR noft("inter-rater") OR noft(intrarater) OR noft("intra-rater") OR noft(intertester) OR noft("inter-tester") OR noft(intratester) OR noft("intra-tester") OR noft(interobserver) OR noft("inter-observer") OR noft(intraobserver) OR noft("intra-observer") OR noft(intertechnician) OR noft("inter-technician") OR noft(intratechnician) OR noft("intra-technician") OR noft(interexaminer) OR noft("inter-examiner") OR noft(intraexaminer) OR noft("intra-examiner") OR noft(interassay) OR noft("inter-assay") OR noft(intraassay) OR noft("intra-assay") OR noft(interindividual) OR noft("inter-individual") OR noft(intraindividual) OR noft("intra-individual") OR noft(interparticipant) OR noft("inter-participant") OR noft(intraparticipant) OR noft("intra-participant") OR noft(kappa) OR noft(kappa's) OR noft(kappas)) OR (noft(stability) OR noft(interrater) OR noft("inter-rater") OR noft(intrarater) OR noft("intra-rater") OR noft(intertester) OR noft("inter-tester") OR noft(intratester) OR noft("intra-tester") OR noft(interobserver) OR noft("inter-observer") OR noft(intraobserver) OR noft("intra-observer") OR noft(intertechnician) OR noft("inter-technician") OR noft(intratechnician) OR noft("intra-technician") OR noft(interexaminer) OR noft("inter-examiner") OR noft(intraexaminer) OR noft("intra-examiner") OR noft(interassay) OR noft("inter-assay") OR noft(intraassay) OR noft("intra-assay") OR noft(interindividual) OR noft("inter-individual") OR noft(intraindividual) OR noft("intra-individual") OR noft(interparticipant) OR noft("inter-participant") OR noft(intraparticipant) OR noft("intra-participant") OR noft(kappa) OR noft(kappa's) OR noft(kappas)) OR (noft(repeatab*)) OR ((noft(replicab*) OR noft(repeated)) AND (noft(measure) OR noft(measures) OR noft(findings) OR noft(result) OR noft(results) OR noft(test) OR noft(tests))) OR (noft(generaliza*) OR noft(generalisa*) OR noft(concordance)) OR (noft(generaliza*) OR noft(generalisa*) OR noft(concordance)) OR (noft(intraclass) AND noft(correlation)) OR (noft(intraclass) AND noft(correlation)) OR ((noft(discriminative) OR noft("known group") OR noft("factor analysis") OR noft("factor analyses") OR noft("factor structure") OR noft("factor structures") OR noft(dimension*) OR noft(subscale*))) OR ((noft(discriminative) OR noft("known group") OR noft("factor analysis") OR noft("factor analyses") OR noft("factor structure") OR noft("factor structures") OR noft(dimension*) OR noft(subscale*))) OR ((noft(multitrait) AND noft(scaling) AND (noft(analysis) OR noft(analyses)))) OR ((noft(multitrait) AND noft(scaling) AND (noft(analysis) OR noft(analyses)))) OR ((noft("item discriminant") OR noft("interscale correlation*") OR noft(error) OR noft(errors) OR noft("individual variability") OR noft("interval variability") OR noft("rate variability"))) OR ((noft("item discriminant") OR noft("interscale correlation*") OR noft(error) OR noft(errors) OR noft("individual variability") OR noft("interval variability") OR noft("rate variability"))) OR ((noft(variability) AND (noft(analysis) OR noft(values)))) OR ((noft(variability) AND (noft(analysis) OR noft(values)))) OR ((noft(uncertainty) AND (noft(measurement) OR noft(measuring)))) OR ((noft(uncertainty) AND (noft(measurement) OR noft(measuring)))) OR ((noft(sensitiv*) OR noft(responsive*))) OR ((noft(sensitiv*) OR noft(responsive*))) OR ((noft(limit) AND noft(detection))) OR ((noft(limit) AND noft(detection))) OR ((noft("minimal detectable concentration") OR noft(interpretab*))) OR ((noft("minimal detectable concentration") OR noft(interpretab*))) OR (((noft(minimal) OR noft(minimally) OR noft(clinical) OR noft(clinically)) AND (noft(important) OR noft(significant) OR noft(detectable)) AND (noft(change) OR noft(difference)))) OR (((noft(minimal) OR noft(minimally) OR noft(clinical) OR noft(clinically)) AND (noft(important) OR noft(significant) OR noft(detectable)) AND (noft(change) OR noft(difference)))) OR ((noft(small*) AND (noft(real) OR noft(detectable)) AND (noft(change) OR noft(difference)))) OR ((noft(small*) AND (noft(real) OR noft(detectable)) AND (noft(change) OR noft(difference)))) OR (noft("meaningful change") OR noft("ceiling effect") OR noft("floor effect") OR noft("item response model") OR noft(IRT) OR noft(Rasch) OR noft("differential item functioning") OR noft(DIF) OR noft("computer adaptive testing") OR noft("item bank") OR noft("cross-cultural equivalence")) OR (noft("meaningful change") OR noft("ceiling effect") OR noft("floor effect") OR noft("item response model") OR noft(IRT) OR noft(Rasch) OR noft("differential item functioning") OR noft(DIF) OR noft("computer adaptive testing") OR noft("item bank") OR noft("cross-cultural equivalence")))) AND ((noft("range of motion") OR noft(ROM)) OR (noft(finger*) NEAR/3 (noft(floor) OR noft(toe) OR noft(knee) OR noft("fibular head"))) OR (noft("wrist crease") NEAR/3 noft(floor)) OR (noft(Schober) OR noft("straight leg raise*") OR noft(SLR) OR noft(Lasegue*) OR noft("isometric strength") OR noft("isokinetic strength") OR noft("motor control") OR noft("movement control") OR noft("functional movement screen*") OR noft(FMS) OR noft("sitting one leg knee extension") OR noft("posterior pelvic tilt") OR noft("waiter's bow") OR noft("one leg stance")) OR (noft(gait) OR noft("stride length") OR noft("stride duration") OR noft(walk*) OR noft(cadence) OR noft("stance phase") OR noft("swing phase") OR noft("double limb support") OR noft("single limb support") OR noft(inclinometer) OR noft(goniometer) OR noft(kyphometer) OR noft("electromagnetic tracking")) OR (noft(dynamometer) OR noft("manual muscle test") OR noft(MedX) OR noft(Cybex) OR noft(Kin-Com) OR noft(RehaGait) OR noft(JAMAR)) OR (noft("aerobic capacity") OR noft(VO2*) OR noft("bicycle ergomet*") OR noft("maximal graded exercise*") OR noft("steep ramp") OR noft("muscle endurance") OR noft("Biering Sorensen") OR noft("modified Sorensen") OR noft("Roman chair")) OR (noft(sternum) NEAR/3 (noft(ground) OR noft(floor))) OR ((noft(back) OR noft(body) OR noft(trunk)) NEAR/3 noft(endurance)) OR (noft("prone bridge")) OR ((noft("lower extremities") OR (noft(hips) OR noft(knees))) NEAR/5 (noft(90deg*) OR noft("90 degree*"))) OR (noft(arch-up*) OR noft(sit-up*) OR noft(squat*) OR noft("dumbbell press*") OR noft("double limb stance") OR noft("single limb stance") OR noft("stork stand*") OR noft("flamingo balance") OR noft(y-balance) OR noft(CTSIB)) OR ((noft("clinical test*") NEAR/2 noft("sensory interaction") NEAR/2 noft(balance)) OR (noft("clinical test*") NEAR/2 noft("sensory integration") NEAR/2 noft(balance))) OR ((noft("Berg balance scale") OR noft(Tinetti*) OR noft("performance oriented mobility assessment*") OR noft("lower extremity motor coordination test*"))) OR (noft(chair) NEAR/3 (noft(stand*) OR noft(rise*))) OR (noft("sit to stand") OR noft("stand up") OR noft("stand ups") OR noft(roll*)) OR (noft(lie) NEAR/2 noft(sit)) OR (noft(bed) NEAR/2 noft(chair)) OR (noft(step*)) OR ((noft(stand) or noft(standing)) NEAR/2 noft(continuous)) OR (noft("functional capacity evaluation") OR noft(lift*) OR noft("progressive isoinertial lifting evaluation") OR noft(pile) OR noft("forward reach") OR noft("functional reach")) OR noft(treadmill) OR (noft(stair*) NEAR/2 noft(climb*)) OR (noft("timed up and go") OR noft(TUG) OR noft("8 foot up and go") OR noft("Physical capability assessment tool") OR noft(PCAT) OR noft("aggregated functional performance test") OR noft("aggregated assessment of physical function") OR noft("short physical performance battery") OR noft("cumulated ambulation score") OR noft("functional independence measure") OR noft("Katz ADL index") OR noft("Katz index")) OR (noft("Activity measure for post-acute care 6 clicks") OR noft("Activity measure for postacute care 6 clicks") OR noft("Physiotherapy functional mobility profile") OR noft("Barthel index")) OR ((noft(lying)) OR (noft(time) NEAR/5 noft(stand*)) OR (noft("constant postures") OR noft("active postures") OR noft("sedentary postures") OR noft("sedentary activity") OR noft("claudication index") OR noft("light intensity") OR noft("moderate intensity") OR noft("vigorous intensity") OR noft("activity count") OR noft("physical activity") OR noft(acceleromet*)) OR (noft("wearable electronic devices") OR noft("fitness tracker*") OR noft("activity monitor*") OR noft(pedomet*) OR noft(gps) OR noft(watch) OR noft("smart watch")) OR (noft(6wt) NEAR/3 noft(app*))))

For July 10, 2023 search, the following filter was added to the ProQuest search:

Applied filter: 2022-05-27 - 2023-07-10
