# Supplementary material for: Physical functioning in the lumbar spinal surgery population: A systematic review and narrative synthesis of outcome measures and measurement properties of the physical measures
Source: PLoS One. 2024 Aug 29;19(8):e0307004. doi: 10.1371/journal.pone.0307004 (PMC11361614; doi:10.1371/journal.pone.0307004)
Supplement: S2 Appendix — (DOCX) [file pone.0307004.s002.docx]

**S2 Appendix:** Rating criteria for measurement properties

| **Measurement property** | **Rating** | **Criteria for rating** |
| --- | --- | --- |
| Reliability | + | ICC or weighted Kappa ≥ 0.70 |
|  | ? | ICC or weighted Kappa not reported |
|  | - | ICC or weighted Kappa < 0.70 |
| Measurement error | + | LOA or SEM < MIC |
|  | ? | MIC not defined |
|  | - | LOA or SEM > MIC |
| Construct validity (hypothesis testing) | + | Result in accordance with hypothesis |
|  | ? | Hypothesis not defined and not able to derive |
|  | - | Result not in accordance with hypothesis |
| Criterion validity | + | Correlation or AUC ≥ 0.70 |
|  | ? | Not all information for ‘+’ reported |
|  | - | Correlation or AUC < 0.70 |
| Responsiveness | + | Result in accordance with hypothesis or AUC ≥ 0.70 |
|  | ? | Hypothesis not defined and not able to derive |
|  | - | Result not in accordance with hypothesis or AUC < 0.70 |

Criteria based on Prinsen, et al^1^

AUC, area under curve; ICC, intraclass correlation coefficient; LOA, limits of agreement; MIC, minimal important change

**References**

1. Prinsen CAC, Mokkink LB, Bouter LM, et al. COSMIN guideline for systematic reviews of patient-reported outcome measures. *Quality of Life Research*. 2018;27:1147-1157. doi:10.1007/S11136-018-1798-3
