# Supplementary material for: Physical functioning in the lumbar spinal surgery population: A systematic review and narrative synthesis of outcome measures and measurement properties of the physical measures
Source: PLoS One. 2024 Aug 29;19(8):e0307004. doi: 10.1371/journal.pone.0307004 (PMC11361614; doi:10.1371/journal.pone.0307004)
Supplement: S3 Appendix — (DOCX) [file pone.0307004.s003.docx]

**S3 Appendix: Articles excluded at full text stage (Stage 2)**

| **Not measurement property** | | | | |
| --- | --- | --- | --- | --- |
| **Number** | **Authors** | **Title** | **Journal** | **Year** |
| 1 | Zeitlberger, Anna M; Sosnova, Marketa; Ziga, Michal; Regli, Luca; Bozinov, Oliver; Weyerbrock, Astrid; Stienen, Martin N; Maldaner, Nicolai | Assessment of the Minimum Clinically Important Difference in the Smartphone-based 6-minute Walking Test After Surgery for Lumbar Degenerative Disc Disease. | Spine | 2021 |
| 2 | Jansson, K-A; Nemeth, G; Granath, F; Jonsson, B; Blomqvist, P | Health-related quality of life in patients before and after surgery for a herniated lumbar disc. | The Journal of bone and joint surgery. British volume | 2005 |
| 3 | Gautschi O.P.; Stienen M.N.; Corniola M.V.; Joswig H.; Schaller K.; Hildebrandt G.; Smoll N.R. | Assessment of the minimum clinically important difference in the timed up and go (TUG) test after surgery for lumbar degenerative disc disease | Swiss Medical Weekly | 2016 |
| 4 | Maldaner, Nicolai; Sosnova, Marketa; Ziga, Michal; Zeitlberger, Anna M.; Bozinov, Oliver; Gautschi, Oliver P.; Weyerbrock, Astrid; Regli, Luca; Stienen, Martin N. | External Validation of the Minimum Clinically Important Difference in the Timed-up-and-go Test After Surgery for Lumbar Degenerative Disc Disease. | Spine | 2022 |
| 5 | Smeets, R; Koke, A; Lin, CW; Ferreira, M; Demoulin, C | Measures of Function in Low Back Pain/Disorders Low Back Pain Rating Scale (LBPRS), Oswestry Disability Index (ODI), Progressive Isoinertial Lifting Evaluation (PILE), Quebec Back Pain Disability Scale (QBPDS), and Roland-Morris Disability Questionnaire | Arthritis Care & Research | 2011 |
| 6 | Sullivan, Humbert G; Bobenmoyer, Robert L; Boland, Kevin M; Cerniglia, Molly M; McHugh, Vicki L; Born, Hayley L; Mathiason, Michelle A; Ladwig, Nicholas R | Physical capability outcomes after total disc replacement with ProDisc-L. | International journal of spine surgery | 2012 |
| 7 | Gautschi, Oliver P; Stienen, Martin N; Corniola, Marco V; Joswig, Holger; Schaller, Karl; Hildebrandt, Gerhard; Smoll, Nicolas R | Assessment of the Minimum Clinically Important Difference in the Timed Up and Go Test After Surgery for Lumbar Degenerative Disc Disease. | Neurosurgery | 2017 |
| 8 | Stienen, Martin N; Maldaner, Nicolai; Joswig, Holger; Corniola, Marco V; Bellut, David; Prommel, Peter; Regli, Luca; Weyerbrock, Astrid; Schaller, Karl; Gautschi, Oliver P | Objective functional assessment using the "Timed Up and Go" test in patients with lumbar spinal stenosis. | Neurosurgical focus | 2019 |
| 9 | Herno A, Airaksinen O, Saari T | Computed-tomography after laminectomy for lumbar spinal stenosis – Patients pain patterns, walking capacity, and subjective disability had no correlation with computed-tomography findings | SPINE | 1994 |
| 10 | Stienen M.N.; Maldaner N.; Sosnova M.; Joswig H.; Corniola M.V.; Regli L.; Hildebrandt G.; Schaller K.; Gautschi O.P. | Lower extremity motor deficits are underappreciated in patient-reported outcome measures: Added value of objective outcome measures | Neurospine | 2020 |
| 11 | Smuck, Matthew; Muaremi, Amir; Zheng, Patricia; Norden, Justin; Sinha, Aman; Hu, Richard; Tomkins-Lane, Christy | Objective measurement of function following lumbar spinal stenosis decompression reveals improved functional capacity with stagnant real-life physical activity. | The Spine Journal | 2018 |
| 12 | Byrnes, S Kimberly; Nuesch, Corina; Loske, Stefan; Leuenberger, Andrea; Scharen, Stefan; Netzer, Cordula; Mundermann, Annegret | Inertial Sensor-Based Gait and Attractor Analysis as Clinical Measurement Tool: Functionality and Sensitivity in Healthy Subjects and Patients With Symptomatic Lumbar Spinal Stenosis. | Frontiers in physiology | 2018 |
| 13 | Hartmann, Sebastian; Hegewald, Aldemar Andres; Tschugg, Anja; Neururer, Sabrina; Abenhardt, Michael; Thome, Claudius | Analysis of a performance-based functional test in comparison with the visual analog scale for postoperative outcome assessment after lumbar spondylodesis. | European Spine Journal | 2016 |
| 14 | Herno, A; Airaksinen, O; Saari, T | Computed tomography after laminectomy for lumbar spinal stenosis. Patients' pain patterns, walking capacity, and subjective disability had no correlation with computed tomography findings. | Spine | 1994 |
| 15 | Janssen, Esther R; Osong, Biche; van Soest, Johan; Dekker, Andre; van Meeteren, Nico L; Willems, Paul C; Punt, Ilona M | Exploring Associations of Preoperative Physical Performance With Postoperative Outcomes After Lumbar Spinal Fusion: A Machine Learning Approach. | Archives of physical medicine and rehabilitation | 2021 |
| 16 | Yagi M.; Okada E.; Nori S.; Suzuki S.; Tsuji O.; Nagoshi N.; Nakamura M.; Matsumoto M.; Watanabe K. | How preoperative motor weakness affects the extent of recovery after elective spine surgery in patients with degenerative lumbar spinal stenosis | Spine Journal | 2021 |
| 17 | Inoue, Masahiro; Orita, Sumihisa; Inage, Kazuhide; Suzuki, Miyako; Fujimoto, Kazuki; Shiga, Yasuhiro; Kanamoto, Hirohito; Abe, Koki; Kinoshita, Hideyuki; Norimoto, Masaki; Umimura, Tomotaka; Sato, Takashi; Sato, Masashi; Suzuki, Masahiro; Enomoto, Keigo; Eguchi, Yawara; Akazawa, Tsutomu; Aoki, Yasuchika; Kawasaki, Yohei; Ohtori, Seiji | Objective evaluation of postoperative changes in real-life activity levels in the postoperative course of lumbar spinal surgery using wearable trackers. | BMC musculoskeletal disorders | 2020 |
| 18 | Wolfla, C | Assessment of the Minimum Clinically Important Difference in the Timed Up and Go Test After Surgery for Lumbar Degenerative Disc Disease COMMENT | NEUROSURGERY | 2017 |
| 19 | Iversen MD, Katz JN | Examination findings and self-reported walking capacity in patients with lumbar spinal stenosis. | Phys Ther | 2001 |
| 20 | Sosnova M, Zeitlberger AM, Ziga M, Gautschi OP, Weyerbrock A, Stienen MN, et al. | Longitudinal smartphone-based self-assessment of objective functional impairment in patients undergoing surgery for lumbar degenerative disc disease: initial experience. | Acta Neurochir | 2020 |

| **Population** | | | | |
| --- | --- | --- | --- | --- |
| **Number** | **Authors** | **Title** | **Journal** | **Year** |
| 1 | Ahmad, Hasan S; Singh, Shikha; Jiao, Kenneth; Basil, Gregory W; Yang, Andrew I; Wang, Michael Y; Welch, William C; Yoon, Jang W | Data-driven phenotyping of preoperative functional decline patterns in patients undergoing lumbar decompression and lumbar fusion using smartphone accelerometry. | Neurosurgical focus | 2022 |
| 2 | Chok B; Tan SB | The relationship between measures of spinal mobility and impairment, functional limitation and disability for low back pain. | Physiotherapy Singapore | 2001 |
| 3 | Sharif Bidabadi, Shiva; Murray, Iain; Lee, Gabriel Yin Foo | Validation of foot pitch angle estimation using inertial measurement unit against marker-based optical 3D motion capture system. | Biomedical engineering letters | 2018 |
| 4 | Pratt, Roland K; Fairbank, Jeremy C T; Virr, Andrew | The reliability of the Shuttle Walking Test, the Swiss Spinal Stenosis Questionnaire, the Oxford Spinal Stenosis Score, and the Oswestry Disability Index in the assessment of patients with lumbar spinal stenosis. | Spine | 2002 |
| 5 | Ruiz F.K.; Bohl D.D.; Webb M.L.; Russo G.S.; Grauer J.N. | Oswestry Disability Index is a better indicator of lumbar motion than the Visual Analogue Scale | Spine Journal | 2014 |
| 6 | Fritz, JM; Erhard, RE; Delitto, A; Welch, WC; Nowakowski, PE | Preliminary results of the use of a two-stage treadmill test as a clinical diagnostic tool in the differential diagnosis of lumbar spinal stenosis | JOURNAL OF SPINAL DISORDERS | 1997 |
| 7 | Zheng, CF; Liu, YC; Hu, YC; Xia, Q; Miao, J; Zhang, JD; Zhang, K | Correlations of Japanese Orthopaedic Association Scoring Systems with Gait Parameters in Patients with Degenerative Spinal Diseases | ORTHOPAEDIC SURGERY | 2016 |
| 8 | Herno, A; Airaksinen, O; Saari, T; Pitkanen, M; Manninen, H; Suomalainen, O | Computed tomography findings 4 years after surgical management of lumbar spinal stenosis. No correlation with clinical outcome. | Spine | 1999 |
| 9 | Harris, Sean; Roddey, Toni; Shelton, Tamara; Bailey, Lane; Brewer, Wayne; Ellison, Jennifer; Wang, Wanyi; Gleeson, Peggy | The functional lumbar index: Validation of a novel clinical assessment tool for individuals with low back pain. | Musculoskeletal science & practice | 2022 |
| 10 | Campbell H; Rivero-Arias O; Johnston K; Gray A; Fairbank J; Frost H | Responsiveness of objective, disease-specific, and generic outcome measures in patients with chronic low back pain: an assessment for improving, stable, and deteriorating patients. | Spine | 2006 |
| 11 | Tomkins-Lane, CC; Battie, MC | Validity and Reproducibility of Self-report Measures of Walking Capacity in Lumbar Spinal Stenosis | SPINE | 2010 |
| 12 | Monie A.P.; Price R.I.; Lind C.R.P.; Singer K.P. | Assessing the clinical utility of combined movement examination in symptomatic degenerative lumbar spondylosis | Clinical Biomechanics | 2015 |
| 13 | Ramos C.M.M.; Romerio C.F.W.E.; Amaral R.; de Oliveira C.E.A.S.; de Rezende Pratali R. | Impact of the lumbar stiffness related to arthrodesis to functional disability | Coluna/ Columna | 2021 |
| 14 | Tarnanen, Sami; Neva, Marko H; Kautiainen, Hannu; Ylinen, Jari; Pekkanen, Liisa; Kaistila, Tiina; Vuorenmaa, Mirja; Hakkinen, Arja | The early changes in trunk muscle strength and disability following lumbar spine fusion. | Disability and rehabilitation | 2013 |
| 15 | Yukawa, Yasutsugu; Lenke, Lawrence G; Tenhula, Janet; Bridwell, Keith H; Riew, K Daniel; Blanke, Kathy | A comprehensive study of patients with surgically treated lumbar spinal stenosis with neurogenic claudication. | The Journal of bone and joint surgery | 2002 |
| 16 | Oestergaard, Lisa G; Nielsen, Claus V; Bunger, Cody E; Svidt, Karen; Christensen, Finn B | The effect of timing of rehabilitation on physical performance after lumbar spinal fusion: a randomized clinical study. | European spine journal | 2013 |
| 17 | Yukawa, Y; Lenke, LG; Tenhula, J; Bridwell, KH; Riew, KD; Blanke, K | A comprehensive study of patients with surgically treated lumbar spinal stenosis with neurogenic claudication | JBJS | 2002 |
| 18 | Dominick GM, Winfree KN, Pohlig RT, Papas MA | Physical activity assessment between consumer and research-grade accelerometers: a comparative study in free-living conditions | JMIR Mhealth Uhealth | 2016 |
| 19 | Fritz, J M; Erhard, R E; Delitto, A; Welch, W C; Nowakowski, P E | Preliminary results of the use of a two-stage treadmill test as a clinical diagnostic tool in the differential diagnosis of lumbar spinal stenosis. | Journal of spinal disorders | 1997 |
| 20 | Simmonds MJ, Olson SL, Jones S, Hussein T, Lee CE, Novy D, et al. | Psychometric characteristics and clinical usefulness of physical performance tests in patients with low back pain | Spine | 1998 |
| 21 | Lee CE, Simmonds MJ, Novy DM, Jones S | Self-reports and clinician measured physical function among patients with low back pain: a comparison | Arch Phys Med Rehabil. | 2001 |
| 22 | Conway J, Tomkins CC, Haig AJ | Walking assessment in people with lumbar spinal stenosis: capacity, performance, and self-report measures | Spine J | 2011 |
| 23 | Andrasinova, T. et al.. | Is there a correlation between degree of radiologic lumbar spinal stenosis and its clinical manifestation? | Clin. Spine Surg | 2018 |
| 24 | Conrad BP, Shokat MS, Abbasi AZ, Vincent HK, Seay A, Kennedy DJ | Associations of self-report measures with gait, range of motion and proprioception in patients with lumbar spinal stenosis. | Gait Posture | 2013 |
| 25 | Tosic L, Goldberger E, Maldaner N, et al.. J. | Normative data of a smartphone app–based 6-minute walking test, test-retest reliability, and content validity with patient-reported outcome measures | Neurosurg Spine | 2020 |
| 26 | Stienen MN, Gautschi OP, Staartjes VE, et al (from database search) | Reliability of the 6-minute walking test smartphone application. | J Neurosurg Spine | 2019 |
| 27 | Sánchez-Zuriaga D, López-Pascual J, Garrido-Jaén D, de Moya MFP, Prat-Pastor J | Reliability and validity of a new objective tool for low back pain functional assessment. | Spine | 2011 |
| 28 | Tomkins, C. C., Battie, M. C., Rogers, T., Jiang, H. & Petersen, S. | A criterion measure of walking capacity in lumbar spinal stenosis and its comparison with a treadmill protocol. | Spine | 2009 |
| 29 | Tomkins-Lane, C. C., Battie, M. C. & Macedo, L. G. | Longitudinal construct validity and responsiveness of measures of walking capacity in individuals with lumbar spinal stenosis. | The Spine Journal | 2014 |
| 30 | Jespersen AB, Gustafsson MEK, Jespersen AB, Gustafsson MEAK | Correlation between the Oswestry Disability Index and objective measurements of walking capacity and performance in patients with lumbar spinal stenosis: a systematic literature review. | Eur  Spine J | 2018 |
| 31 | Stienen MN, Gautschi OP, Staartjes VE, Maldaner N, Sosnova M, Ho AL, et al (from searching references) | Reliability of the 6-minute walking test smartphone application | J Neurosurg Spine | 2019 |
| 32 | Pryce R, Johnson M, Goytan M, Passmore S, Berrington N, Kriellaars D. | Relationship between ambulatory performance and self-rated disability in patients with lumbar spinal stenosis | Spine | 2012 |

| **Outcome measure** | | | | |
| --- | --- | --- | --- | --- |
| **Number** | **Authors** | **Title** | **Journal** | **Year** |
| 1 | Lehmann, T.R.; Brand, R.A.; Gorman, T.W.O. | A low-back rating scale | Spine | 1983 |
| 2 | Liu, Chien-Lin; Wang, Shih-Tien; Lin Cf, Chien-fu Jeff; Cheng, Cheng-Kung; Wu, Chih-Liang; Chang, Huann; Lo, Wai-Hee | Isokinetic evaluation of the ankle before and after surgical treatment of the lumbar disc herniation. | Clinical biomechanics | 1998 |
| 3 | Okoro, Tosan; Qureshi, Assad; Sell, Beulah; Sell, Philip | The accuracy of assessment of walking distance in the elective spinal outpatients setting. | European spine journal | 2010 |
| 4 | Stief F.; Meurer A.; Wienand J.; Rauschmann M.; Rickert M. | Effect of lumbar spinal fusion surgery on the association of self-report measures with objective measures of physical function | Gait and Posture | 2018 |
| 5 | Scheidt, Sebastian; Endres, Sandra; Gesicki, Marco; Hofmann, Ulf Krister | Using video rasterstereography and treadmill gait analysis as a tool for evaluating postoperative outcome after lumbar spinal fusion. | Gait & posture | 2018 |
| 6 | Takami, Masanari; Kawakami, Mamoru; Hashizume, Hiroshi; Tsutsui, Shunji; Oka, Hiroyuki; Shinozaki, Tomohiro; Iwasaki, Hiroshi; Yamada, Hiroshi | Psychometric Evaluation and External Validity of the Japanese Version of Lumbar Stiffness Disability Index. | Spine surgery and related research | 2022 |
| 7 | Murakami Y.; Morino T.; Hino M.; Misaki H.; Imai H.; Miura H. | A Scoring System for Anterior Longitudinal Ligament Ossification of the Lumbar Spine in Diffuse Idiopathic Skeletal Hyperostosis: Relationship Between the Extent of Ligament Ossification and the Range of Motion | Global Spine Journal | 2023 |

| **Insufficient information to evaluate measurement property (e.g., abstract, review article)** | | | | |
| --- | --- | --- | --- | --- |
| **Number** | **Authors** | **Title** | **Journal** | **Year** |
| 1 | Stienen M.N.; Maldaner N.; Sosnova M.; Zeitlberger A.M.; Ziga M.; Weyerbrock A.; Bozinov O.; Regli L.; Gautschi O.P. | External Validation of the Timed-Up-and-Go (TUG) Test as Measure of Objective Functional Impairment in Patients with Lumbar Degenerative Disc Disease | European Spine Journal | 2020 |
| 2 | Master H.; Pennings J.S.; Coronado R.A.; Robinette P.; Haug C.; Skolasky R.L.; Riley L.H.; Neuman B.J.; Cheng J.S.; Aaronson O.S.; Devin C.J.; Wegener S.; Archer K.R. | 287. Early postoperative physical activity predicts clinical improvement in disability 1 year following spine surgery | Spine Journal | 2020 |
| 3 | Bienstock D.M.; Shankar D.S.; Kim J.; Zubizarreta N.; Poeran J.; Bronson W.H.; Chaudhary S.B.; Iatridis J.C. | P49. Physical activity measures in lumbar laminectomy patients: a prospective comparison of fitness tracker measures versus patient-reported outcome measures | Spine Journal | 2020 |
| 4 | Nuesch C.; Netzer C.; Schraknepper A.; Loske S.; Scharen S.; Mundermann A. | Clinical improvement after decompression surgery reflects improved gait symmetry during a six minute walking test in patients with symptomatic lumbar spinal stenosis | Swiss Medical Weekly | 2017 |
| 5 | Hofmann U.K.; Endress S.; Gesicki M.; Walter C.; Scheidt S. | Using video rasterstereography and treadmill gait analysis as a tool for evaluating postoperative outcome after lumbar spinal fusion | European Spine Journal | 2017 |
| 6 | Gautschi O.P.; Joswig H.; Corniola M.V.; Smoll N.R.; Schaller K.; Hildebrandt G.; Stienen M.N. | The validity of the timed-up-and-go (TUG) test as an outcome measure in degenerative disc disease | Swiss Medical Weekly | 2016 |
| 7 | Stienen M.N.; Smoll N.R.; Joswig H.; Corniola M.V.; Schaller K.; Hildebrandt G.; Gautschi O.P. | Validation of the baseline severity stratification of objective functional impairment in lumbar degenerative disc disease | Swiss Medical Weekly | 2016 |
| 8 | Gautschi O.P.; Smoll N.R.; Corniola M.V.; Joswig H.; Hildebrandt G.; Schaller K.; Stienen M.N. | Validity and reliability of an objective measurement of functional impairment in lumbar degenerative spine disease: The timedup-and-go-test (tug-test) | European Spine Journal | 2015 |
| 9 | Archer K.; Wegener S.; Vanston S.; Bird M.L.; McGirt M.J.; Cheng J.S.; Aaronson O.S.; Devin C.J. | Patient-reported and performance-based outcome measures following spine surgery: A longitudinal analysis | Spine Journal | 2014 |
| 10 | Maldaner N.; Zeitlberger A.; Ziga M.; Bozinov O.; Regli L.; Stienen M.N. | Objective Functional Outcome Measures Expose Relevant Ceiling Effects Inherent to Subjective Patient- Reported Outcome Measures in Patients Undergoing Surgery for Lumbar Degenerative Disorders | Neurosurgery | 2023 |
| 11 | Smuck M.; Buman M.; Ith M.A.; Haskell W.; Kao M.-C.J. | Activity monitoring with accelerometry outperforms self-reported and laboratory assessments of function in patients with lumbar spinal stenosis | PM and R | 2013 |
| 12 | Smuck M.; Buman M.; Ith M.A.; Haskell W.; Kao M.-C.J. | Lumbar spinal stenosis decompression normalizes free-living physical activity impairment | Spine Journal | 2013 |
| 13 | Brede E.; Neblett R.; Mayer T.; Gatchel R. | The effect of lumbar surgery on the flexion-relaxation phenomenon and its responsiveness to interdisciplinary functional restoration | Spine Journal | 2011 |
| 14 | Chen Y.-A.; Chang J.-W.; Pao J.-L.; Hsu W.-L. | Relationship between lumbar spine range-of-motion and balance performance during forward reaching in patients with lumbar spinal stenosis following fusion surgery | Physiotherapy | 2015 |
| 15 | Daentzer D.; Venjakob E.; Schulz J.; Schwarze M. | Effect of microsurgical operations in the lumbar spine on segmental stability - An in vivo-study using upright-MRI | European Spine Journal | 2019 |
| 16 | Stienen M.N.; Maldaner N.; Joswig H.; Corniola M.V.; Bellut D.; Prommel P.; Regli L.; Weyerbrock A.; Schaller K.; Gautschi O.P. | Objective assessment of function and outcome in patients with lumbar spinal stenosis by the Timed-Up and Go test | European Spine Journal | 2019 |
| 17 | Smuck M.; Buman M.; Haskell W.; Masters B. | Activity changes following treatment of lumbar spinal stenosis | PM and R | 2012 |
| 18 | Budithi S.; Dhawan R.; Balain B.; Trivedi J.M.; Jaffray D.C. | Only walking matters | European Spine Journal | 2012 |
| 19 | Singh S.; Ahmad H.; Basil G.; Wang M.; Welch W.; Yoon J.; Jiao K. | Assessing Pre-operative Functional Decline in Lumbar Fusion and Lumbar Decompression Patients Through Smartphone-based Accelerometry | Journal of Neurosurgery | 2022 |
| 20 | Kandwal P.; Verma A.; Gowda A.K.; Ahuja K.; Mittal S. | Effect of surgical intervention on gait function in patients with symptomatic lumbar canal stenosis | Spine Journal | 2022 |
| 21 | Haddas R.; Barzilay Y. | Home-based functional outcome measurements for spine patients: validation and feasibility study | Spine Journal | 2022 |
| 22 | McGregor A.H.; Hughes S.P.F. | The potential use of spinal motion as a measure of surgical outcome | Journal of Back and Musculoskeletal Rehabilitation | 2004 |
| 23 | Kuittinen, Pekka; Sipola, Petri; Aalto, Timo Juhani; MÃ¤Ã¤ttÃ¤, Sara; Parviainen, Anita; Saari, Tapani; Sinikallio, Sanna; Savolainen, Sakari; Turunen, Veli; KrÃ¶ger, Heikki; Airaksinen, Olavi; Leinonen, Ville | Correlation of lateral stenosis in MRI with symptoms, walking capacity and EMG findings in patients with surgically confirmed lateral lumbar spinal canal stenosis. | BMC Musculoskeletal Disorders | 2014 |
| 24 | Yasar, Baris; Simsek, Serkan; Er, Uygur; Yigitkanli, Kazim; Eksioglu, Emel; Altug, Tibet; Belen, Deniz; Kars, Zafer H; Bavbek, Murad | Functional and clinical evaluation for the surgical treatment of degenerative stenosis of the lumbar spinal canal. | Journal of neurosurgery spine | 2009 |
| 25 | Hartmann S, Hegewald AA, Tschugg A, et al. | Analysis of a performance-based functional test in comparison with the visual analog scale for postoperative outcome assessment after lumbar spondylodesis. | Eur Spine J | 2016 |
| 26 | Rao PJ, Phan K, Maharaj MM, Pelletier MH, Walsh WR, Mobbs RJ | Accelerometers for objective evaluation of physical activity following spine surgery | J Clin Neurosci | 2016 |
| 27 | DeVine J, Norvell DC, Ecker E, Fourney DR, Vaccaro A, Wang J, Andersson G (From Mobbs 2016) | Evaluating the correlation and responsiveness of patient-reported pain with function and quality-of-life outcomes after spine surgery | Spine | 2011 |

| **Not English language** | | | | |
| --- | --- | --- | --- | --- |
| **Number** | **Authors** | **Title** | **Journal** | **Year** |
| 1 | GÃksel, Ferdi | Ä°ki ve Daha Ã‡ok Seviye Dejeneratif Lomber Spinal Stenoz TanÄ±sÄ±yla Dekompresyon ve Spinal Enstrumanla FÃ¼zyon YapÄ±lan HastalarÄ±n Fonksiyonel ve Radyolojik SonuÃ§larÄ± | PQDT - Global | 2011 |
