# Supplementary material for: Physical functioning in the lumbar spinal surgery population: A systematic review and narrative synthesis of outcome measures and measurement properties of the physical measures
Source: PLoS One. 2024 Aug 29;19(8):e0307004. doi: 10.1371/journal.pone.0307004 (PMC11361614; doi:10.1371/journal.pone.0307004)
Supplement: S5 Appendix — (DOCX) [file pone.0307004.s005.docx]

**S5 Appendix: Summary of stage one results**

| **Contents** | **Page number** |
| --- | --- |
| **S5 Appendix 1:** Patient reported outcome measures | 2 |
| **S5 Appendix 2:** Physical measures | 7 |

**S5 Appendix 1:** Patient reported outcome measures

Patient reported outcome measures of physical functioning categorized according to IMMPACT (Initiative on Methods, Measurement, and Pain Assessment in Clinical Trials) and OMERACT (Outcome Measures in Rheumatoid Arthritis Clinical Trials) Framework.^1^

| **General physical functioning / activity** | **Site specific physical functioning / activity** | **Disease specific physical functioning / activity** | **Pain related physical functioning / activity** | **Activities of daily living** |
| --- | --- | --- | --- | --- |
| PROMs: n=31  Study author developed questions: n=41 | PROMs: n=26  Study author developed questions: n=3 | PROMs: n=9  Study author developed questions: n=1 | PROMs: n=2  Study author developed questions: n=2 | PROMs: n=2  Study author developed questions: n=20 |
| *Physical activity*   - Activity Pattern Indicator - Compendium of Physical Activities scale - International Physical Activity Questionnaire   - Long form   - Short form - Paffenbarger Physical Activity and Exercise Index - Tegner Activity Scale - Physical Activity Scale - Physical Activity Scale for the Elderly - Study developed questions: n=11   *Health status / Quality of life*   - 15D   - Mobility dimension   - Usual activities dimension - EQ-5D (3L and 5L)   - Mobility dimension   - Usual activities dimension - Sickness impact profile (Physical dimension) - SF-36   - Physical component / health score   - Physical functioning domain - SF-12   - Physical component / health score   - Physical functioning domain - SF-8   - Physical component / health score   - Physical functioning domain - VR-36 (Physical component / health score) - VR-12 (Physical component / health score) - WHOQOL-BREF (Physical health domain)   *Functional status*   - PROMIS   - Physical function   - Physical health - WONCA (Daily activities domain) - Geriatric Locomotive Function Scale - Loco-check Questionnaire - Patient reported walking distance / duration - Study developed questions: n=7 for functioning, n=13 mobility / walking, n=3 usual activities, n=2 work   *Disability*   - Disability Rating Index - Study developed questions: n=5   *Patient identified functional limitations*   - Canadian Occupational Performance Measure - Patient Specific Functional Scale | *Low back and / or leg pain*   - Back Pain Functional Scale - COMI   - Function domain   - Disability domain - Dallas Pain Questionnaire   - Daily activity subscale   - Work / Leisure subscale - Hannover Functional Ability Questionnaire (FFbH-R) - JOA (Gait domain) - JOABPEQ   - Walking ability domain   - Lumbar function domain - Low Back Outcome Score - Modified Low Back Outcome Score - Low Back Pain Rating Scale (Disability domain) - Million score / scale / disability index - Quebec Back Pain Disability Scale - General Function Score - Lumbar Spine Outcomes Questionnaire (Functional disability domain) - Maine-Seattle Back Questionnaire - MODEMS spine outcome tool - Roland Morris Disability Questionnaire   - 24-item version   - 23-item version   - 24-item sub ‘leg pain’ for ‘back pain’   - For Sciatica - Waddell Disability Index - Prolo functional economic rating scale (Functional subscale) - Modified Prolo functional economic rating scale (Functional subscale) - Study developed questions: n=3   *Back / headache / facial pain*   - Chronic Pain Grade (Disability subscale) | *Spinal stenosis – related*   - Brigham Spinal Stenosis questionnaire (Physical function subscale) - Spinal Stenosis Measure / Questionnaire (Physical function domain) - Stucki questionnaire (Physical function domain) - Zurich Claudication Questionnaire   - Physical function subscale   - Walking distance as single item question - Neurogenic Claudication Outcome Score - Oxford Claudication Score (Physical function domain) - Study developed question: n=1   *Scoliosis*   - SRS-22 (Function domain) - SRS-30 (Function domain) | *Pain related disability, not linked to body region or condition/disease*   - Pain Disability Index - Pain Disability Questionnaire (Functional status component) - Study developed questions: n=2 | *Self-care*   - EQ-5D (Self-care dimension) - Study developed questions: n=7   *Activities of daily living*   - JOA (ADL domain) - Study developed questions: n=13 |

| **Study author developed physical functioning PROMs** | |
| --- | --- |
| **General physical functioning / activity** | |
| Physical activity | - Weekly frequency of low-intensity physical activity - Physical activity dose and intensity level: Seldom active (inactive); Irregular performance of low-intensity activity (mildly active); Regular performance of low-intensity activity (walking); Regular performance of moderate intensity activity (moderately active); Regular performance of moderate-to-high intensity activity (very active)) - Frequency of participation in 15 types of physical activity/exercise introduced in the Kenko Nippon 21 - Minutes per week of leisure time physical activity - 11 different leisure activities and the time spent on each activity per week. (eg golf, swimming, etc) - Based on Cincinatti Sports Activity Scale to understand the frequency and strenuousness of sporting activities. - Level of physical activity rated on a six-grade scale - Minutes per week of leisure time physical activities - Minutes per week of leisure time physical activities - During the past 4 weeks, what was the most strenuous level of physical activity that you could do? - physical activity and sport: regular, occasional, none |
| Functional status | Functioning   - Daily activity performance (1: no problems; 2: moderate problems; 3: extreme problems) No problems: I am able to perform main activity (eg, work, study, housework) with or without limited vigorous activities, such as running, lifting heavy objects, participating in strenuous sports; moderate problems: I am able to perform main activity with limited moderate activities, such as moving a table, pushing a vacuum cleaner, bowling, or playing golf; extreme problems: I am unable to perform main activity or I take sick leave. - Evaluate difficulty with sitting, standing, and sleeping - Evaluate functional outcomes on a 5-point scale: 1: Independence with respect to mobilization and activities of daily living and 5: Extreme limitations in walking or the need for major assistance with activities of daily living - Assess ability to stand, sit, walk and perform other activities of daily living on an analog scale ranging from 32 points (totally disabled) to 7 points (normal function, with contact sports) - Hindrance in daily activity (1: Every activity, 10: minimal activity only) - Sitting, standing duration - Stand up ability: unrestricted, restricted, almost impossible   Mobility / walking   - 1) Describe your mobility: No problems to walk; Some problems to walk; Confined to bed. - Self-described mobility - Mobility status (No problems in walking about; Some problems in walking about; Confined to bed) - Prior level of function based on level of assistance needed in mobility (fully independent, independent in community with limitation, and independent at household level with or without assistance) - Prior level of function based on the level of assistance needed in mobility (independent, partially dependent, and maximally dependent). - Walking difficulty (scale from none to maximum) - Walking capacity from 1 (best) to 5 (worst) - Walking capacity (ability to walk distances, ambulate indoors and walk for pleasure) - Ability to walk (normal gait, abnormal gait, use of cane/walker/wheelchair, or bedridden) - Walking capacity (difficulty walking in general, outdoors, shopping, around the house, bedroom to bathroom). scale ranges from 1 (least disabled) to 5 (most disabled). - Walking distance, walking difficulty, walking outdoors or in malls, and walking throughout the home (all rated on scale 1-5) - Lower extremity function (walking): 0=unable to walk; 1=needs aid to walk on flat ground; 2=needs aid to climb stairs, 3=walks unaided but with difficulty (includes painful walking); 4=walks normally - Gait ability: unrestricted, restricted walk perimeter, totally restricted   Usual activities   - Describe your ability to perform your usual activities (i.e. work, study, housework and leisure activities): No problems in performing usual activities; Some problems in performing usual activities; Unable to perform usual activities. 4) activity status: strenuous, moderate, sedentary, no activities - Ability to perform usual activities - Ability to perform usual activities; 3 responses: no problems, some problems, inability.   Work   - Ability to perform 1) normal activities and 2) work: 1) Excellent (no limitations); 2) Good (one or more minor limitations but most work or ADL can be accomplished); 3) Fair (one or more limitations that interfere seriously with ADL or work); and 4) Incapacitated (unable to perform ADL or to work at all) - Ability to perform work-related activities (0 points: Not limited; 1: Slightly limited; 2: substantially impaired; 3: severely limited or impossible |
| Disability | - Evaluate disability as quantified by the ability to perform employment and activities of daily living as normal: (0), slightly limited (1), significantly impaired (2), or severely limited/impossible (3) - Impairments in work, walk, stairs, sleep, sex, driving, sitting - Evaluate severity of disability, based on Korean severity index ranging from 1 (severe; ambulation dependent on aids or a wheelchair or patient bedridden) to 6 (mild; independent ambulation without aids) - Understand if patients felt impaired in the execution of 12 daily activities (dressing, rising from a chair without using the arms, washing-up, cleaning, shopping, driving a car, bicycling, bus riding, stair climbing, walking, sleeping, normal sexual activities). The number of impaired activities was recorded as a disability score. - Impairment in ADLS: work, walk, stairs, sleep, sex, drive, eat |
| **Site specific physical functioning / activity** | |
| Low back and / or leg pain | - Global back disability - Are you functioning better or worse than before your back surgery? - Developed questionnaire - Specific Functional Disability Index, based on Lumbar Stiffness Disability Questionnaire |
| **Disease specific physical functioning / activity** | |
| Spinal stenosis – related | - Standing time prior to being limited by neurogenic claudication symptoms |
| **Pain related physical functioning / activity** | |
| Pain related disability, not linked to body region or condition | - Community ambulation: Able to walk in the malls in pain or were unable to walk in the malls: No; Yes, always pain; Yes, sometimes pain; Yes, comfortably - Household ambulation: Able to walk from the bedroom to the bathroom in constant pain or were unable to get to the bathroom (No; Yes, always pain; Yes, sometimes pain; Yes, comfortably) |
| **Activities of daily living** | |
| Self care | - Describe your self-care: No problems with self-care; Facing difficulties in washing or dressing; Unable to wash or dress. - Ability to self-care - Ability to perform self-care activities. 3 responses: no problems, some problems, inability. - Prior level of function based on level of assistance needed in activities of daily living (fully independent, independent in community with limitation, and independent at household level with or without assistance) - Overall Functioning Rating: 0=bedridden; 1=capable of self care with aid; 2=capable of self-care alone; 3=capable of most desired activities; 4=capable of all desired activities - Limitations in dressing, rated as: none, mild, moderate, severe - Limitations in washing, rated as: none, mild, moderate, severe |
| Activities of daily living | - Patients selected two important ADL activities that were severely hampered by their symptoms, in a standardized fashion. These were called Main Complaints (MC) - Ability to perform 1) normal activities and 2) work: 1) Excellent (no limitations); 2) Good (one or more minor limitations but most work or ADL can be accomplished); 3) Fair (one or more limitations that interfere seriously with ADL or work); and 4) Incapacitated (unable to perform ADL or to work at all) - Ability to perform activities of daily living (0 = not at all and 10 = all activities) - ADLs: easy to perform, moderate restriction, very difficult to perform - Ability to perform activities of daily living (0 points: Not limited; 1: Slightly limited; 2: substantially impaired; 3: severely limited or impossible - At baseline, patients selected two important ADL activities that were severely hampered by their symptoms, in a standardized way, and severity was scored on a VAS. - Study developed questionnaire about the effects of lumbar stiffness after surgery on their ADL, based on Lumbar Stiffness Disability Questionnaire - Prior level of function based on the level of assistance needed in activities of daily living (independent, partially dependent, and maximally dependent). - Limitations in 3) getting out of bed, rated as: none, mild, moderate, severe - Restriction in daily activities: I am able to do my daily activities as desired; I have to modify my daily activities because of my back or leg pain; I am unable to perform my daily activities because of my back or leg pain - Evaluate functional outcomes on a 5-point scale: 1: Independence with respect to mobilization and activities of daily living and 5: Extreme limitations in walking or the need for major assistance with activities of daily living - Pain induced limitations while performing daily activities (i.e. dressing, washing, getting out of bed and walking) - Degree of return to preop ADL: Grade 1 (excellent, no limitations); Grade 2 (good, minor limitations with most ADL and work accomplished); Grade 3 (Severe limitations interfering with ADL or work capacities); Grade 4 (unable to perform ADL) |

**S5 Appendix 2:** Physical measures

Physical measures of physical functioning categorized according to level two categories of the International Classification of Functioning, Disability and Health (ICF) framework.^2^

| **Impairment-based physical measures** | | | | | | | | |
| --- | --- | --- | --- | --- | --- | --- | --- | --- |
| **ICF Classification** | | | | | | | **Physical outcome measure / physical measure (n=number of physical measures identified across studies)** | **Measurement Tool** |
| **Component** | | **Chapter** | | **Domain** | **Category** | |  |  |
| Body Function | | Neuromusculoskeletal and movement-related functions | | Functions of the joints and bones | b710:  Mobility of joint functions | | Range of movement (n=15)   - Active range of movement   - Thoracic spine   - Thoracolumbar spine   - Lumbar spine   - Lumbopelvic   - Hip   - Knee - Schober test - Modified Schober test - Distance   - Fingertip to floor   - Fingertip to toe   - Fingertip to knee   - Fingertip to fibular head   - Wrist crease to floor - Passive straight leg raise - Lasegue’s test | - Inclinometer - Goniometer - Kyphometer - Electromagnetic tracking |
|  |  |  |  | Muscle functions | b730:  Muscle power functions | | Strength (n=6)   - Maximum isometric strength:   - Trunk   - Lumbar spine   - Hip   - Knee   - Handgrip - Maximum isokinetic strength: Trunk | - Dynamometer - Manual muscle test - MedX lumbar extension device - Cybex, Kin-Com Isokinetic testing device - JAMAR handgrip dynamometer |
|  |  |  |  | Movement functions | b760:  Control of voluntary movement functions | | Motor control (n=5)   - Modified Functional Movement Screen - Study developed measures of ‘movement control’:   - Sitting one leg knee extension   - Standing posterior pelvic tilt   - Waiter’s bow   - One leg stance test |  |
|  |  |  |  |  | b770:  Gait pattern functions | | Gait parameters (n=9)   - Two Step Test - Stride length - Stride duration - Gait speed - Cadence - Asymmetry during stance phase - Asymmetry during swing phase - Asymmetry of double limb support - Asymmetry of single limb support | - RehabGait system with 7 inertial sensors strapped to legs |
| **Performance-based physical measures** | | | | | | | | |
| **ICF Classification** | | | | | | | **Physical outcome measure / physical measure (n=number of physical measures identified across studies)** | **Measurement Tool** |
| **Component** | **Chapter** | | **Domain** | | | **Category** |  |  |
| Body function | Functions of the cardiovascular, hematological, immunological and respiratory systems | | Additional functions and sensations of the cardiovascular and respiratory systems | | | b455 :  Exercise tolerance functions | Aerobic capacity (n=3)   - Bicycle ergometer test - Maximal graded exercise test - Steep ramp test | - Bicycle ergometer - Treadmill |
|  | Neuromusculoskeletal and movement-related functions | | Muscle functions | | | b740 :  Muscle endurance functions | Muscle endurance (n=9)   - Biering Sorensen test - Modified Sorensen test - Sustained positions:   - Prone hold sternum away from ground   - Prone bridge and hold   - Supine hips and knees at 90˚ and hold - Repetitions:   - Arch-ups   - Sit-ups   - Squats   - Single-arm dumbbell press | - Variable roman chair apparatus |
|  |  |  | Movement functions | | | b755:  Involuntary movement reaction functions | Balance (n=13)   - Double limb stance   - Eyes open   - Eyes closed   - Narrow base of support   - Wide base of support - Single limb stance   - Eyes open   - Eyes closed - Stork Stand test - Flamingo Balance Test - Y Balance test - Clinical Test for Sensory Interaction in Balance - Berg Balance Scale - Tinetti Test / Performance Oriented Mobility Assessment - Tandem walk | - *None* |
|  |  |  |  |  |  | b760:  Control of voluntary movement functions | Motor control (n=1)   - Lower Extremity Motor Coordination Test | - *None* |
| Activities and Participation | Mobility | | Changing and maintaining body position | | | d410: Changing basic body position | Functional mobility (n=13)   - Timed up and go - 8 foot up and go - Sit to stand / Chair stand test / Chair rise test:   - 5 repetitions   - Repetitions for 30 sec   - Repetitions for 60 sec - Stand Up Test - Time to perform:   - Roll   - Lie to sit   - Sit to stand   - Bed to chair - Alternate Step Test - Stepping repetitions / test - Four Square Step Test | - TUG phone application |
|  |  |  |  |  |  | d415: Maintaining body position | Sustained positions (n=1)   - Duration of time able to stand continuously | - *None* |
|  |  |  | Carrying, moving and handling objects | | | d430: Lifting and carrying objects | Lifting (n=4)   - Functional Capacity Evaluation - Lift waist to chest height: 1kg, 6kg - Progressive Isoinertial Lifting Evaluation test – Lumbar - Progressive Isoinertial Lifting Evaluation test – Cervical | - *None* |
|  |  |  |  |  |  | d445: Hand and arm use | Reaching (n=3)   - Forward reach test:   - Unloaded   - Loaded - Functional reach test | - *None* |
|  |  |  | Walking and moving | | | d450:  Walking | Walking (n=16)   - Usual gait speed walk test:   - 4 meters   - 5 meters   - 10 meters - Fast gait speed walk test:   - 50 foot   - 10 meters   - 15 meters   - 50 meters - 5 min walk test - 6 min walk test - Self-paced walking test - Treadmill test:   - Distance at fixed or preferred speed   - Time at fixed or preferred speed   - Modified Bruce Protocol - Overground walking distance - Overground walking time - Shuttle walking test | - Treadmill - 6WT phone application |
|  |  |  |  |  |  | d451:  Going up and down stairs | Stairs (n=3)   - Stair climb (ascend and descend):   - 4 steps   - 10 steps   - Maximum stairs in 1 minute | - *None* |
|  |  |  | Other mobility | | | d498: Other specified mobility | Multi-activity performance-based measures (n=3)   - Physical Capability Assessment Tool - Aggregated Functional Performance Test - Short Physical Performance Battery   Functional task performance (n=8)   - Cumulated Ambulation Score - Functional Independence Measure: Bed mobility, transfers, gait, self-care domains - Katz ADL index - Activity Measure for Post-Acute Care (AMPAC) 6 clicks - Physiotherapy Functional Mobility Profile - Barthel Index for Activities of Daily Living - Modified Barthel index - Karnofsky Performance Status Scale | - TUG app |
| **Activity in natural environment physical measures** | | | | | | | | |
| **ICF Classification** | | | | | | | **Physical outcome measure / physical measure (n=number of physical measures identified across studies)** | **Measurement Tool** |
| **Component** | **Chapter** | | **Domain** | | | **Category** |  |  |
| Activities and Participation | Mobility | | Changing and maintaining body position | | | d415:  Maintaining body position | Sustained positions (n=4)   - Time spent not moving:   - Lying   - Standing   - Constant postures   - Sedentary postures | - Accelerometers:   - McRobert’s DynaPort   - ActivPAL; ActivPAL3   - Unilever GENEA   - Apple watch - GPS |
|  |  |  | Walking and moving | | | d450:  Walking | Walking (n=4)   - Walking time - Walking distance - Walking speed - Claudication Index   Composite gait measure (n=1)   - Gait Posture Index | - Accelerometers   - Apple watch   - Fitbit Charge; Zip - GPS (Homefree wireless telehome care solutions) - Pedometer |
|  |  |  |  |  |  | d460:  Moving around in different location | Walking (n=1)   - Number of daily walking events out of home | - GPS |
|  |  |  | Other mobility | | | d498:  Other specified mobility | Physical activity parameters (n=12)   - Intensity, Time spent in   - Light intensity   - Moderate Intensity   - Moderate – vigorous intensity   - Vigorous intensity   - Consecutive minutes above light intensity activity - Volume:   - Activity counts   - Steps per day   - Gait cycles per day - Time spent moving:   - Active postures   - Physical activity duration per day - Achievement of physical activity guidelines | - Accelerometers:   - Actigraph GT3X+; GT3X-BT   - ActivPAL; ActivPAL3   - Fitbit Charge; Zip; Flex   - Jawbone Up Move   - Apple watch   - Garmin watch   - Xiaomi Mi Band   - StepWatch3   - Unilever GENEA - Pedometer: Omron HJ-113-E |

**References**

1. Taylor AM, Phillips K, Patel K V., et al. Assessment of physical function and participation in chronic pain clinical trials: IMMPACT/OMERACT recommendations. *Pain*. 2016;157(9):1836-1850. doi:10.1097/j.pain.0000000000000577

2. World Health Organization. *Towards a Common Language for Functioning, Disability and Health ICF*.; 2002.
