# Supplementary material for: Physical functioning in the lumbar spinal surgery population: A systematic review and narrative synthesis of outcome measures and measurement properties of the physical measures
Source: PLoS One. 2024 Aug 29;19(8):e0307004. doi: 10.1371/journal.pone.0307004 (PMC11361614; doi:10.1371/journal.pone.0307004)
Supplement: S6 Appendix — (DOCX) [file pone.0307004.s006.docx]

**S6 Appendix: Stage two results**

| **Contents** | **Page number** |
| --- | --- |
| **S6 Appendix 1:** Risk of bias | 2 |
| **S6 Appendix 2:** Summary of measurement properties | 18 |
| **S6 Appendix 3:** Hypotheses for validity and responsiveness (hypothesis testing approaches) | 47 |
| **S6 Appendix 4:** Elements of a comprehensive research question for reliability and measurement error of physical measures | 58 |
| **S6 Appendix 5:** Quality of evidence (GRADE) | 63 |
| **S6 Appendix 6:** Interpretability | 70 |
| **S6 Appendix 7:** Feasibility | 84 |
| **References** | 99 |

**S6 Appendix 1:** Risk of bias

| **Construct validity** | | | | | | |
| --- | --- | --- | --- | --- | --- | --- |
| **Physical outcome measure / physical measure** | **Study** | **Is it clear what the comparator instrument(s) measure(s)?** | **Were the measurement properties of the comparator instrument(s) sufficient?** | **Was the statistical method appropriate for the hypotheses to be tested?** | **Were there any other important flaws in the design or statistical methods of the study?** | **TOTAL (Lowest score)** |
| **Impairment-based physical outcome measures** | | | | | | |
| **Physical outcome measure: Active range of movement** | | | | | | |
| Physical measure: Lumbar, trunk and hip flexion and extension | Mannion et al (2005)^2^ | Very good | Doubtful: Some information reported in study population | Very good | Inadequate: No hypothesis reported | Inadequate |
| Physical measure: Lumbopelvic flexion and extension | Pitino (2000)^3^ | Very good | Very good | Very good | Inadequate: Incomplete hypothesis, Sample size <20 | Inadequate |
| **Physical outcome measure: Handgrip strength** | | | | | | |
| Physical measure: Maximum voluntary contraction | Kwon et al (2020)^4^ | Very good | Inadequate: No information reported | Very good | Inadequate: No hypothesis reported, Reporting of experimental protocol led to difficulties evaluating methodology | Inadequate |
|  | Kwon et al (2020)^5^ | Very good | Doubtful: Some information reported for some measures in any population | Adequate: Abstract and methods section state 2 different statistical methods - both appropriate but unclear which was used | Inadequate: No hypothesis reported, Reporting of experimental protocol led to difficulties evaluating methodology | Inadequate |
|  | Inoue et al (2020)^6^ | Very good | Inadequate: No information reported | Very good | Inadequate: No hypothesis reported, Reporting of experimental protocol led to difficulties evaluating methodology | Inadequate |
| **Physical outcome measure: Gait Parameters** | | | | | | |
| Physical measure: Two-test test | Fujita et al (2019)^7^ | Very good | Doubtful: Some information reported for some measures in any population | Adequate: Assumable because distribution of scores not presented | Inadequate: No hypothesis reported | Inadequate |
| **Performance-based physical outcome measures** | | | | | | |
| **Physical outcome measure: 5 repetitions sit to stand** | | | | | | |
| Physical measure: Time to complete | Master et al (2020)^8^ | Very good | Inadequate: No information reported | Very good | Inadequate: No hypothesis reported | Inadequate |
|  | Staartjes et al (2019)^9^ | Very good | Doubtful: Some information reported for some measures in any population | Very good | Inadequate: No hypothesis reported | Inadequate |
|  | Staartjes et al (2020)^10^ | Very good | Inadequate: No information reported | Very good | Inadequate: No hypothesis reported | Inadequate |
|  | Klukowska et al (2020)^11^ | Very good | Inadequate: No information reported | Very good | Inadequate: No hypothesis reported, Reporting of measurement time points unclear | Inadequate |
|  | Staartjes et al (2018)^12^ | Very good | Doubtful: Some information reported in any population | Very good | Inadequate: No hypothesis reported | Inadequate |
| **Physical outcome measure: 6-minute walk test** | | | | | | |
| Physical measure: Distance walked | Maldaner et al (2020)^13^ | Very good | Inadequate: No information reported | Very good | Inadequate: No hypothesis reported | Inadequate |
|  | Maldaner et al (2021)^14^ | Very good | Inadequate: No information reported | Very good | Inadequate: No hypothesis reported, Appears to have combined pre and post-operative scores for analysis despite some being statistically different | Inadequate |
|  | Zeitlberger et al (2022)^15^ | Very good | Inadequate: No information reported | Very good | Inadequate: No hypothesis reported, Inconsistent reporting of which measurement properties investigated and associated statistical analyses, Minimal reporting of standardized procedures for data collection | Inadequate |
|  | Ziga et al (2023)^16^ | Very good | Inadequate: No information reported | Very good | Inadequate: No hypothesis reported | Inadequate |
| Physical measure: Time to first symptoms | Zeitlberger et al (2022)^15^ | Very good | Inadequate: No information reported | Very good | Inadequate: No hypothesis reported, Inconsistent reporting of which measurement properties investigated and associated statistical analyses, Minimal reporting of standardized procedures for data collection | Inadequate |
| Physical measure: Distance to first symptoms | Zeitlberger et al (2022)^15^ | Very good | Inadequate: No information reported | Very good | Inadequate: No hypothesis reported, Inconsistent reporting of which measurement properties investigated and associated statistical analyses, Minimal reporting of standardized procedures for data collection | Inadequate |
| **Physical outcome measure: 10-meter walk test** | | | | | | |
| Physical measure: Walking speed | Wada et al (2022)^17^ | Very good | Very good | Very good | Inadequate: No hypothesis reported | Inadequate |
| **Physical outcome measure: Modified Sorensen test** | | | | | | |
| Physical measure: Time to exhaustion | Dedering et al (2006)^18^ | Very good | Doubtful: Some information reported for some measures | Very good | Inadequate: No hypothesis reported, Same investigator collected pre- and post-operative measures but no report of blinding of evaluator | Inadequate |
|  | Dedering (2012)^19^ | Very good | Inadequate: No information reported | Very good | Inadequate: No hypothesis reported | Inadequate |
| **Physical outcome measure: Timed Up and Go** | | | | | | |
| Physical measure: Time to complete | Maldaner et al (2021)^14^ | Very good | Inadequate: No information reported | Very good | Inadequate: No hypothesis reported, Appears to have combined pre and post- operative scores for analysis despite some being statistically different, | Inadequate |
|  | Stienen et al (2021)^20^ | Very good | Doubtful: Some information reported in any population | Very good | Doubtful: Non-discriminative hypotheses, Inconsistent reporting of which measurement properties investigated and associated statistical analyses | Doubtful |
|  | Staartjes et al (2022)^21^ | Very good | Doubtful: Some information reported in a surgical population | Very good | Inadequate: No hypothesis reported, pre-operative assessment completed at 2 different time points (outpatient consultation, admission for surgical treatment) but did not report time difference | Inadequate |
|  | Master et al (2020)^8^ | Very good | Inadequate: No information reported | Very good | Inadequate: No hypothesis reported | Inadequate |
|  | Corniola et al (2016)^22^ | Very good | Inadequate: No information reported | Very good | Inadequate: No hypothesis reported, Minimal reporting of standardized procedures for data collection | Inadequate |
|  | Gautschi et al (2016)^23^ | Very good | Inadequate: No information reported | Very good | Inadequate: No hypothesis reported, Unclear reporting of assessment time points | Inadequate |
|  | Stienen et al (2017)^24^ | Very good | Doubtful: Some information reported in any population | Very good | Inadequate: No hypothesis reported | Inadequate |
| **Physical outcome measure: Treadmill test** | | | | | | |
| Physical measure: Maximum walking distance | Herno et al (1999)^25^ | Very good | Inadequate: No information reported | Very good | Inadequate: Incomplete hypothesis | Inadequate |
|  | Prasad et al (2016)^26^ | Very good | Inadequate: No information reported | Adequate: Assumable as distribution of scores not presented for all data | Inadequate: No hypothesis reported, Reporting of experimental protocol led to difficulties evaluating methodology | Inadequate |
| Physical measure: Maximum walking time | Prasad et al (2016)^26^ | Very good | Inadequate: No information reported | Adequate: Assumable as distribution of scores not presented for all data | Inadequate: No hypothesis reported, Reporting of experimental protocol led to difficulties evaluating methodology | Inadequate |
| Physical measure: Time to first symptoms | Prasad et al (2016)^26^ | Very good | Inadequate: No information reported | Adequate: Assumable as distribution of scores not presented for all data | Inadequate: No hypothesis reported, Reporting of experimental protocol led to difficulties evaluating methodology | Inadequate |
| Physical measure: Distance to first symptoms | Prasad et al (2016)^26^ | Very good | Inadequate: No information reported | Adequate: Assumable as distribution of scores not presented for all data | Inadequate: No hypothesis reported, Reporting of experimental protocol led to difficulties evaluating methodology | Inadequate |
| **Activity in a natural environment physical outcome measures** | | | | | | |
| **Physical outcome measure: Step count** | | | | | | |
| Physical measure: Steps per day | Kim et al (2019)^27^ | Very good | Inadequate: No information reported | Very good | Inadequate: No hypothesis reported | Inadequate |
|  | Stienen et al (2020)^28^ | Very good | Doubtful: Some information reported in any population | Very good | Inadequate: No hypothesis reported, Sample size <20 | Inadequate |
|  | Bienstock et al (2022)^29^ | Very good | Inadequate: No information reported | Very good | Doubtful: Participants not blinded to real-time activity data, Sample size <20 | Inadequate |
| **Physical outcome measure: Gait cycles** | | | | | | |
| Physical measure: Distance walked per day | Voglis et al (2022)^30^ | Very good | Doubtful: Some information reported for some measures | Doubtful: Statistical measure appropriate but group all time points together for correlation analyses | Inadequate: No hypothesis reported, Sample size <20 | Inadequate |
| **Physical outcome measure: Gait cycles** | | | | | | |
| Physical measure: Gait cycles per day | Schulte et al (2010)^31^ | Very good | Inadequate: No information reported | Very good | Inadequate: No hypothesis reported, Selective reporting of results led to difficulties evaluating construct validity | Inadequate |
| Physical measure: Gait cycles per hour | Schulte et al (2010)^31^ | Very good | Inadequate: No information reported | Very good | Inadequate: No hypothesis reported, Selective reporting of results led to difficulties evaluating construct validity | Inadequate |
| Physical measure: Gait intensities per day | Schulte et al (2010)^31^ | Very good | Inadequate: No information reported | Very good | Inadequate: No hypothesis reported, Selective reporting of results led to difficulties evaluating construct validity | Inadequate |

| **Criterion validity** | | | | | |
| --- | --- | --- | --- | --- | --- |
| **Physical outcome measure / physical measure** | **Study** | **For continuous scores: Were correlations, or the area under the receiver operating curve calculated?** | **For dichotomous scores: Were sensitivity and specificity determined?** | **Were there any other important flaws in the design or statistical methods of the study?** | **TOTAL (Lowest score)** |
| **Impairment-based physical outcome measures** | | | | | |
| **Physical outcome measure: Gait parameters** | | | | | |
| Physical measure: Two-test test | Fujita et al (2019)^7^ | Very good | Very good | Doubtful: No measurement property information about reference standard in study population | Doubtful |
| **Performance-based physical outcome measures** | | | | | |
| Not studied | | | | | |
| **Activity in a natural environment physical outcome measures** | | | | | |
| **Physical outcome measure: Step count** | | | | | |
| Physical measure: Steps detected at thigh | Gilmore et al (2020)^32^ | Very good | NA | Doubtful: No reporting of blinding of condition during step count analysis or decisions regarding which participants trialed 2 vs 3 monitors | Doubtful |
| Physical measure: Steps detected at wrist | Gilmore et al (2020)^32^ | Very good | NA | Doubtful: No reporting of blinding of condition during step count analysis or decisions regarding which participants trialed 2 vs 3 monitors | Doubtful |

| **Responsiveness - Construct approach hypothesis testing (comparison with other outcome measurement instruments and before/after intervention)** | | | | | | | |
| --- | --- | --- | --- | --- | --- | --- | --- |
| **Physical outcome measure / physical measure** | **Study** | **Comparison with other outcome measurement instruments** | | **Before/after intervention** | **Applicable to both comparison with other outcome measurement instruments and before/after intervention** | | |
|  |  | **Is it clear what the comparator instrument(s) measure(s)?** | **Were the measurement properties of the comparator instrument(s) sufficient?** | **Adequate description of the intervention given?** | **Was the statistical method appropriate for the hypotheses to be tested?** | **Were there any other important flaws in the design or statistical methods of the study?** | **TOTAL (Lowest score)** |
| **Impairment-based physical outcome measures** | | | | | | | |
| **Physical outcome measure: Active Range of Movement** | | | | | | | |
| Physical measure: Lumbar extension | Häkkinen et al (2005)^33^ | Very good | Doubtful: Some information reported in any population | NA | Very good | Inadequate: No hypothesis reported, No reporting of who collected data and if they knew of results of baseline assessment testing | Inadequate |
| Physical measure: Lumbar, trunk and hip flexion and extension | Mannion et al (2005)^2^ | Very good | Doubtful: Some information reported in study population | NA | Very good | Inadequate: No hypothesis reported, No reporting of who collected data and if they knew of results of baseline assessment testing | Inadequate |
| Physical measure: Schober test | Häkkinen et al (2005)^33^ | Very good | Doubtful: Some information reported in any population | NA | Very good | Inadequate: No hypothesis reported, No reporting of who collected data and if they knew of results of baseline assessment testing | Inadequate |
| **Physical outcome measure: Gait parameters** | | | | | | | |
| Physical measure: Asymmetry of double support | Loske et al (2018)^34^ | Very good | Inadequate: No information reported | NA | Very good | Inadequate: Incomplete hypothesis (not able to determine anticipated strength of correlations), No reporting of who collected data and if they knew of results of baseline assessment testing | Inadequate |
| Physical measure: Stride length | Loske et al (2018)^34^ | Very good | Inadequate: No information reported | NA | Very good | Inadequate: Incomplete hypothesis (not able to determine anticipated strength of correlations), No reporting of who collected data and if they knew of results of baseline assessment testing | Inadequate |
| **Performance-based physical outcome measures** | | | | | | | |
| **Physical outcome measure: 1 min stair climb** | | | | | | | |
| Physical measure: Number of steps | Jakobsson et al (2019)^35^ | Very good | Very good | NA | Very good | Very good | Very good |
| **Physical outcome measure: 5-min walk test** | | | | | | | |
| Physical measure: Distance walked | Jakobsson et al (2019)^35^ | Very good | Very good | NA | Very good | Very good | Very good |
| **Physical outcome measure: 6-min walk test** | | | | | | | |
| Physical measure: Distance walked | Takenaka et al (2022)^36^ | NA | NA | Very good | Very good | Inadequate: No hypothesis reported for expected direction and magnitude of change scores, No reporting of who collected data and if they knew of results of baseline assessment testing | Inadequate |
|  | Takenaka et al (2023)^37^ | NA | NA | Very good | Very good | Inadequate: No hypothesis reported for expected direction and magnitude of change scores, No reporting of who collected data and if they knew of results of baseline assessment testing | Inadequate |
|  | Zeitlberger et al (2022)^15^ | NA | NA | Very good | Very good | Inadequate: No hypothesis reported for expected direction and magnitude of change scores, No reporting of who collected data and if they knew of results of baseline assessment testing | Inadequate |
|  | Maldaner et al (2021)^14^ | NA | NA | Very good | Very good | Inadequate: No hypothesis reported for expected direction and magnitude of change scores, No reporting of who collected data and if they knew of results of baseline assessment testing | Inadequate |
|  | Ziga et al (2023)^16^ | NA | NA | Very good | Very good | Inadequate: No hypothesis reported for expected direction and magnitude of change scores, No reporting of who collected data and if they knew of results of baseline assessment testing | Inadequate |
|  | Sosnova et al (2021)^38^ | Very good | Inadequate: No information reported | NA | Very good | Very good | Inadequate |
| Physical measure: Time to first symptoms | Zeitlberger et al (2022)^15^ | NA | NA | Very good | Very good | Inadequate: No hypothesis reported for expected direction and magnitude of change scores, No reporting of who collected data and if they knew of results of baseline assessment testing | Inadequate |
| Physical measure: Distance to first symptom | Zeitlberger et al (2022)^15^ | NA | NA | Very good | Very good | Inadequate: No hypothesis reported for expected direction and magnitude of change scores, No reporting of who collected data and if they knew of results of baseline assessment testing | Inadequate |
| **Physical outcome measure: 10-meter walk test** | | | | | | | |
| Physical measure: Walking speed | Wada et al (2022)^17^ | Very good | Very good | NA | Very good | Inadequate: No hypothesis reported for expected direction and magnitude of change scores, No reporting of who collected data and if they knew of results of baseline assessment testing | Inadequate |
| **Physical outcome measure: 50-foot walk test** | | | | | | | |
| Physical measure: Time to complete | Jakobsson et al (2019)^35^ | Very good | Very good | NA | Very good | Very good | Very good |
| **Physical outcome measure: Self-paced walking test** | | | | | | | |
| Physical measure: Distance walked | Tomkins-Lane et al (2020)^39^ | Very good | Inadequate: No information reported | NA | Very good | Inadequate: Incomplete hypothesis (not able to determine anticipated strength of correlations), No reporting of who collected data and if they knew of results of baseline assessment testing | Inadequate |
| Physical measure: Time walked | Tomkins-Lane et al (2020)^39^ | Very good | Inadequate: No information reported | NA | Very good | Inadequate: Incomplete hypothesis (not able to determine anticipated strength of correlations), No reporting of who collected data and if they knew of results of baseline assessment testing | Inadequate |
| **Physical outcome measure: Timed Up and Go** | | | | | | | |
| Physical measure: Time to complete | Maldaner et al (2021)^14^ | NA | NA | very good | very good | Inadequate: No hypothesis reported for expected direction and magnitude of change scores, No reporting of who collected data and if they knew of results of baseline assessment testing | Inadequate |
|  | Jakobsson et al (2019)^35^ | Very good | Very good | NA | Very good | Very good | Very good |
|  | Gautschi (2016)^40^ | Very good | Very good | NA | Inadequate: Calculated Pearson correlation coefficients at each time point and interpreted r values at different time points as an indication of responsiveness | Inadequate: Incomplete hypothesis (not able to determine anticipated direction and strength of correlations), No reporting of standardized data collection procedures, who collected data and if they knew of results of baseline assessment testing | Inadequate |
| **Physical outcome measure: Trunk muscle endurance (tests of repetition)** | | | | | | | |
| Physical measure: Repetitive arch-ups until exhaustion | Häkkinen et al (2005)^33^ | Very good | Doubtful: Some information reported in any population | NA | Very good | Inadequate: No hypothesis reported, No reporting of who collected data and if they knew of results of baseline assessment testing | Inadequate |
| Physical measure: Repetitive sit-ups until exhaustion | Häkkinen et al (2005)^33^ | Very good | Doubtful: Some information reported in any population | NA | Very good | Inadequate: No hypothesis reported, No reporting of who collected data and if they knew of results of baseline assessment testing | Inadequate |
| **Activity in a natural environment physical outcome measures** | | | | | | | |
| **Physical outcome measure: Step count** | | | | | | | |
| Physical measure: Steps per day | Mobbs et al (2016)^41^ | Very good | Inadequate: No information reported | NA | Very good | Inadequate: No hypothesis reported | Inadequate |
| **Physical outcome measure: Gait Posture Index** | | | | | | | |
| Physical measure: Gait Posture Index | Ghent et al (2020)^42^ | Very good | Inadequate: No information reported | NA | Very good | Inadequate: No hypothesis reported | Inadequate |
|  | Mobbs et al (2019)^43^ | Very good | Inadequate: No information reported | NA | Very good | Inadequate: No hypothesis reported, No reporting of blinding of researcher collecting data, Sample size <20 | Inadequate |
| **Physical outcome measure: Distance per day** | | | | | | | |
| Physical measure: Distance per day | Mobbs et al (2016)^41^ | Very good | Inadequate: No information reported | NA | Very good | Inadequate: No hypothesis reported | Inadequate |

| **Responsiveness - Criterion approach** | | | | | |
| --- | --- | --- | --- | --- | --- |
| **Physical outcome measure / physical measure** | **Study** | **For continuous scores: Were correlations between change scores, or the area under the Receiver Operator Curve calculated?** | **For dichotomous scales: Were sensitivity and specificity determined?** | **Were there any other important flaws in the design or statistical methods of the study?** | **TOTAL (Lowest score)** |
| **Impairment-based physical outcome measures** | | | | | |
| Not studied | | | | |  |
| **Performance-based physical outcome measures** | | | | | |
| **Physical outcome measure: 6-min walk test** | | | | | |
| Physical measure: Distance walked | Takenaka et al (2022)^36^ | Very good | NA | Doubtful: Some reporting of measurement property information. No reporting of standardized procedures for data collection for ODI, blinding of investigators for data collection or awareness of baseline scores | Doubtful |
|  | Takenaka et al (2023)^37^ | Very good | NA | Doubtful: Some reporting of measurement property information. No reporting of standardized procedures for data collection for ODI, blinding of investigators for data collection or awareness of baseline scores, though same profession collected data | Doubtful |
|  | Maldaner et al (2021)^14^ | Very good | NA | Doubtful: Some reporting of measurement property information for reference standard (but not for dichotomized subscale) and standardized data collection procedures | Doubtful |
|  | Zeitlberger et al (2022)^15^ | Very good | NA | Inadequate: No reporting of measurement properties for reference standard, standardized procedures for data collection, blinding of investigators for data collection or awareness of baseline scores | Inadequate |
| Physical measure: Time to first symptoms | Zeitlberger et al (2022)^15^ | Very good | NA | Inadequate: No reporting of measurement properties for reference standard, standardized procedures for data collection, blinding of investigators for data collection or awareness of baseline scores | Inadequate |
| Physical measure: Distance to first symptom | Zeitlberger et al (2022)^15^ | Very good | NA | Inadequate: No reporting of measurement properties for reference standard, standardized procedures for data collection, blinding of investigators for data collection or awareness of baseline scores | Inadequate |
| **Physical outcome measure: Timed Up and Go** | | | | | |
| Physical measure: Time to complete | Maldaner et al (2021)^14^ | Very good | NA | Doubtful: Some reporting of measurement property information for reference standard (but not for dichotomized subscale) and standardized data collection procedures | Doubtful |
| **Activity in a natural environment physical outcome measures** | | | | | |
| Not studied | | | | | |

| **Reliability** | | | | | | | | | |
| --- | --- | --- | --- | --- | --- | --- | --- | --- | --- |
| **Physical outcome measure / physical measure** | **Study** | **Patients stable between repeated measurements?** | **Time interval between repeated measurements appropriate?** | **Measurement conditions similar for the repeated measurements?** | **Professional(s) administer measurement without knowledge of scores or values of other repeated measurement(s)?** | **Professional(s) assign scores or determine values without knowledge of scores or values of other repeated measurement(s)?** | **Any other important flaws in the design or statistical methods of the study?** | **For continuous scores: Intraclass correlation coefficient (ICC)? For ordinal scores: Weighted kappa? For dichotomous/nominal scores: Kappa for each category against the other categories combined?** | **TOTAL (*Lowest score)*** |
| **Impairment-based physical outcome measures** | | | | | | | | | |
| **None studied** | | | | | | | | | |
| **Performance-based physical outcome measures** | | | | | | | | | |
| **Physical outcome measure: 5 repetitions sit to stand** | | | | | | | | | |
| Physical measure: Time to complete | Staartjes et al (2018)^12^ | Adequate: Assumable standard was met | Very good | Adequate: Assumable standard was met | Doubtful: Unclear, not reported | Doubtful: Unclear, not reported | Inadequate: Unclear when measurements were taken, who took them and which measurement properties surgical vs control group contributed to, for example no retest raw data for surgical group reported | Very good | Inadequate |
|  | Staartjes et al (2019)^9^, Inter-rater reliability of tele-supervised | NA: Inter-rater | NA: Inter-rater | NA: Inter-rater | Doubtful: Unclear, not reported | Doubtful: Unclear, not reported | Inadequate: No instructions about standardized test environment, procedures for participants to record test or procedures for independent timing of video recordings. | Adequate: ICC, model not described | Inadequate |
|  | Staartjes et al (2019)^9^, Reliability between measurement environments & tools | Adequate: Assumable standard was met | Very good | Doubtful: Unclear, not reported | Doubtful: Unclear, not reported | Doubtful: Unclear, not reported | Inadequate: Two factors vary in test-retest (environment and person timing), No instructions about standardizing test environment for data collection. | Doubtful: Pearson correlation coefficient without evidence for no systematic difference between measurements | Inadequate |
| **Physical outcome measure: 6 minute walk test** | | | | | | | | | |
| Physical measure: Distance | Maldaner et al (2020)^13^ | Adequate: Reasons to assume standard was met | Doubtful: Time interval not reported | Doubtful: Unclear, not reported | Doubtful: Unclear, not reported | Doubtful: Unclear, not reported | Very good | Adequate: ICC, model not described | Doubtful |
|  | Zeitlberger et al (2022)^15^ | Doubtful: Unclear, not reported | Very good | Doubtful: Unclear, not reported | Doubtful: Unclear, not reported | Doubtful: Unclear, not reported | Very good | Adequate: ICC, model not described | Doubtful |
| Physical measure: Time to first symptoms | Zeitlberger et al (2022)^15^ | Doubtful: Unclear, not reported | Very good | Doubtful: Unclear, not reported | Doubtful: Unclear, not reported | Doubtful: Unclear, not reported | Very good | Adequate: ICC, model not described | Doubtful |
| Physical measure: Distance to first symptoms | Zeitlberger et al (2022)^15^ | Doubtful: Unclear, not reported | Very good | Doubtful: Unclear, not reported | Doubtful: Unclear, not reported | Doubtful: Unclear, not reported | Very good | Adequate: ICC, model not described | Doubtful |
| **Physical outcome measure: Treadmill test** | | | | | | | | | |
| Physical measure: Time to first symptoms (pre-operative) | Deen et al (2000)^44^ | Adequate: Reasons to assume standard was met | Very good | Adequate: Reasons to assume standard was met | Doubtful: Unclear, not reported | Doubtful: Unclear, not reported | Inadequate: Several re-test timepoints and all combined for statistical analysis | Adequate: Concordance correlation coefficient with evidence of no systematic difference between measurements | Inadequate |
| Physical measure: Time to first symptoms (post-operative) | Deen et al (2000)^44^ | Adequate: Reasons to assume standard was met | Doubtful: Time interval not reported | Adequate: Reasons to assume standard was met | Doubtful: Unclear, not reported | Doubtful: Unclear, not reported | Inadequate: Post-operative design not described. 33% loss in sample size pre- to post-operative but no reason reported. | Adequate: Concordance correlation coefficient with evidence of no systematic difference between measurements | Inadequate |
| Physical measure: Total ambulation time  (pre-operative) | Deen et al (2000)^44^ | Adequate: Reasons to assume standard was met | Very good | Adequate: Reasons to assume standard was met | Doubtful: Unclear, not reported | Doubtful: Unclear, not reported | Inadequate: Several re-test timepoints and all combined for statistical analysis, 33% loss in sample size pre- to post-operative but no reason reported. | Adequate: Concordance correlation coefficient with evidence of no systematic difference between measurements | Inadequate |
| Physical measure: Total ambulation time  (post-operative) | Deen et al (2000)^44^ | Adequate: Reasons to assume standard was met | Doubtful: Time interval not reported | Adequate: Reasons to assume standard was met | Doubtful: Unclear, not reported | Doubtful: Unclear, not reported | Inadequate: Post-operative design not described. 33% loss in sample size pre- to post-operative but no reason reported. | Adequate: Concordance correlation coefficient with evidence of no systematic difference between measurements | Inadequate |
| **Activity in a natural environment physical outcome measures** | | | | | | | | | |
| **Not studied** | | | | | | | | | |

| **Measurement Error** | | | | | | | | | |
| --- | --- | --- | --- | --- | --- | --- | --- | --- | --- |
| **Physical outcome measure / physical measure** | **Study** | **Patients stable between repeated measurements?** | **Time interval between repeated measurements appropriate?** | **Measurement conditions similar for the repeated measurements?** | **Professional(s) administer the measurement without knowledge of scores or values of other repeated measurement(s)?** | **Professional(s) assign scores or determine values without knowledge of the scores or values of other repeated measurement(s)?** | **Any other important flaws in the design or statistical methods of the study?** | **For continuous scores: SEM, SDC, LoA or CV? For dichotomous/nominal/ordinal scores: Percentage specific agreement?** | **TOTAL (*Lowest score)*** |
| **Impairment-based physical outcome measures** | | | | | | | | | |
| **None studied** | | | | | | | | | |
| **Performance-based physical outcome measures** | | | | | | | | | |
| **Physical outcome measure: 5 repetitions sit to stand** | | | | | | | | | |
| Physical measure: Time to complete | Staartjes et al (2018)^12^ | Adequate: Assumable standard was met | Very good | Adequate: Assumable standard was met | Doubtful: Unclear, not reported | Doubtful: Unclear, not reported | Inadequate: Unclear when measurements were taken, who took them and which measurement properties surgical vs control group contributed to, for example no retest raw data for surgical group reported | Adequate: SEM, model not described | Inadequate |
|  | Staartjes et al (2019)^9^ | NA: Inter-rater | NA: Inter-rater | NA: Inter-rater | Doubtful: Unclear, not reported | Doubtful: Unclear, not reported | Inadequate: No instructions about standardized test environment, procedures for participants to record test or procedures for independent timing of video recordings. | Adequate: LOA, not fully described | Inadequate |
| **Physical outcome measure: 6 minute walk test** | | | | | | | | | |
| Physical measure: Distance | Maldaner et al (2020)^13^ | Adequate: Reasons to assume standard was met | Doubtful: Time interval not reported | Doubtful: Unclear, not reported | Doubtful: Unclear, not reported | Doubtful: Unclear, not reported | Very good | Adequate: SEM, model not described | Doubtful |
|  | Zeitlberger et al (2022)^15^ | Doubtful: Unclear, not reported | Very good | Doubtful: Unclear, not reported | Doubtful: Unclear, not reported | Doubtful: Unclear, not reported | Very good | Very good | Doubtful |
|  | Takenaka et al (2023)^37^ | Adequate: Reasons to assume standard was met | Very good | Very good | Doubtful: Unclear, not reported | Doubtful: Unclear, not reported | Very good | Very good | Doubtful |
| Physical measure: Time to first symptoms | Zeitlberger et al (2022)^15^ | Doubtful: Unclear, not reported | Very good | Doubtful: Unclear, not reported | Doubtful: Unclear, not reported | Doubtful: Unclear, not reported | Very good | Very good | Doubtful |
| Physical measure: Distance to first symptoms | Zeitlberger et al (2022)^15^ | Doubtful: Unclear, not reported | Very good | Doubtful: Unclear, not reported | Doubtful: Unclear, not reported | Doubtful: Unclear, not reported | Very good | Very good | Doubtful |
| **Activity in a natural environment physical outcome measures** | | | | | | | | | |
| **Physical outcome measure: Step count** | | | | | | | | | |
| Physical measure: Steps detected at thigh | Gilmore et al (2020)^32^ | Adequate: Reasons to assume standard was met | Doubtful: Unclear, not reported | Adequate: Reasons to assume standard was met | Doubtful: Unclear, not reported | Doubtful: Unclear, not reported | Very good | Very good | Doubtful |
| Physical measure: Steps detected at wrist | Gilmore et al (2020)^32^ | Adequate: Reasons to assume standard was met | Doubtful: Unclear, not reported | Adequate: Reasons to assume standard was met | Doubtful: Unclear, not reported | Doubtful: Unclear, not reported | Very good | Very good | Doubtful |

**S6 Appendix 2:** Summary of measurement properties

| **Physical outcome measure / physical measure** | **Authors (year)** | **Measurement time point** | **Physical measure result (mean (SD) unless otherwise noted)** | **Measurement property** | **Comparator** | **Statistical measure** | **Measurement property result** |
| --- | --- | --- | --- | --- | --- | --- | --- |
| **Impairment-based physical outcome measures** | | | | | | | |
| **Physical outcome measure: Active range of movement** | | | | | | | |
| Physical measure: Lumbar extension (Dualer goniometer) | Häkkinen et al (2005)^33^ | 2 months post-operative,  14 months post-operative | Med (IQR), degrees:  2 months post-operative: 10 (7-13)  14 months post-operative: 12 (10-18) | Responsiveness (Construct approach hypothesis testing, comparison with other outcome measures) | 15D | Pearson correlation coefficient | r=-0.02 (95% CI: -0.18-0.14) |
| Physical measure: Schober test |  | 2 months post-operative,  14 months post-operative | Med (IQR), cm:  2 month post-operative: 4 (3-5)  14 months post-operative: 5 (4-5) | Responsiveness (Construct approach hypothesis testing, comparison with other outcome measures) | 15D | Pearson correlation coefficient | r=0.16 (95% CI: -0.01-0.31) |
| Physical measure: Lumbar, trunk and hip flexion and extension (computer-assisted electronic inclinometer) | Mannion et al (2005)^2^ | 1-2 days pre-operative,  2 months post-operative | Pre-operative (degrees):  Hip flexion: 42.1 (18.9)  Trunk flexion: 84.7 (27.1)  Lumbar flexion: 42.8 (15.9)  Hip extension: 13.4 (8.9)  Trunk extension: 22.8 (10.3)  Lumbar extension: 6.9 (5.6)  2 months post-operative:  Hip flexion: 47.2 (16.6)  Trunk flexion: 85.9 (29.0)  Lumbar flexion: 36.7 (13.1)  Hip extension: 13.8 (8.6)  Trunk extension: 21.9 (11.3)  Lumbar extension: 6.7 (6.7) | Construct validity (Pre-operative comparison with other outcome measures) | RMDQ | Bivariate regression analysis | Pre-operative correlations only:  Lumbar flexion: r=0.61, p=0.0002  Trunk flexion: r=0.54, p=0.004  Hip flexion: r=0.43, p=0.01  Lumbar extension: r=0.57, p=0.0005  Trunk extension: r=0.61, p=0.0002  *Only reported statistically significant results |
|  |  |  |  | Responsiveness (Construct approach hypothesis testing, comparison with other outcome measures) | RMDQ | Bivariate regression analysis | Trunk flexion: r=-0.59, p=0.0004  Lumbar flexion: r=-0.82, p=0.0001  *Only reported statistically significant results |
| Physical measure: Lumbopelvic flexion and extension (Dual bubble inclinometer) | Pitino (2000)^3^ | Pre-operative PT,  First post-operative PT  Discharge from PT | Lumbar flexion (degrees):  Pre-operative: 9.86 (8.84), Range: 0-27  Post-operative: 14.19 (7.55), Range: 0-27  Discharge: 15.87 (7.24), Range 2.0-30.0  Lumbar extension (degrees):  Pre-operative: 12.6 (8.97), Range: 0-30  Post-operative: 12.31 (5.65), Range: 4-25  Discharge: 12.00 (4.36), Range: 5-19 | Construct validity (Comparison with other outcome measures) | North American Spine Society Questionnaire (Disability and neurogenic symptom subscales)  Straight leg raise | Spearman rho correlation coefficient | “Spearman rho correlations of the pooled data revealed a moderate relationship between involved SLR and lumbar flexion (p=0.424, p<.01).”  *Only reported statistically significant results |
| **Physical outcome measure: Handgrip strength** | | | | | | | |
| Physical measure: Handgrip maximum voluntary contraction (handheld dynamometer) | Inoue et al (2020)^6^ | Pre-operative | 30.2 (9.1) kg | Construct validity (Comparison with other outcome measures) | Area of skeletal muscle mass at L3  Area of psoas muscle at L3  Psoas muscle index  10m walk test time  10m walk test steps  Intermittent claudication (up to 300m walk distance)  Lower leg extension power | Pearson correlation coefficient | Area of skeletal muscle at L3: r=0.469  Area of psoas muscle at L3: r=0.380  Psoas muscle index: r=0.253  10m walk test time: r=-0.269  10m walk test steps: r=-0.352  Intermittent claudication: r=0.201 Lower leg extension power: r=0.723 |
|  | Kwon et al (2020)^4^ | Pre-operative, One year post-operative | 17.3 kg (unclear if pre- or post-operative measurement) | Construct validity (Comparison with other outcome measures) | History of falling  6 min walk test  Timed Up and Go  Sit to stand test  Radiological parameters (SVA, LL, PT, SS, PI, GT, T1PA, PI-LL, TK, TLK) | Pearson correlation coefficient | History of falling  Pre-operative: r=−0.135, p=0.202  Post-operative: r=−0.019, p=0.859  6 min walk test  Pre-operative: r=-0.196, p=0.063  Post-operative: r=-0.377, p<0.001  Timed Up and Go  Pre-operative: r=-0.156, p=0.139  Post-operative: r=-0.368, p<0.001  Sit to stand test  Pre-operative: r=-0.038, p=0.723  Post-operative: r=-0.164, p=0.120  SVA  Pre-operative: r=−0.375, p<0.001  Post-operative: r=−0.394, p<0.001  LL  Pre-operative: r=0.090, p=0.395  Post-operative: r=0.226, p=0.031  PT  Pre-operative: r=−0.277, p=0.008  Post-operative: r=−0.330, p=0.001  SS  Pre-operative: r=−0.031, p=0.770  Post-operative: r=0.079, p=0.458  PI  Pre-operative: r=−0.223, p=0.033  Post-operative: r=−0.242, p=0.021  GT  Pre-operative: r=−0.360, p< 0.001  Post-operative: r=−0.431, p< 0.001  T1PA  Pre-operative: r=−0.345, p=0.001  Post-operative: r=−0.407, p<0.001  PI-LL  Pre-operative: r=−0.252, p=0.016  Post-operative: r=−0.391, p<0.001  TK  Pre-operative: r=0.279, p=0.007  Post-operative: r=0.222, p=0.035  TLK  Pre-operative: r=-0.170, p=0.107  Post-operative: r=-0.190, p=0.071 |
|  | Kwon et al (2020)^5^ | Pre-operative | Not reported | Construct validity (Comparison with other outcome measures) | ODI  EQ-5D  VAS LBP  VAS leg pain  Alternative step test  6 meter walk test  Timed Up and Go  Sit to stand test | Abstract states Pearson correlation coefficient. Methods states partial correlation adjusted for age, BMI and operation level | ODI  Women: r=-0.164, p=0.070  Men: r=-0.384, p=0.001  EQ-5D  Women: r=0.190, p=0.036  Men: r=0.373, p=0.001  VAS LBP  Women: r=-0.151, p=0.095  Men: r=-0.056, p=0.646  VAS leg pain  Women: r=-0.215, p=0.017  Men: r=0.232, p=0.052  Alternative step test  Women: r=-0.147, p=0.105  Men: r=-0.381, p=0.001  6 meter walk test  Women: r=-0.239, p=0.008  Men: r=-0.097, p=0.423  Timed Up and Go  Women: r=-0.080, p=0.378  Men: r=-0.199, p=0.097  Sit to stand test  Women: r=-0.119, p=0.191  Men: r=-0.271, p=0.022 |
| **Physical outcome measure: Gait parameters** | | | | | | | |
| Physical measure: Two-step test | Fujita et al (2019)^7^ | One day pre-operative | 1.04 (0.22) | Construct validity (Comparison with other outcome measures) | Timed Up and Go | Pearson correlation coefficient | r=0.65, p<0.001 |
|  |  |  |  | Criterion validity | Timed Up and Go | ROC AUC, Sensitivity, Specificity | AUC: 0.83 (95% CI: 0.78-0.89), Sensitivity: 0.78, Specificity: 0.79  *Cut-off point of Two-Step test score set at 0.93 |
| Physical measure: Asymmetry of double support (RehabGait system) | Loske et al (2018)^34^ | One day pre-operative, 10 weeks post-operative, 12 months post-operative | Pre-op: 14.3 (13.5), Min-Max: 0.2-55.4  10 weeks: 11.4 (10.2), Min-Max: 0.1-39.9  12 months: 15.2 (12.9), Min-Max: 1.9-62.1 | Responsiveness (Construct approach hypothesis testing, comparison with other outcome measures) | ODI | Stepwise linear regression | 10 weeks post-operative: r=0.622  *Did not report coefficient for Asymmetry of double support at 12 months |
| Physical measure: Stride length (RehabGait system) |  |  | Pre-operative: 1.18 (0.26), Min-Max: 0.60-1.78  10 weeks: 1.21 (0.20), Min-Max: 0.78-1.72  12 months: 1.20 (0.22), 0.78-1.82 | Responsiveness (Construct approach hypothesis testing, comparison with other outcome measures) | ODI | Stepwise linear regression | 12 months post-operative: r=0.642  *Did not enter model at 10 weeks post-operative |
| **Performance-based physical outcome measures** | | | | | | | |
| **Physical outcome measure: 1-min stair climb** | | | | | | | |
| Physical measure: Number of stairs | Jakobsson et al (2019)^35^ | 8-12 weeks pre-operative, 6 months post-operative | Pre-operative (# steps):  Completed 6-month physical tests: 105.3 (24.8)  Improved: 95.3 (26.9) Unchanged: 114.1 (20.7)  6 months:  Completed 6-month physical tests: NR  Improved: 125.0 (24.3)  Unchanged: 127.1 (22.3) | Responsiveness (Construct approach hypothesis testing, comparison with other outcome measures) | Hypothesis 1: Construct specific GPE,  Hypothesis 2: Generic GPE,  Hypothesis 3: 5-min walk, 50-foot walk, TUG  Hypothesis 4: ODI  Hypothesis 5: VAS back pain | Hypothesis 1-2: ROC AUC  Hypothesis 3-5: Spearman's rho correlation coefficient | Hypothesis 1:  AUC: 0.72 (95% CI: 0.59 - 0.85)  Hypothesis 2:  AUC: 0.70 (95% CI: 0.59 - 0.81)  Hypothesis 3:  5-min walk: ρ=0.815, p<0.01  50-foot walk: ρ=-0.755, p<0.01  TUG: ρ=-0.67, p<0.01  Hypothesis 4: ρ=−0.396, p<0.01  Hypothesis 5: ρ=− 0.342, p<0.01 |
| **Physical outcome measure: 5 repetitions sit to stand** | | | | | | | |
| Physical measure: Time to complete | Klukowska et al (2020)^11^ | Pre-operative | Not reported | Construct validity (comparison with other outcome measures) | RMDQ  ODI  VAS back pain  VAS leg pain  EQ-5D index  EQ-5D VAS | Univariate linear regression | RMDQ  Mild OFI: β=1.37 (95% CI: −0.06 - 2.79), p=0.061  Moderate OFI: β=5.79 (95% CI: 3.91 - 7.68), p<0.001  Severe OFI: β=6.76 (95% CI: 4.64 - 8.89), p<0.001  ODI  Mild OFI: β=2.57 (95% CI: −2.06 - 7.20), p=0.277  Moderate OFI: β=12.05 (95% CI: 5.92 - 18.18), p<0.001  Severe OFI: β=19.43 (95% CI: 12.51 - 26.33), p<0.001  VAS back pain  Mild OFI: β=1.39 (95% CI: 0.65 - 2.14), p<0.001  Moderate OFI: β=2.32 (95% CI: 1.33 - 3.32), p<0.001  Severe OFI: β=2.36 (95% CI: 1.24 - 3.48), p=0.001  VAS leg pain  Mild OFI: β=−0.09 (95% CI: −0.66 - 0.48), p=0.748  Moderate OFI: β=0.18 (95% CI: −0.57 - 0.94), p=0.637  Severe OFI: β=0.51 (95% CI: −0.33 - 1.37), p=0.238  EQ-5D index  Mild OFI: β=0.01 (95% CI: −0.07 - 0.09), p=0.791  Moderate OFI: β=−0.19 (95% CI: −0.30 - −0.08), p<0.001  Severe OFI: β=−0.30 (95% CI: −0.42 - −0.17), p<0.001  EQ-VAS  Mild OFI: β=−3.16 (95% CI: −8.37 - 2.05), p=0.236  Moderate OFI: β=−5.59 (95% CI: −12.49 - 1.31), p=0.114  Severe OFI: β=−12.69 (95% CI: −20.46 - −4.91), p=0.002 |
|  | Master et al (2020)^8^ | Pre-operative | 34.8 (22.5) sec | Construct validity (comparison with other outcome measures) | ODI  Brief Pain Inventory back pain  Brief Pain Inventory leg pain | Spearman Rho | ODI: ρ=0.32, p<0.01  Brief Pain Inventory back pain: ρ=0.05  Brief Pain Inventory leg pain: ρ=0.10 |
|  | Staartjes et al (2018)^12^ | Pre-operative | 13.32 (7.87) sec  Retest: Not reported for surgical cohort | Measurement error | n/a | SEM | 1.47 |
|  |  |  |  | Reliability (test retest) | n/a | ICC (models not reported) | Consistency & absolute agreement: 0.97 (95% CI: 0.94–0.98) |
|  |  |  |  | Construct validity (comparison with other outcome measures) | RMDQ  ODI  VAS back pain  VAS leg pain  EQ-5D Index  EQ-5D VAS | Pearson correlation | RMDQ: r=0.49 (95% CI: 0.36 – 0.60), p<0.001  ODI: r=0.44 (95% CI: 0.30 – 0.56), p<0.001  VAS back pain: r=0.31 (95% CI: 0.16 –0.45), p<0.001  VAS leg pain: r=0.10 (95% CI: Not reported), p=0.207  EQ-5D index: r=-0.41 (95% CI: -0.53 - 0.27), p<0.001  EQ-5D VAS: r=-0.14 (95% CI: Not reported), p=0.091 |
|  | Staartjes et al (2019)^9^ | Pre-operative | Clinic: 13.75 (6.84) sec  Unsupervised: 13.54 (7.11) sec  Tele-supervised: 13.25 (5.66) sec | Reliability (between measurement environments & tools: supervised in clinic and unsupervised at home; supervised in clinic and tele-supervised) | n/a | Pearson’s product–moment correlation | Clinic and unsupervised: r=0.94 (95% CI: 0.91–0.96), p<0.001  Clinic and tele-supervised: r=0.90 (95% CI: 0.83–0.94), p<0.001 |
|  |  |  |  | Reliability (inter-rater of tele-supervised) | n/a | ICC (Two-way random effects model set for absolute agreement) | ICC: 0.996 (95% CI: 0.993–0.998), p<0.001 |
|  |  |  |  | Measurement error | n/a | Bland–Altman analysis of interrater agreement of tele-supervised | Mean interrater bias: −0.15 sec, 95% limits of agreement: −0.81 - 0.51 sec |
|  |  |  |  | Construct validity (comparison with other outcome measures) | RMDQ  ODI  VAS back pain  VAS leg pain  EQ-5D Index  EQ-VAS | Correlations (*Did not report specific statistical test) | RMDQ: r=0.48 (95% CI: 0.31–0.63), p<0.001  ODI: r=0.40 (95% CI: 0.21–0.55), p<0.001  VAS back pain: r=0.25 (95% CI: 0.05–0.43), p=0.017  VAS leg pain: p=0.189 (*did not report r value)  EQ-5D index: r=−0.24 (95% CI: −0.42 − 0.04), p=0.021  EQ-VAS: r=−0.30 (95% CI: −0.47 - −0.10), p=0.004 |
|  | Staartjes et al (2020)^10^ | Pre-operative | 13.4 (6.4) sec | Construct validity (comparison with other outcome measures) | VAS back pain  VAS leg pain | Univariate linear regression | VAS back pain: β=5.36 (95% CI: 2.87 - 7.85)  VAS leg pain: β=1.59 (95% CI: −0.13 - 3.30) |
| **Physical outcome measure: 5-min walk test** | | | | | | | |
| Physical measure: Distance walked | Jakobsson et al (2019)^35^ | 8-12 weeks pre-operative, 6 months post-operative | Pre-operative (m):  Completed 6-month physical tests: 422.3 (82.4)  Improved: 403.2 (94.2)  Unchanged: 445.5 (73.3)  6 months:  Completed 6-month physical tests: NR  Improved: 484.9 (66.7)  Unchanged: 474.7 (93.0) | Responsiveness (Construct approach hypothesis testing, comparison with other outcome measures) | Hypothesis 1: Construct specific GPE,  Hypothesis 2: Generic GPE,  Hypothesis 3: 5-min walk, 50-foot walk, TUG  Hypothesis 4: ODI  Hypothesis 5: VAS back pain | Hypothesis 1-2: ROC AUC  Hypothesis 3-5: Spearman's rho correlation coefficient | Hypothesis 1:  AUC: 0.68 (95% CI: 0.54 - 0.82)  Hypothesis 2:  AUC: 0.70 (95% CI: 0.58 - 0.82)  Hypothesis 3:  1-min stair climb: ρ=0.815, p<0.01  50-foot walk: ρ=-0.755, p<0.01  TUG: ρ=-0.586, p<0.01  Hypothesis 4: ρ=-0.422, p<0.01  Hypothesis 5: ρ=−0.342, p<0.01 |
| **Physical outcome measure: 6-min walk test** | | | | | | | |
| Physical measure: Distance walked | Maldaner et al (2020)^13^ | Pre-operative | 370 (137) m | Measurement error | n/a | SEM | 58.3 m |
|  |  |  |  | Reliability (test retest) | n/a | ICC (model not reported) | β=0.82 (95% CI: 0.75–0.88), p<0.001 |
|  |  |  |  | Construct validity (comparison with other outcome measures) | COMI back  ZCQ Symptom severity  ZCQ PF  VAS back pain  VAS leg pain | Pearson correlation coefficient | COMI back: r=−0.31 (95% CI: −0.5 - −0.08)  ZCQ Symptom severity: r=−0.32 (95% CI: −0.52 - −0.09)  ZCQ PF: r=−0.33 (95% CI: −0.52 - −0.10)  VAS back pain: r=−0.42 (95% CI: −0.59 - −0.20)  VAS leg pain: r=−0.32 (95% CI: −0.51 - −0.09) |
|  | Maldaner et al (2021)^14^ | Pre-operative and 6 weeks post-operative | Pre-operative: 401 (129) m  6 weeks post-operative: 495 (129) m | Construct validity (comparison with other outcome measures) | Timed up and go  COMI back  VAS back pain  VAS leg pain  ZCQ PF  ZCQ symptom severity  ZCQ pain subscale  ZCQ neuroischemic symptoms subscale | Pearson correlation coefficient | TUG z-score: r=−0.66 (95%CI: −0.76 - −0.53)  ZCQ PF: r=−0.57 (95%CI: −0.69 - −0.42)  ZCQ symptom severity: r=−0.49 (95%CI: −0.63 - −0.33)  ZCQ pain: r=−0.50 (95%CI: −0.64 - −0.34)  ZCQ Neuroischemic symptoms: r=−0.30 (95%CI: −0.47 - −0.11)  COMI back: r=−0.51 (95%CI: −0.64 - −0.35)  VAS back pain: r=−0.52 (95%CI: −0.65 - −0.36)  VAS leg pain: r=−0.51 (95%CI: −0.64 - −0.34) |
|  |  |  |  | Responsiveness (Criterion approach) | ZCQ satisfaction | ROC AUC | AUC: 0.70 (95% CI: 0.51–0.89) |
|  |  |  |  | Responsiveness (Construct approach hypothesis testing, pre-post intervention) | n/a | Standardized response mean | 6-min walk distance: 0.86  6-min walk z-score: 0.83 |
|  | Sosnova et al (2021)^38^ | Pre-operative and 6 weeks post-operative | Not reported | Responsiveness (patient reported) | ZCQ  COMI | Patient preference survey question: “Which instrument do you consider best in detecting differences in your symptoms?”  Analysis: Friedman test with Bonferroni correction | Percentage (sample size) of responses:  6-min walk test app: 78% (n=38)  ZCQ: 16% (n=8), p<0.001  COMI: 6% (n=3), p<0.001 |
|  | Takenaka et al (2022)^36^ | Pre-operative and 6 months post-operative | Pre-operative: 341.6 (170.8) m  6 months post-operative: 482.7 (92.4) m | Responsiveness (Criterion approach) | ODI | ROC AUC | AUC: 0.70 (95% CI: 0.52–0.89) Sensitivity: 0.57, Specificity: 0.71 |
|  |  |  |  | Responsiveness (Construct approach hypothesis testing pre-post intervention) | n/a | Cohen's r (effect size) | Cohen's r effect size: 0.79 |
|  | Takenaka et al (2023)^37^ | Pre-operative and post-operative at 6 and 23 months | Pre-operative: 311.5 (154.4) m  6 months post-operative: 449.0 (86.5) m  12 months post-operative: 443.6 (97.1) m | Responsiveness (Construct approach hypothesis testing pre-post intervention) | n/a | Cohen’s r (effect size) | Pre-operative to 6 months: r=0.78  Pre-operative to 12 months: r=0.77 |
|  |  |  |  | Responsiveness (Criterion approach) | ODI | ROC AUC | 6 month: AUC 0.72 (95% CI: 0.63–0.82), SN: 0.51, SP: 0.74  12 month: AUC 0.78 (95% CI: 0.69–0.86), SN: 0.72, SP: 0.65  Low walking level and severe disability level pre-operatively - 6 months: AUC 0.98 (95% CI: 0.94–1.00)  Low walking level and severe disability level pre-operatively - 12 months: AUC 0.90 (95% CI: 0.68–1.00)  High walking level and low disability level pre-operatively - 6 months: AUC 0.60 (95% CI: 0.43–0.76)  High walking level and low disability level pre-operatively - 12 months: AUC 0.51 (95% CI: 0.34–0.67) |
|  |  |  |  | Measurement error | n/a | SEM (SD x √(1-r) where r is the test-retest reliability coefficient) | 34.5 |
|  | Zeitlberger et al (2022)^15^ | Pre-operative and 6 weeks post-operative | Pre-operative: 401 (129) m  6 weeks post-operative: 495 (129) m | Measurement error | n/a | SEM (SD x √(1-intra-rater ICC)) | 58 m |
|  |  |  |  | Reliability (test retest) | n/a | ICC (Model details not reported) | ICC: 0.82 (95% CI: 0.75-0.87) |
|  |  |  |  | Construct validity (comparison with other outcome measures) | VAS back pain  VAS leg pain  ZCQ Symptom severity  ZCQ PF  COMI Back | Pearson correlation coefficient | VAS back: r=−0.52 (95% CI: −0.65 - −0.36)  VAS leg: r=−0.51 (95% CI: −0.64 - −0.34)  ZCQ Symptom severity: r=−0.49 (95% CI: −0.63 - −0.33)  ZCQ PF: r=−0.57 (95% CI: −0.69 - −0.42)  COMI: r=−0.51 (95% CI: −0.64 - −0.35) |
|  |  |  |  | Responsiveness (criterion approach) | ZCQ satisfaction | ROC AUC | AUC: 0.70 (95% CI: 0.52–0.90) |
|  |  |  |  | Responsiveness (Construct approach hypothesis testing pre-post intervention) | n/a | Standardized response mean | 0.86 |
|  | Ziga et al (2023)^16^ | Pre-operative and post-operative at 6 weeks and 3 months | Pre-operative: 378 (137) m  6 weeks post-operative: 490 (126) m  3 months post-operative: 518 (112) m | Construct validity (comparison with other outcome measures) | COMI  ZCQ PF  ZCQ SS  VAS back pain  VAS leg pain | Pearson correlation coefficient | Pre-operative:  COMI: -0.31  ZCQ PF: -0.31  ZCQ SS: -0.34  VAS back: -0.35  VAS leg: -0.36  6 weeks post-operative:  COMI: -0.43  ZCQ PF: -0.67  ZCQ SS: -0.50  VAS back: -0.39  VAS leg: -0.33  3 months post-operative:  COMI: -0.59  ZCQ PF: -0.58  ZCQ SS: -0.47  VAS back: -0.46  VAS leg: -0.59 |
|  |  |  |  | Responsiveness (Construct approach hypothesis testing pre-post intervention) | n/a | Standardized response mean | Pre-operative to 6 weeks post-operative: 0.95  6 weeks post-operative to 3 months post-operative: 0.98 |
| Physical measure: Distance to first symptoms | Zeitlberger et al (2022)^15^ | Pre-operative and 6 weeks post-operative | Pre-operative: 227 (161) m  6 weeks post-operative: 431 (194) m | Measurement error | n/a | SEM (SD x √(1-intra-rater ICC)) | 85 m |
|  |  |  |  | Reliability (test retest) | n/a | ICC (Model details not reported) | ICC: 0.83 (95% CI: 0.77-0.88) |
|  |  |  |  | Construct validity (comparison with other outcome measures) | VAS back pain  VAS leg pain  ZCQ Symptom severity  ZCQ PF  COMI Back | Pearson correlation coefficient | VAS back: r=−0.48 (95% CI: −0.62 - −0.31)  VAS leg: r=−0.58 (95% CI: −0.69 - −0.43)  ZCQ Symptom severity: r=−0.52 (95% CI: −0.65 - −0.36)  ZCQ PF: r=−0.64 (95% CI: −0.74 - −0.51)  COMI: r=−0.59 (95% CI: −0.71 - −0.44) |
|  |  |  |  | Responsiveness (criterion approach) | ZCQ satisfaction | ROC AUC | AUC: 0.75 (95% CI: 0.53–0.98) |
|  |  |  |  | Responsiveness (Construct approach hypothesis testing pre-post intervention) | n/a | Standardized response mean | 0.94 |
| Physical measure: Time to first symptoms | Zeitlberger et al (2022)^15^ | Pre-operative and 6 weeks post-operative | Pre-operative: 192 (127) sec  Post-operative: 304 (102) sec | Measurement error | n/a | SEM (SD x √(1-intra-rater ICC)) | 59 sec |
|  |  |  |  | Reliability (test retest) | n/a | ICC (Model details not reported) | ICC: 0.79 (95% CI: 0.72-0.85) |
|  |  |  |  | Construct validity (comparison with other outcome measures) | VAS back pain  VAS leg pain  ZCQ Symptom severity  ZCQ PF  COMI Back | Pearson correlation coefficient | VAS back: r=−0.33 (95% CI: −0. 50 - −0. 14)  VAS leg: r=−0.43 (95% CI: −0.58 - −0.25)  ZCQ Symptom severity: r=−0.39 (95% CI: −0.54 - −0.20)  ZCQ PF: r=−0.51 (95% CI: −0.65 - −0.35)  COMI: r=−0.44 (95% CI: −0.59 - −0.26) |
|  |  |  |  | Responsiveness (criterion approach) | ZCQ satisfaction | ROC AUC | AUC: 0.59 (95% CI: 0.34–0.83) |
|  |  |  |  | Responsiveness (Construct approach hypothesis testing, pre-post intervention) | n/a | Standardized response mean | 0.84 |
| **Physical outcome measure: 10-meter walk test** | | | | | | | |
| Physical measure: Walking speed | Wada et al (2022)^17^ | Pre-operative (between admission and surgery) and post-operative at 3, 6 and 12 months | Pre-operative: 0.97 (0.27) m/s  12 months post-operative: 1.16 (0.26) m/s | Construct validity (comparison with other outcome measures) | Pain Catastrophizing Scale | Pearson correlation coefficient | Pre-operative: -0.20  3 months post-operative: -0.31  6 months post-operative: -0.33  12 months post-operative: -0.40 |
|  |  |  |  | Responsiveness (Construct approach hypothesis testing, comparison with other outcome measures) | Pain Catastrophizing Scale | Pearson correlation coefficient | Change from pre-operative to 12 months: -0.25 |
| **Physical outcome measure: 50-foot walk test** | | | | | | | |
| Physical measure: Time to complete | Jakobsson et al (2019)^35^ | 8-12 weeks pre-operative, 6 months post-operative | Pre-operative (sec):  Completed 6-month physical tests: 9.2 (2.8)  Improved: 9.9 (3.8)  Unchanged: 8.6 (2.4)  6 months:  Completed 6-month physical tests: NR  Improved: 7.7 (1.5)  Unchanged: 8.3 (2.4) | Responsiveness (Construct approach hypothesis testing, comparison with other outcome measures) | Hypothesis 1: Construct specific GPE,  Hypothesis 2: Generic GPE,  Hypothesis 3: 5-min walk, 50-foot walk, TUG  Hypothesis 4: ODI  Hypothesis 5: VAS back pain | Hypothesis 1-2: ROC AUC  Hypothesis 3-5: Spearman's rho correlation coefficient | Hypothesis 1:  AUC: 0.80 (95% CI: 0.67 - 0.93)  Hypothesis 2:  AUC: 0.76 (95% CI: 0.66 - 0.87)  Hypothesis 3:  1-min stair climb: ρ=-0.755, p<0.01  5-min walk: ρ=-0.755, p<0.01  TUG: ρ=0.665, p<0.01  Hypothesis 4: ρ=0.467, p<0.01  Hypothesis 5: ρ=0.368, p<0.01 |
| **Physical outcome measure: Modified Sorensen test** | | | | | | | |
| Physical measure: Time to exhaustion | Dedering et al (2006)^18^ | 2 weeks to 1 day pre-operative, and 4 weeks post-operative | Pre-operative (sec):  All: 175 (72)  Men: 201 (58)  Women: 117 (68)  4 weeks post-operative:  All: 187 (86)  Men: 216 (78)  Women: 121 (70) | Construct validity (comparison with other outcome measures) | Borg at min 1, min 2 and min 3 of Modified Sorensen test  EMG L1 slope  EMG L5 slope  Physical activity  RMDQ  ODI  Self-efficacy scale  SF-36 domains | Spearman’s correlation coefficients | Pre-operative:  Borg min 1: ρ=0.36; min 2: ρ=0.63; min 3: ρ=0.61  EMG L1 slope: ρ=0.02  EMG L5 slope: ρ=0.41  Physical activity: M: ρ=0.53; W: ρ=0.70  RMDQ: M: ρ=-0.54; W: ρ=-0.60  ODI: M: ρ=-0.34; W: ρ=-0.58  Self-efficacy scale: M: ρ=0.14; W: ρ=0.52  SF-36 PF: M: ρ=0.28; W: ρ=0.62  SF-36 RP: M: ρ=0.17; W: ρ=0.30  SF-36 BP: M: ρ=0.24; W: ρ=0.10  SF-36 GH: M: ρ=0.25; W: ρ=0.58  SF-36 Vit: M: ρ=0.42; W: ρ=0.19  SF-36 SF: M: ρ=0.15; W: ρ=0.61  SF-36 RE: M: ρ=0.28; W: ρ=0.09  SF-36 MH: M: ρ=0.46; W: ρ=-0.08  4 weeks post-operative:  Borg min 1: ρ=0.55; min 2: ρ=0.68; min 3: ρ=0.72  EMG L1 slope: ρ=0.35  EMG L5 slope: ρ=0.26  Physical activity: M: ρ=0.42; W: ρ=0.71  RMDQ: M: ρ=-0.36; W: ρ=-0.91  ODI: M: ρ=-0.38; W: ρ=-0.88  Self-efficacy scale: M: ρ=0.37; W: ρ=0.83  SF-36 PF: M: ρ=0.32; W: ρ=0.73  SF-36 RP: M: ρ=0.66; W: ρ=0.77  SF-36 BP: M: ρ=0.52; W: ρ=0.78  SF-36 GH: M: ρ=0.05; W: ρ=0.39  SF-36 Vit: M: ρ=0.28; W: ρ=0.53  SF-36 SF: M: ρ=0.18; W: ρ=0.62  SF-36 RE: M: ρ=0.35; W: ρ=0.59  SF-36 MH: M: ρ=0.05; W: ρ=0.39 |
|  | Dedering (2012)^19^ | 2 years post-operative | All: 231 (86) sec  Men: 245 (84) sec  Women: 197 (87) sec | Construct validity (comparison with other outcome measures) | EMG L5 slope  Physical activity  RMDQ  ODI  Self-efficacy scale  SF-36 domains  Back Beliefs Questionnaire | Spearman’s correlation coefficient | EMG L5 Slope: M: ρ=0.17; W: ρ=0.96  Physical activity: M: ρ=0.48; W: ρ=0.95  RMDQ: M: ρ=-0.69; W: ρ=-0.90  ODI: M: ρ=-0.84; W: ρ=-0.94  Self-efficacy scale: M: ρ=0.82; W: ρ=0.93  SF-36 PF: M: ρ=0.77; W: ρ=0.86  SF-36 RP: M: ρ=0.48; W: ρ=0.42  SF-36 BP: M: ρ=0.71; W: ρ=0.83  SF-36 GH: M: ρ=0.46; W: ρ=0.31  SF-36 Vit: M: ρ=0.59; W: ρ=0.72  SF-36 SF: M: ρ=0.58; W: ρ=0.61  SF-36 RE: M: ρ=0.16; W: ρ=0.44  SF-36 MH: M: ρ=0.37; W: ρ=0.70  Back Beliefs Questionnaire: M: ρ=0.69; W: ρ=0.05 |
| **Physical outcome measure: Self-paced walking test** | | | | | | | |
| Physical measure: Distance walked | Tomkins-Lane et al (2020)^39^ | 1 week pre-operative, 6 weeks post-operative | Median (IQR) (m)  Pre-operative: 361.5 (135.4 - 736.1)  Post-Operative: 1911.1 (431.1 - 2210.4) | Responsiveness (Construct approach hypothesis testing, comparison with other outcome measures) | VAS back pain  VAS leg pain  ODI  SF-36  SSSQ PF domain  SSSQ symptom severity domain | Spearman’s Rho correlations | VAS back pre-walk: ρ=-0.21  VAS back post-walk: ρ=-0.59  VAS leg pre-walk: ρ=-0.33  VAS leg post-walk: ρ=-0.60  ODI composite: ρ=0.35  ODI pain: ρ=-0.74  SF-36 Pain: ρ=0.26  SF-36 General: ρ=-0.10  SF-36 PF: ρ=-0.65  SSSQ PF: ρ= -0.60  SSSQ Symptom Severity: ρ=-0.71 |
| Physical measure: Time walked | Tomkins-Lane et al (2020)^39^ | 1 week pre-operative, 6 weeks post-operative | Median (IQR) (sec)  Pre-operative: 383.0 (200.5 - 699.0)  Post-operative: 1800.0 (506.0 - 1800.0) | Responsiveness (Construct approach hypothesis testing, comparison with other outcome measures) | VAS back pain  VAS leg pain  ODI  SF-36  SSSQ PF domain  SSSQ symptom severity domain | Spearman’s Rho correlations | VAS back pre-walk: ρ=-0.20  VAS back post-walk: ρ=-0.61  VAS leg pre-walk: ρ=-0.22  VAS leg post-walk: ρ=-0.59  ODI Composite: ρ=0.18  ODI Pain: ρ=-0.67  SF-36 Pain: ρ=0.12  SF-36 General: ρ=-0.07  SF-36 PF: ρ=-0.55  SSSQ PF: ρ=-0.50  SSSQ Symptoms: ρ=-0.60 |
| **Physical outcome measure: Timed up and go** | | | | | | | |
| Physical measure: Time to complete | Corniola et al (2016)^22^ | Pre-operative | Did not report | Construct validity (comparison with other outcome measures) | MRI Modic classification type,  MRI Pfirrmann classification grade | Univariate logistic regression | Modic: OR: 0.97 (95% CI: 0.59 - 1.59), p=0.916  Pfirrman: OR: 1.13 (95% CI: 0.67 - 1.89), p=0.637 |
|  | Gautschi et al (2016)^23^ | Pre-operative | 11.3 (5.8) sec | Construct validity (comparison with other outcome measures) | VAS back pain  VAS leg pain  RMDI  ODI  SF-12 PCS  SF-12 MCS  EQ-5D Index  EQ-5D VAS | Pearson correlation coefficient | VAS back pain: r=0.29  VAS leg pain: r=0.30  RMDI: r=0.43  ODI: r=0.36  SF12 PCS: r=-0.33  SF12 MCS: r=-0.27  EQ5D index: r=-0.41  EQ-5D VAS: r=-0.28 |
|  | Gautschi et al (2016)^40^ | Pre-operative, 3 days post-operative, and 6 weeks post-operative | Pre-operative (sec): 10.3 (6.3)  3 days post-operative: 9.5 (4.3)  6 weeks post-operative: 6.5 (2.8) | Responsiveness (Construct approach hypothesis testing, comparison with other outcome measures) | VAS back pain  VAS leg pain  ODI  RMDI  EQ-5D Index  SF-12 PCS  SF-12 MCS | Pearson correlation coefficient | VAS back pain  Pre-operative: r=0.199, p=0.020  3 days post-operative: r= 0.366, p<0.001  6 weeks post-operative: r=0.226, p=0.008  VAS leg pain  Pre-operative: r=0.187, p=0.029  3 days post-operative: r=0.136, p=0.113  6 weeks post-operative: r=0.252, p=0.003  ODI  Pre-operative: r=0.324, p<0.001  3 days post-operative: r=0.395, p<0.001  6 weeks post-operative: r=0.413, p<0.001  RMDI  Pre-operative: r=0.136, p=0.113  3 days post-operative: r=0.340, p<0.001  6 weeks post-operative: r=0.435, p<0.001  EQ-5D Index  Pre-operative: r=−0.224, p=0.009  3 days post-operative: r=−0.420, p=<0.001  6 weeks post-operative: r=−0.363, p<0.001  SF-12 PCS  Pre-operative: r=−0.091, p=0.293  3 days post-operative: r=−0.230, p=0.007  6 weeks post-operative: r=−0.330, p<0.001  SF-12 MCS  Pre-operative: r=−0.171, p=0.047  3 days post-operative: r=−0.205, p=0.017  6 weeks post-operative: r=−0.174, p=0.042 |
|  | Jakobsson et al (2019)^35^ | 8-12 weeks pre-operative, 6 months post-operative | Pre-operative (sec):  Completed 6-month physical tests: 7.8 (3.0)  Improved chair rise: 9.1 (4.4)  Unchanged chair rise: 7.1 (1.6)  Improved walking: 9.0 (SD NR)  Unchanged walking: 6.9 (1.6)  6 months:  Completed 6-month physical tests: Not reported  Improved chair rise: 5.7 (1.2)  Unchanged chair rise: 6.4 (1.9)  Improved walking: 5.8 (SD NR)  Unchanged walking: 6.4 (1.9) | Responsiveness (Construct approach hypothesis testing comparison with other outcome measures) | Hypothesis 1: Construct specific GPE,  Hypothesis 2: Generic GPE,  Hypothesis 3: 1-min stair climb, 5-min walk, 50-foot walk  Hypothesis 4: ODI  Hypothesis 5: VAS back pain | Hypothesis 1-2: ROC AUC,  Hypothesis 3-5: Spearman's rho correlation coefficient | Hypothesis 1:  GPE walking AUC: 0.74 (95% CI: 0.61 - 0.86)  GPE chair rise AUC: 0.79 (95% CI: 0.67 - 0.91)  Hypothesis 2:  GPE walking AUC: 0.72 (95% CI: 0.67 - 0.91)  GPE chair rise AUC: 0.72 (95% CI: 0.62 - 0.83  Hypothesis 3:  1-min stair climb: ρ=−0.670, p<0.01  5-min walk: ρ=-0.586, p<0.01  50-foot walk: ρ= 0.665, p<0.01  Hypothesis 4: ρ=0.413, p<0.01  Hypothesis 5: ρ=0.286, p<0.01 |
|  | Maldaner et al (2021)^14^ | Pre-operative and 6 weeks post-operative | Pre-operative: 10.44 (4.37) sec  6 weeks post-operative: 8.47 (3.38) sec | Construct validity (comparison with other outcome measures) | 6-min walk test distance  ZCQ PF subscale  ZCQ symptom severity subscale  ZCQ pain subscale  ZCQ neuroischemic symptoms subscale  COMI back  VAS back pain  VAS leg pain | Pearson correlation coefficient | Correlations between combined pre and post-operative scores  6-min walk test distance: r=−0.66 (95% CI: −0.76 - −0.53)  ZCQ PF: r=0.45 (95% CI: 0.27 - 0.60)  ZCQ symptom severity: r=0.40 (95% CI: 0.22 - 0.56)  ZCQ pain: r=0.38 (95% CI: 0.19 - 0.54)  ZCQ neuroischemic symptoms: r=0.27 (95% CI: 0.07 - 0.45)  COMI back: r=0.40 (95% CI: 0.22 - 0.56)  VAS back pain: r=0.35 (95% CI: 0.16 - 0.52)  VAS leg pain: r=0.36 (95% CI: 0.17 - 0.52) |
|  |  |  |  | Responsiveness (criterion approach) | ZCQ satisfaction | ROC AUC | AUC: 0.53 (95% CI: 0.30–0.77) |
|  |  |  |  | Responsiveness (Construct approach hypothesis testing, pre-post intervention) | n/a | Standardized response mean | TUG in sec: 0.67  TUG z-score: 0.56 |
|  | Master et al (2020)^8^ | Pre-operative | 15.5 (8.1) sec | Construct validity (comparison with other outcome measures) | ODI  Brief Pain Inventory back pain  Brief Pain Inventory leg pain | Spearman Rho | ODI: ρ=0.29  Brief Pain Inventory back pain: ρ=0.06  Brief Pain Inventory leg pain: ρ=0.06 |
|  | Staartjes et al (2022)^21^ | Pre-operative during outpatient consultations or upon admission for surgical treatment | TUG test time (sec):  CCI 0: 10.62 (5.94)  CCI 1: 15.15 (15.65)  CCI 2: 10.75 (4.58)  CCI 3: 12.22 (6.86)  CCI 4: 13.27 (6.39)  ASA 1: 11.25 (8.68)  ASA 2: 10.82 (5.22)  ASA 3: 15.53 (18.10) | Construct validity (comparison with other outcome measures) | Medical comorbidities (Charlson Comorbidity Index, CCI; American Society of Anesthesiology, ASA) | Univariate linear regression | CCI and Tug Test time (sec), regression coefficient (95% CI)  CCI 1: 4.54 (2.25 to 6.82)  CCI 2: 0.14 (-3.59 to 3.86)  CCI 3: 1.61 (-3.97 to 7.18)  CCI 4: 2.65 (-6.90 to 12.20)  CCI and TUG t-score, regression coefficient (95% CI)  CCI 1: 15.81 (-1.26 - 32.89)  CCI 2: -10.57 (-38.44 - 17.30)  CCI 3: -8.40 (-50.11 - 33.31)  CCI 4: -9.93 (-81.42 - 61.55)  ASA and Tug Test time (sec), regression coefficient (95% CI)  ASA 2: -0.43 (-2.74 - 1.88)  ASA 3: 4.28 (1.06 - 7.50)  ASA and TUG t-score, regression coefficient (95% CI)  ASA 2: -19.09 (-36.20 - -1.98)  ASA 3: 3.67 (-20.17 - 27.52) |
|  | Stienen et al (2017)^24^ | Pre-operative | TUG t-score:  No OFI: 109.1 (8.2)  Mild OFI: 130.8 (5.0)  Moderate OFI: 147.8 (9.0)  Severe OFI: 221.3 (158.0) | Construct validity (comparison between subgroups) | VAS back pain  VAS leg pain  RMDI  ODI  EQ-5D Index  SF-12 PCS  SF-12 MCS | Univariate linear regression | VAS back pain  Whole cohort: β=0.42 (95% CI: 0.16 - 0.68), p=0.002  Mild OFI: β=0.75 (95% CI: −0.09 - 1.59), p=0.082  Moderate OFI: β=1.28 (95% CI: 0.48 - 2.08), p=0.002  Severe OFI: β=0.89 (95% CI: −0.02 - 1.79), p=0.055)  VAS leg pain  Whole cohort: β=0.76 (95% CI: 0.50-1.01), p<0.001)  Mild OFI: β=1.18 (95% CI: 0.34–2.02), p=0.006  Moderate OFI: β=1.71 (95% CI: 0.90–2.51), p<0.001)  Severe OFI: β=2.06 (95% CI: 1.15–2.97), p<0.001  RMDI  Whole cohort: β=1.89 (95% CI: 1.43-2.36), p<0.001  Mild OFI: β=3.38 (95% CI: 1.87–4.88), p<0.001  Moderate OFI: β=4.06 (95% CI: 2.62–5.49), p<0.001  Severe OFI: β=5.29 (95% CI: 3.67–6.91), p<0.001  ODI  Whole cohort: β=5.57 (95% CI: 3.97-7.18), p<0.001  Mild OFI: β=6.86 (95% CI: 1.64–12.09), p=0.010  Moderate OFI: β=11.83 (95% CI: 6.85–16.82), p<0.001  Severe OFI: β=16.03 (95% CI: 10.40–21.66), p<0.001  EQ-5D Index  Whole cohort: β=-0.074 (95% CI:-0.094 - -0.054), p<0.001  Mild OFI: β=−0.121 (95% CI: −0.185 - −0.056), p<0.001  Moderate OFI: β=−0.115 (95% CI: −0.177 - −0.054), p<0.001  Severe OFI: β=−0.241 (95% CI: −0.310 - −0.171), p<0.001  SF-12 PCS  Whole cohort: β=-1.81 (95% CI: -2.57 - -1.06), p<0.001  Mild OFI: β=−2.12 (95% CI: −4.57 - −0.33), p=0.089  Moderate OFI: β=−3.32 (95% CI: −5.66 - −0.98), p=0.005  Severe OFI: β=−5.63 (95% CI: −8.27 - −2.99), p<0.001  SF-12 MCS  Whole cohort: β=2.28 (95% CI:-3.33 - -1.22), p<0.001  Mild OFI: β=−4.52 (95% CI: −7.94 - −1.10), p=0.010  Moderate OFI: β=−3.49 (95% CI: −6.75 - −0.24), p=0.036  Severe OFI: β=−7.32 (95% CI:−11.00 - −3.64), p<0.001 |
|  | Stienen et al (2021)^20^ | Pre-operative, usually day before surgery | 10.8 (4.4) sec; Range 4.8 - 26.7 | Construct validity (comparison with other outcome measures) | VAS back pain  VAS leg pain  ZCQ symptom severity subscale  ZCQ pain subscale  ZCQ neuroischemic subscale  ZCQ PF subscale  COMI back  COMI pain intensity subscale  COMI disability subscale | Pearson correlation coefficient | VAS back pain: r=0.37, p=0.002  VAS leg pain: r=0.37, p=0.002  ZCQ symptom severity: r=0.41, p<0.001  ZCQ pain: r=0.41, p<0.001  ZCQ neuroischemic symptoms: r=0.24, p=0.042  ZCQ PF: r=0.36, p=0.002  COMI back: r=0.50, p<0.001  COMI pain intensity: r=0.37, p=0.002  COMI disability: r=0.26, p=0.032 |
| **Physical outcome measure: Treadmill test** | | | | | | | |
| Physical measure: Maximum walking distance | Herno et al (1999)^25^ | Post-operative 10.9 years (Range: 9.4-13.1 years) | No stenosis: 515 m  Stenosis: 470 m | Construct validity (comparison with other outcome measures) | MRI degenerative findings (Summative degenerative scale) and stenosis findings (Minimum area of dural sac) | Partial correlation coefficient | Degeneration: r=0.206,  Stenosis: r=0.016 |
|  | Prasad et al (2016)^26^ | Pre-operative and 6 months post-operative | Pre-operative: 230.21 (68.14) m  Post-operative: 446.88 (85.53) m | Construct validity (comparison with other outcome measures) | Thecal sac cross-sectional area  VAS leg pain  VAS back pain  ODI  JOA  SF-36 PCS  SF-36 MCS  Time to first symptoms | Pearson’s rank coefficient | Thecal sac cross-sectional area: r=0.35  VAS leg pain: r=−0.15  VAS back pain: r=−0.18  ODI: r=−0.16  JOA: r=0.13  SF‑36 PCS: r=−0.05  SF‑36 MCS: r=−0.26  (*Not reported if pre or post-operative correlations)  Pre-operative:  Time to first symptoms: r=0.62  VAS leg pain: r=0.3  ODI: r=-0.21  Post-operative:  ODI: r=-0.32 |
| Physical measure: Distance to first symptoms | Prasad et al (2016)^26^ | Pre-operative and 6 months post-operative | Pre-operative: 66.75 m (47.08)  Post-operative: 291.74 m (75.39) | Construct validity (comparison with other outcome measures) | Thecal sac cross-sectional area | Pearson’s rank coefficient | r=0.24 (*Not reported if pre or post-operative correlations) |
| Physical measure: Maximum walking time | Deen et al (2000)^44^ | Pre-operative test-retest on same day, within 1 day or within 2-4 days.  Post-operative test-retest timing not reported | Median (IQR) (min):  Pre-operative:  1.2 mph: 5.3 (2.0 - 9.3)  Preferred speed: 3.5 (1.7 - 8.5)  Post-operative:  1.2 mph: 15 (15 - 15)  Preferred speed: 5* (10.1 - 15)  *As reported | Reliability (test retest) | NA | Concordance correlation coefficient (CCC) | Pre-operative:  1.2 mph: CCC=0.89  Preferred speed: CCC=0.96  Post-operative:  CCC not reported. Stated “most patients completed a full 15-minute examination, and there was little variability” |
|  | Prasad et al (2016)^26^ | Pre-operative and 6 months post-operative | Pre-operative: 408.96 (121.68) sec  Post-operative: 802.32 (152.42) sec | Construct validity (comparison with other outcome measures) | Thecal sac cross-sectional area | Pearson’s rank coefficient | r=0.41 (*Not reported if pre or post-operative correlations) |
| Physical measure: Time to first symptoms | Deen et al (2000)^44^ | Pre-operative test-retest on same day, within 1 day or within 2-4 days.  Post-operative test-retest timing not reported | Median (IQR) (min):  Pre-operative:  1.2 mph: 0 (0 - 1.6)  Preferred speed: 0 (0 - 1.3)  Post-operative:  1.2 mph: 15 (10.8, 15)  Preferred sped: 15 (1.5, 15) | Reliability (test retest) | NA | Concordance correlation coefficient (CCC) | Pre-operative  1.2 mph: CCC=0.90  Preferred speed: CCC=0.98  Post-operative:  CCC not reported. Stated “most patients completed a full 15-minute examination, and there was little variability” |
|  | Prasad et al (2016)^26^ | Pre-operative and 6 months post-operative | Pre-operative: 117.67 sec (79.71)  Post-operative: 517 sec (153.3) | Construct validity (comparison with other outcome measures) | Thecal sac cross-sectional area | Pearson’s rank coefficient | r=0.21 (*Not reported if pre or post-operative correlations) |
| **Physical outcome measure: Trunk muscle endurance** | | | | | | | |
| Physical measure: Repetitive arch-ups until exhaustion | Häkkinen et al (2005)^33^ | 2 months post-operative,  14 months post-operative | Median (IQR) (repetitions):  2 months post-operative: 30 (12 - 41)  14 months post-operative: 37 (23 - 53) | Responsiveness (Construct approach hypothesis testing, comparison with other outcome measures) | 15D | Pearson’s correlation coefficient | r=0.16, (95% CI: -0.01 to 0.31) |
| Physical measure: Repetitive sit-ups until exhaustion | Häkkinen et al (2005)^33^ | 2 months post-operative,  14 months post-operative | Median (IQR) (repetitions):  2 months post-operative: 20 (2 - 31)  14 months post-operative: 27 (17 - 39) | Responsiveness (Construct approach hypothesis testing, comparison with other outcome measures) | 15D | Pearson’s correlation coefficient | r=0.13, (95% CI: -0.03 to 0.28) |
| **Activity in a natural environment physical outcome measures** | | | | | | | |
| **Physical outcome measure: Step count** | | | | | | | |
| Physical measure: Steps per day (using Fitbit Flex 2,^29^ Fitbit Charge,^27^ Fitbit Zip,^41^ Mi Band^28^) | Bienstock et al (2022)^29^ | Pre-operative (at least 1 week before surgery),  2 weeks post-operative,  1 month post-operative,  3 months post-operative,  6 months post operative | Pre-operative: 5536 (1314)  Not reported steps per day post-operatively | Construct validity (comparison with other outcome measures) | ODI  ODI Q4  SF-12 PCS | Spearman rank correlation coefficient | ODI  Pre-op: ρ=-0.22, p=0.35  2 wks: ρ=-0.49, p=0.04  1 month: ρ=-0.49, p=0.04  3 months: ρ=-0.57, p=0.02  6 months: ρ 0.10, p=0.73  ODI Q4  Pre-op: ρ=-0.18, p=0.45  2 wks: ρ=-0.26, p=0.32  1 month: ρ=-0.44, p=0.07  3 months: ρ=-0.58, p=0.02  6 months: ρ=-0.11, p=0.71  SF-12 PCS  Pre-op: ρ=0.34, p=0.14  2 wks: Analysis not completed - requires 4 week recall period  1 month: ρ=-0.56, p=0.02  3 months: ρ=0.77, p<0.001  6 months: ρ=0.67, p=0.008 |
|  | Kim et al (2019)^27^ | Day 1-7 post-operative | Post-operative day 1: 773 (1199.49)  Post-operative day 2: 1524.27 (2232.13)  Post-operative day 3: 2040.73 (1498.26)  Post-operative day 4: 3081.82 (1982.68)  Post-operative day 5: 3790.68 (2006.05)  Post-operative day 6: 4032.64 (2334.21)  Post-operative day 7: 5412 (2712.44) | Construct validity (comparison with other OMs) | VAS pain | Pearson correlation coefficient | r=-0.981, p<0.001 |
|  | Mobbs et al (2016)^41^ | 7 days pre-operative,  1 month post-operative,  2 months post-operative,  3 months post-operative | Pre-operative: 5255 (2883)  1 month: 4574 (2186)  2 months: 7135 (3112)  3 months: 8312 (4218) | Responsiveness (Construct approach hypothesis testing, comparison with other outcome measures) | VAS back pain  VAS leg pain  ODI  SF-12 MCS  SF-12 PCS | Pearson correlation coefficient | VAS back pain: r=0.446, p=0.316  VAS leg pain: r=0.472, p = 0.285  ODI: Did not report  SF-12 MCS: Did not report  SF-12 PCS: r = 0.058, p = 0.902  *Did not report which time point change scores were correlated |
|  | Stienen et al (2020)^28^ | Pre-operative,  3 months post-operative,  12 months post operative | Pre-operative: 4403 (1936)  3 months: 4679 (2265)  12 months: 3312 (2228) | Construct validity (comparison with other OMs) | ODI  PHQ-2 | Pearson correlation coefficient | ODI  Pre-operative: r=−0.164, p=0.612  3 months: r=−0.569, p=0.054  12 months: r=−0.075; p=0.926  PHQ-2  Pre-operative: r=−0.525, p=0.080  3 months: Analysis not completed. “PHQ-2 was 0 points for all patients with available step data, rendering analysis impossible.”  12 months: r=−0.762; p=0.238 |
| Physical measure: Steps detected at thigh (using ActivPAL3, Fitbit Flex, Jawbone UP Move) | Gilmore et al (2020)^32^ | Second or third day post-operative | ActivPAL3: 145 (54)  Fitbit Flex: 110 (82)  Jawbone: 36 (58) | Criterion validity | Observed number of steps from video recording | ICC, model 2.1 | ActivPAL3: 0.81 (95% 0.37-0.94)  Fitbit Flex: 0.11 (95% -0.15-0.44)  Jawbone: 0.71 (95% -0.02-0.91) |
|  |  |  |  | Measurement error | Observed number of steps from video recording | SEM: Standard deviation of difference between steps detected by activity monitor and observed step count | ActivPAL3: 23.2  Fitbit Flex: 35.8  Jawbone: 44.6 |
| Physical measure: Steps detected at wrist (using Fitbit Flex, Jawbone UP Move) | Gilmore et al (2020)^32^ | Second or third day post-operative | Fitbit Flex:  Wrist total: 46 (69)  No gait aid: 75 (84)  Gait aid: 17 (44)  Jawbone:  Wrist total: 38 (81)  No gait aid: 95 (107)  Gait aid: No steps detected | Criterion validity | Observed number of steps from video recording | ICC, model 2.1 | Fitbit Flex  Wrist total: 0.35 (95% -0.17-0.74)  No gait aid: 0.36 (95% -0.23-0.79)  Gait aid: 0.13 (95% -0.10-0.55)  Jawbone  Wrist total: 0.36 (95% -0.17-0.74)  No gait aid: 0.46 (95% -0.36-0.87)  Gait aid: no steps detected, unable to calculate |
|  |  |  |  | Measurement error | Observed number of steps from video recording | SEM: Standard deviation of difference between steps detected by activity monitor and observed step count | Fitbit Flex  Wrist total: 36.2  No gait aid: 43.3  Gait aid: 26.0  Jawbone  Wrist total: 40.5  No gait aid: 58.1  Gait aid: no steps detected, unable to calculate |
| **Physical outcome measure: Gait Posture Index** | | | | | | | |
| Physical measure: Gait Posture Index (using personal device e.g., Apple watch, Garmin or Mi Band 2^42,43^) | Ghent et al (2020)^42^ | 1 week of pre-operative,  3 months post-operative | Pre-operative: 59.33 (16.06), Range: 30-96  Post-operative: 85.75 (9.22), Range: 63-100 | Responsiveness (Construct approach hypothesis testing, comparison with other outcome measures) | ODI | Pearson correlation coefficient | r=0.56, p=0.005 |
|  | Mobbs et al (2019)^43^ | Pre-operative, 3 months post-operative | Pre-operative: 55.1754 (17.44204), Min-Max: 28.12-82.33  Post-operative: 75.9608 (11.10504), Min-Max: 52.91-87.91 | Responsiveness (Construct approach hypothesis testing, comparison with other outcome measures) | ODI  Patient satisfaction index as per Odom's criteria | Pearson correlation coefficient | ODI: r=0.682, p=0.01  Patient satisfaction: r=−0.618, p=0.024 |
| **Physical outcome measure: Distance per day** | | | | | | | |
| Physical measure: Distance per day (km / day using Fibit Zip^41^ and miles / day using Apple iOS Apple Health activity data from personal device^30^) | Mobbs et al (2016)^41^ | 7 days pre-operative,  1 month post-operative,  2 months post-operative,  3 months post-operative | Kilometers / day:  Pre-operative: 3.8 (2.2)  1 month: 3.4 (1.7)  2 month: 5.3 (2.5)  3 month: 6.2 (3.6) | Responsiveness (Construct approach hypothesis testing, comparison with other outcome measures) | VAS back pain  VAS leg pain  ODI  SF-12 MCS  SF-12 PCS | Pearson correlation coefficient | VAS back pain: r=0.333, p=0.348  VAS leg pain: r=-0.012, p=0.975  ODI: Did not report  SF-12 MCS: Did not report  SF-12 PCS: r=0.117, p=0.747  *Did not report which time point change scores were correlated |
|  | Voglis et al (2022)^30^ | Pre-operative and post-operative at 6 and 12 weeks | No raw descriptive data provided for walking distance, in figures only | Construct validity (comparison with other OMs) | 6WT app distance  COMI  ZCQ PF  ZCQ SS | Pearson correlation coefficient | 6WT app distance: r=0.57  COMI: r=0.62  ZCQ PF: r=0.68  ZCQ SS: r=0.52  *Pearson correlations at each time point not provided for 6WT in text (in figure only) and for PROMs only provided the overall correlations as appeared to group all timepoints together |
| **Physical outcome measure: Gait cycles** | | | | | | | |
| Physical measure: Gait cycles per day (using StepWatch 3) | Schulte et al (2010)^31^ | Pre-operative, 3 months post-operative,  12 months post-operative | Mean (SD provided only in figure):  Pre-operative: 3578  3 months post-operative: 4145  12 months post-operative: 4335 | Construct validity (comparison with other OMs) | VAS back pain  VAS leg pain  ODI  RMDQ  Radiological parameters (cross sectional area of spinal canal, thickness of yellow ligament, sagittal diameter of spinal canal, circumference of axial cross-section) | Spearman correlation coefficient | VAS back  Pre-operative: ρ=0.137  3 months: ρ=-0.142  12 months: ρ=-0.242  VAS leg  Pre-operative: ρ=-0.489,p<0.01  3 months: ρ=-0.227  12 months: ρ=-0.259  ODI  Pre-operative: ρ=-0.075  3 months: ρ=-0.284  12 months: ρ=-0.287  RMDQ  Pre-operative: ρ=-0.064  3 months: ρ=-0.134  12 months: ρ=-0.238  Radiological parameters: Did not report correlation coefficients. Stated, “Radiological data correlated neither with the StepWatch3 Activity Monitor results nor with the clinical data, particularly the VAS for leg pain.” |
| Physical measure: Gait cycles per hour (using StepWatch 3) | Schulte et al (2010)^31^ | Pre-operative, 3 months post-operative,  12 months post-operative | Pre-operative: 277 (127)  3 months: 323 (144)  12 months: 336 (137) | Construct validity (comparison with other OMs) | VAS back pain  VAS leg pain  ODI  RMDQ  Radiological parameters (cross sectional area of spinal canal, thickness of yellow ligament, sagittal diameter of spinal canal, circumference of axial cross-section) | Spearman correlation coefficient | VAS back  Pre-operative: ρ=0.041  3 months: ρ=-0.109  12 months: ρ=-0.266  VAS leg  Pre-operative: ρ=-0.426, p<0.001  3 months: ρ=-0.278  12 months: ρ=-0.341, p<0.05  ODI  Pre-operative: ρ=-0.046  3 months: ρ=-0.214  12 months: ρ=-0.278  RMDQ  Pre-operative: ρ=-0.089  3 months: ρ=-0.062  12 months: ρ=-0.219  Radiological parameters: Did not report correlation coefficients. Stated, “Radiological data correlated neither with the StepWatch3 Activity Monitor results nor with the clinical data, particularly the VAS for leg pain.” |
| Physical measure: Gait intensities per day (using StepWatch 3) | Schulte et al (2010)^31^ | Pre-operative, 3 months post-operative,  12 months post-operative | Pre-operative: 3.2 (3.8%)  3 months post-operative: 5.9 (6.4%)  12 months post-operative: 4.9 (3.9%) | Construct validity (comparison with other OMs) | VAS back pain  VAS leg pain  ODI  RMDQ  Radiological parameters (cross sectional area of spinal canal, thickness of yellow ligament, sagittal diameter of spinal canal, circumference of axial cross-section) | Spearman correlation coefficient | VAS back  Pre-operative: ρ=0.192  3 months: ρ=-0.088  12 months: - ρ=0.255  VAS leg  Pre-operative: ρ=0.267  3 months: ρ=-0.351, p<0.05  12 months: ρ=-0.229  ODI  Pre-operative: ρ=-0.121  3 months: ρ=-0.223  12 months: ρ=-0.289, p<0.05  RMDQ  Pre-operative: ρ=-0.125  3 months: ρ=-0.090  12 months: ρ=-0.250  Radiological parameters: Did not report correlation coefficients. Stated, “Radiological data correlated neither with the StepWatch3 Activity Monitor results nor with the clinical data, particularly the VAS for leg pain.” |

**S6 Appendix 3:** Hypotheses for validity and responsiveness (hypothesis testing approaches)

| **Study** | **Methods reported by study** | **Hypotheses or phrases reported indicating expected direction and strength of relationship** | **Derived hypotheses used in data synthesis** |
| --- | --- | --- | --- |
| Bienstock et al (2022)^29^ | “Correlations between VSMS and PROMs at each time point were measured using the Spearman rank correlation coefficient (r). Spearman r values of 0-0.39, 0.40-0.59, and 0.60-1 indicated weak, moderate, and strong correlations, respectively.” | "We hypothesized that accelerometry-measured physical activity levels would not correlate with traditional PROM data, thus signifying a novel measure quantifying patients’ recoveries" | Construct validity:  Unable to determine the authors hypothesized direction and strength of correlation between measures, and therefore unable to derive a hypothesis. Per COSMIN recommendations, p-values should not be used in testing hypotheses because it is not relevant to examine statistical difference from zero. |
| Corniola et al (2016)^22^ | “Using a uni- and multivariate logistic regression model, the effect size of the relationship between presence of TUG performance impairment and presence of severe MOD and PFI grades was estimated. “ | No hypothesis reported.  "We have the impression that the focus on imaging findings may be too high because the association of radiological signs of lumbar DDD and clinical symptoms is poor. Therefore, the aim of this study was to analyze the association between the MOD and PFI with established outcome measures of pain intensity, functional impairment, HRQoL, and functional mobility measured with the timed-up-and-go." | Construct validity:  Unable to determine the authors hypothesized direction and strength of correlation between measures, and therefore unable to derive a hypothesis. |
| Dedering et al (2006)^18^ | “Spearman’s correlation coefficients were used to describe correlation. Correlation coefficients (absolute values) ranging from 0.00 to 0.25 represented little if any correlation, 0.26–0.49 represented low correlation, 0.50–0.69 represented moderate correlation, 0.70–0.89 represented high correlation and 0.90–1.00 represented very high correlation.” | No hypothesis reported.  “The aim of the current study was to investigate back muscle fatigue and endurance in patients with lumbar disc herniation before and after surgery and to establish the degree of association between perceived fatigue and objectively measured fatigue. In addition, we investigated the relation between muscle fatigue and endurance versus activity, participation, self-efficacy and health to clarify the grade of association between these factors.” | Construct validity: Unable to determine the authors hypothesized direction and strength of correlation between measures, and therefore unable to derive a hypothesis. |
| Dedering (2012)^19^ | “Spearman’s correlation coefficient was used to correlate endurance time and EMG median frequency slope with the questionnaire scores.” | No hypothesis reported.  "The purpose was to explore outcome regarding possible changes in endurance time, EMG median frequency parameters and Borg ratings of back muscle fatigue and back pain." | Construct validity: Unable to determine the authors hypothesized direction and strength of correlation between measures, and therefore unable to derive a hypothesis. |
| Fujita et al (2019)^7^ | 1: “We calculated the sensitivity and specificity of the Two-Step test score for screening the high risk of falls in patients with LSS (TUGT score >=13.5 s) and determined its cut-off value as the score at which the Youden index (sensitivity + specificity - 1) was maximized. We further calculated the area under the curve (AUC) based on the receiver-operator curves.”  2: “Pearson's correlation coefficients were calculated to evaluate correlations between the score of the Two-Step test and that of TUGT.” | No hypothesis reported.  “Using these datasets, we first aimed to validate the Two-Step test as an indicator of functional mobility in patients with LSS. Second, we sought to clarify the factors associated with a short stride in elderly patients with LSS.” | 1: Responsiveness hypothesis derivation not required. AUC evaluated against COSMIN criteria for good measurement properties.  2: Responsiveness: Unable to determine the authors hypothesized direction and strength of correlation in change scores, and therefore unable to derive a hypothesis. |
| Gautschi et al (2016a)^23^ | "The relationship between TUG test times and various subjective outcome measures was measured using Pearson’s correlation coefficient (PCC)." | No hypothesis reported.  “We present the validation (and reliability measures) of the Timed Up and Go (TUG) test…We have evaluated a simple test, the Timed Up and Go (TUG) test, to aid clinical decision making by adding a more objective dimension to the assessment of functional disability in patients with lumbar DDD.” | Construct validity: Unable to determine the authors hypothesized direction and strength of correlation between measures, and therefore unable to derive a hypothesis. |
| Gautschi et al (2016b)^40^ | "The relationship between TUG test times and PROMs before, as well as at W6 postoperatively, was measured using Pearson correlation coefficient (PCC).” | “Validity is found to be present if the direction of the TUG t-scores changes correlates with the PROMs."  *The authors state validity is the measurement property under consideration. However, in accordance with COSMIN measurement property taxonomy, we interpret this to be responsiveness as change scores are of interest. | Responsiveness: Unable to determine the authors hypothesized direction and strength of correlation in change scores, and therefore unable to derive a hypothesis. |
| Ghent et al (2020)^42^ | “Pearson correlation analysis was performed to determine correlation between changes in the GPI and changes in the ODI.” | No hypothesis reported.  "Most studies examining the link between gait metrics and physical activity in spinal surgery have focused on lumbar canal stenosis. Our aim is to expand outcome assessment in LDH surgery to include gait metrics from wearable devices." | Responsiveness: Unable to determine the authors hypothesized direction and strength of correlation in change scores, and therefore unable to derive a hypothesis. |
| Gilmore et al (2020)^32^ | “The Intraclass Correlation Coefficient (ICC, model 2.1) was used to determine the association between the number of steps detected by each activity monitor, and the observed step count.” | “This study evaluated the concurrent validity of the ActivPAL3, Fitbit Flex, and Jawbone UP Move activity monitors when compared to observed step count, in patients immediately after lumbar  fusion surgery.”  *We interpret this to be a measure of criterion validity as observed step count is a reasonable gold standard. | Criterion validity: Hypothesis derivation not required. ICC evaluated against COSMIN criteria for good measurement properties. |
| Häkkinen et al (2005)^33^ | “Pearson’s correlation coefficient was used to determine the relationships between the variables.” | No hypothesis reported.  “This study compared health related quality of life (HRQOL) among lumbar disc surgery patients to general population, and assessed the relationships between HRQOL and other outcome measures 2 and 14 months after surgery.” | Construct validity: Unable to determine the authors hypothesized direction and strength of correlation between measures, and therefore unable to derive a hypothesis. |
| Herno et al (1999)^25^ | “Partial correlation coefficient was used to determine whether the degenerative or stenotic findings seen on MRI had stronger relation to the surgical outcome.” | “The current study began with the hypothesis that the surgical outcome is related more to postoperation stenotic findings than to degenerative findings as seen on MRI.” | Construct validity: Unable to determine the authors hypothesized strength of correlation between measures, and therefore unable to derive a hypothesis. |
| Inoue et al (2020)^6^ | “Correlation between the continuous variables was assessed using Pearson correlation coefficients.” | No hypothesis reported.  “The relationship among handgrip strength, LEP, psoas muscle, walking speed, and walking distance is still largely unknown in LSS patients. The purpose of the present study is to investigate the relationship among grip strength, leg extension power, walking speed, and intermittent claudication for LSS.” | Construct validity: Unable to determine the authors hypothesized direction and strength of correlation between measures, and therefore unable to derive a hypothesis. |
| Jakobsson et al (2019)^35^ | “Hypothesis 1 was tested by calculating the area under the receiver operating characteristic (ROC) curve for improved and unchanged patients, as classified by the construct-specific GPE scales matched for each particular physical capacity task…. Hypothesis 2 concerned the area under the ROC curve for improved and unchanged patients, as classified by the generic GPE scale… Hypotheses 3–5 were investigated with Spearman’s rho.” | Hypothesis 1. “The change scores (differences between baseline and 6-month assessments) of a physical capacity task will be able to distinguish between patients with and without meaningful improvement as classified by a construct-specific GPE scale (area under the ROC curve ≥0.70).”  Hypothesis 2. “The change scores of a physical capacity task will yield greater misclassifications of improved and unchanged patients on a ROC curve when that classification is based on a generic GPE scale rather than construct-specific GPE scales.”  Hypothesis 3. “The change scores of the four physical capacity tasks will be correlated ≥0.50 to each other in the expected direction.”  Hypothesis: 4. “The correlations between change scores of physical capacity tasks and the ODI will be at least 0.10 weaker than the correlations between the change scores among the physical capacity tasks themselves.”  Hypothesis 5. “The correlations between change scores of a physical capacity task and VAS on back pain intensity will be at least 0.10 weaker than the correlations between change scores of the physical capacity task and the ODI.” | Responsiveness: Hypotheses as stated by authors |
| Kim et al (2019)^27^ | “The relationship between postoperative daily VAS pain scores and postoperative physical activity (with focus on the number of steps taken daily from POD 1 to POD 7) was assessed by simple correlation analysis to determine whether a significant correlation existed between daily VAS scores and numbers of steps taken daily. The results of Pearson’s correlation analysis are presented as r and p values, where r values signify strengths of correlations (r values close to 1 indicate a strong positive correlation, while values closer to -1 indicate a strong negative correlation.” | No hypothesis reported.  “The present study was conducted to investigate the usefulness of wearable devices for the assessment of patients’ daily physical activities in a hospital environment and for exploring relationships between physical activity, postoperative pain and short-term surgical outcomes.” | Construct validity: Unable to determine the authors hypothesized direction and strength of correlation between measures, and therefore unable to derive a hypothesis. |
| Klukowska et al (2020)^11^ | “Linear regression models were fitted to assess the impact of the severity grading on included subjective PROMs with “no OFI” as the reference category.” | No hypothesis reported.  “The purpose of this study was to assess the concurrent validity of the 5R-STS BSS in patients with lumbar degenerative diseases by using data from two prospective studies.” | Construct validity: Unable to determine the authors hypothesized direction and strength of correlation between measures, and therefore unable to derive a hypothesis. |
| Kwon et al (2020a)^4^ | “Pearson’s correlations between HGS, radiologic parameters, and results of physical performance tests were evaluated.” | No hypothesis reported.  “This study aimed to examine the relationship between radiologic spinopelvic parameters (after the surgical treatment of patients with LSS) and skeletal muscle function, that is, the results of HGS and muscle performance tests.” | Construct validity: Unable to determine the authors hypothesized direction and strength of correlation between measures, and therefore unable to derive a hypothesis. |
| Kwon et al (2020b)^5^ | Abstract: “Correlations between HGS and clinical outcome parameters were analyzed using Pearson correlation.”  Methods: “Partial correlations adjusted for age, BMI, operation level between HGS and four functional mobility tests, and clinical outcomes were performed.” | No hypothesis reported.  “The aim of our present study was to investigate the prognostic value and the influence of HGS on surgical outcomes and risk of falls for LSS.” | Construct validity: Unable to determine the authors hypothesized direction and strength of correlation between measures, and therefore unable to derive a hypothesis. |
| Loske et al (2018)^34^ | “Stepwise linear regression models were used to  detect significant relationships between changes in ODI score and parameters describing gait function and quality.” | “We hypothesized that…changes in gait parameters will correlate with changes in ODI score.” | Responsiveness: Unable to determine the authors hypothesized direction and strength of correlation in change scores, and therefore unable to derive a hypothesis. |
| Maldaner et al (2020)^13^ | “Content validity of the 6WT was assessed as the relationship between the mean 6WD and the PROMs using a Pearson correlation.”  *The authors state content validity is the measurement property of interest. However, in accordance with COSMIN measurement property taxonomy, we interpret this to be construct validity (hypothesis testing, comparison with other outcome measures) as content validity aspects of relevance, comprehensiveness and comprehensibility were not investigated. | No hypothesis reported.  “The aim of this study, therefore, was to determine the reliability and validity of the smartphone app-based self-measured 6WT in patients with lumbar DDD.” | Construct validity: Unable to determine the authors hypothesized direction and strength of correlation between measures, and therefore unable to derive a hypothesis. |
| Maldaner et al (2021)^14^ | Construct validity: “Pearson correlation coefficients (r) were used to quantify the direction and strength of the relationship between pre- and postoperative 6WT results and objective (log-transformed TUG test results) as well as subjective outcome measures (i.e., PROMs).”  Responsiveness: “The internal responsiveness of  outcome measures was given by their corresponding standardized effect size. The external responsiveness of both objective measures of function was evaluated by receiver operating characteristic (ROC) curves using the ZCQ patient satisfaction subscale as a binary reference standard (combined score = 2 indicated a successful treatment).” | No hypothesis reported for construct validity and internal responsiveness.  “It is not known whether different measures of functional impairment correlate with each other…The purpose of this study was therefore to correlate 6WT and TUG test results in a cohort of patients undergoing surgery for DLDs and to evaluate the internal and external responsiveness of both instruments.” | Construct validity: Unable to determine the authors hypothesized direction and strength of correlation between measures, and therefore unable to derive a hypothesis.  Internal responsiveness: Unable to determine the authors hypothesized magnitude of expected effect size, and therefore unable to derive a hypothesis.  External responsiveness: Hypothesis derivation not required. AUC evaluated against COSMIN criteria for good measurement properties. |
| Mannion et al (2005)^2^ | “Relationships between changes in continuous variables were analysed with regression analysis (simple, and then stepwise multiple regression).” | No hypothesis reported.  Construct validity: “The correlation between spinal ROM and self-rated disability (Roland-Morris scores) was examined.”  Responsiveness: “Then, in a prospective manner, the relationship between individual changes in these objective (ROM) and subjective (Roland-Morris scores) measures, pre-surgery to post-surgery, was determined.” | Construct validity: Unable to determine the authors hypothesized direction and strength of correlation between measures, and therefore unable to derive a hypothesis.  Responsiveness: Unable to determine the authors hypothesized direction and strength of correlation in change scores, and therefore unable to derive a hypothesis. |
| Master et al (2020)^8^ | “Spearman Rho (r) was used to assess correlations between physical performance tests and PROMs of disability and pain at preoperative. Correlations were defined as weak if the coefficient was <0.3, moderate if it was between 0.3 and 0.5, and strong if it was >0.5.” | “We hypothesized that preoperative tests of strength and mobility, 5-Chair Stand and Timed Up and Go (TUG), would be significantly associated with disability and pain. An additional objective was to examine the relationship between physical performance tests and PROMs of disability and pain at the preoperative and 12-month follow-up timepoints.” | Construct validity: Unable to determine the authors hypothesized direction and strength of correlation between measures, and therefore unable to derive a hypothesis. Per COSMIN recommendations, p-values should not be used in testing hypotheses because it is not relevant to examine statistical difference from zero. |
| Mobbs et al (2016)^41^ | “The Pearson correlation test was performed to determine whether there was a significant correlation between changes in physical activity parameters (steps, distance) versus changes in clinical outcome (VAS, ODI, and SF-12 MCS and PCS scores).” | No hypothesis reported.  “No study thus far has prospectively investigated objective physical activity measurements after lumbar spine surgery and tested whether these measurements correlate well with subjective functional scores. Therefore, the aim of this study was to objectively measure functional outcome in patients who had lumbar spine surgery using quantitative physical activity measurements as derived from the accelerometers.” | Responsiveness: Unable to determine the authors hypothesized direction and strength of correlation in change scores, and therefore unable to derive a hypothesis. |
| Mobbs et al (2019)^43^ | “Pearson correlation analysis was performed to determine significant correlation between changes in physical activity parameters (step count, gait velocity and step length) versus change in PROMs (ODI and PSI/Odom’s score).” | No hypothesis reported.  “The aim of this prospective clinical study is to evaluate gait metrics including: (I) daily step count; (II) gait velocity; (III) step length; and (IV) body posture during ambulation including sagittal and coronal alignment for patients with LSS, and develop a simple and objective score of gait assessment: the Gait Posture index (GPi)…To test the reliability of the proposed GPi, a prospective, non-randomised single surgeon series of 13 patients with LSS was collected. Preoperative and postoperative data for 3 months were collected including GPi metrics (daily step count, gait velocity, mean step length and postural score), ODI and Patient Satisfaction Index (PSI) [Modified Odom’s criteria whereby 1: excellent, 2: good, 3: fair and 4: same or worse].”  *The authors state reliability is the measurement property under consideration. However, in accordance with COSMIN measurement property taxonomy, we interpret this to be responsiveness as correlations between changes in measurements are of interest. | Responsiveness: Unable to determine the authors hypothesized direction and strength of correlation in change scores, and therefore unable to derive a hypothesis. |
| Pitino et al (2000)^3^ | “Spearman rho correlation coefficients were performed on individual impairment measures with the LBP/disability subscale and neurogenic symptoms subscale of the NASS questionnaire.” | “The hypotheses were that there would be....a positive significant correlation between impairment and disability measures prior to and following surgical and physical therapy intervention.” | Construct validity: Unable to determine the authors hypothesized strength of correlation between measures, and therefore unable to derive a hypothesis. Per COSMIN recommendations, p-values should not be used in testing hypotheses because it is not relevant to examine statistical difference from zero. |
| Prasad et al (2016)^26^ | “Pearson’s rank coefficient was calculated between variables to assess the correlation.” | No hypothesis reported.  “This study was undertaken to assess the efficacy of surgery in bringing about a functional, clinical, and radiological improvement in cases having a degenerative LCS. A study of the factors that were influencing the surgical outcome was also undertaken.” | Construct validity: Unable to determine the authors hypothesized direction and strength of correlation between measures, and therefore unable to derive a hypothesis. |
| Schulte et al (2010)^31^ | “In addition to descriptive analyses, the following tests were applied: Mann–Whitney U test, Wilcoxon test, Spearman correlation coefficient McNemar test, 1-sample t test and Kruskal–Wallis test.” | No hypothesis reported.  “The aim of the present prospective study was to independently and objectively quantify the patients’ functional activity for several consecutive days using a new technique in normal living conditions both before and after lumbar decompressive surgery. The data were correlated with the clinical results, as well as with the radiologically assessed amount of central LSS.” | Construct validity: Unable to determine the authors hypothesized direction and strength of correlation between measures, and therefore unable to derive a hypothesis. |
| Sosnova et al (2021)^38^ | “Patients ratings for suitability and convenience of different outcome measures were compared using Friedman test with Bonferroni correction.” | No hypothesis reported.  “The aim of this study was to determine the patient’s preference regarding the use of an objective, app-based self-assessed outcome measure and compare it to commonly used subjective, questionnaire-based PROMs in paper form before and after surgery.”  Responsiveness survey question: “Which instrument do you consider best in detecting differences in your symptoms?” | Responsiveness: Unable to determine the authors hypothesized patient-reported preference, and therefore unable to derive a hypothesis. COSMIN does not include recommendations to evaluate patient-reported responsiveness. |
| Staartjes et al (2018)^12^ | “Pearson correlation was used to assess the correlation between log10-transformed 5R-STS test times and validated  questionnaires.” | No hypothesis reported.  “We aim to add a simple and objective tool to the spine surgeon’s armamentarium by evaluating the 5R-STS test for lumbar degenerative pathologies. In a prospective study, we assess the test’s correlation with validated PROMs and propose an upper limit of normal (ULN) and a severity stratification.” | Construct validity: Unable to determine the authors hypothesized direction and strength of correlation between measures, and therefore unable to derive a hypothesis. |
| Staartjes et al (2019)^9^ | “We also quantified convergent validity by correlating PROMs with log10- transformed 5R-STS test times obtained during clinical visits.” | No hypothesis reported.  “A range of PROMs were additionally used…baseline sociodemographic data, as well as numeric rating scales (NRS) for back and leg pain severity, and validated Dutch versions of the Oswestry Disability Index (ODI), Roland–Morris Disability Questionnaire (RMDQ), and EuroQOL-5D-3L (EQ- 5D) to capture subjective functional impairment as well as HRQOL. Participants filled in the questionnaires right after initially performing the test during the clinical visit. We assessed convergent validity of the 5R-STS by correlation with these secondary outcome measures.” | Construct validity: Unable to determine the authors hypothesized direction and strength of correlation between measures, and therefore unable to derive a hypothesis. |
| Staartjes et al (2020)^10^ | “Linear and binomial logistic regression models were fitted to evaluate the influence of maximum pain severity on 5R-STS raw test times, as well as on the presence of OFI and the presence of severe OFI.” | No hypothesis reported.  “In an analysis of data from two prospective clinical  studies, we aim to quantify the association among maximum patient-reported back or leg pain and OFI as measured by the 5R-STS.” | Construct validity: Unable to determine the authors hypothesized direction and strength of correlation between measures, and therefore unable to derive a hypothesis. |
| Staartjes et al (2022)^21^ | “Linear regression models were fitted to evaluate the influence of the severity grading on TUG performance, with CCI = 0 or ASA =1 as the reference category.” | No hypothesis reported.  "The goal of this study consequently was to quantify the extent of association among medical comorbidities and OFI in a population of patients with degenerative lumbar spine disease." | Construct validity: Unable to determine the authors hypothesized direction and strength of correlation between measures, and therefore unable to derive a hypothesis. |
| Stienen et al (2017)^24^ | “Direct and an adjusted linear regression model were built to relate the independent to the dependent variables.” | No hypothesis reported.  “The purpose of this work was to validate the OFI BSS and demonstrate its relationship to commonly used subjective outcome measures…Validity refers to the ability of the OFI BSS to indicate which individuals have functional impairment and which do not.” | Construct validity: Unable to determine the authors hypothesized strength of correlation in measures between individuals with and without functional impairment. Therefore, unable to derive a hypothesis. |
| Stienen et al (2020)^28^ | “Pearson correlation coefficients were calculated between PROMs and activity levels.” | No hypothesis reported.  “This study aimed at determining the feasibility of a low-cost consumer grade accelerometer (LCA) for objective assessment of pre- and postoperative step activity.” | Construct validity: Unable to determine the authors hypothesized direction and strength of correlation between measures, and therefore unable to derive a hypothesis. |
| Stienen et al (2021)^20^ | “A log transformation was conducted to evaluate content validity of the TUG test with PROMs using Pearson correlation, interpreting values between 0 and 0.3 as a weak, 0.3 and 0.7 as a moderate, and between 0.7 and 1 as a strong relationship.”  *The authors state content validity is the measurement property of interest. However, in accordance with COSMIN measurement property taxonomy, we interpret this to be construct validity (hypothesis testing, comparison with other outcome measures) as content validity aspects of relevance, comprehensiveness and comprehensibility were not investigated. | "We hypothesized that pearson correlation coefficients (PCCs) between TUG test results and PROMs would again be weak to moderate.” | Construct validity: PCC between TUG test results and each PROM will be ≤0.70.  PROMs evaluated include: VAS back pain, VAS leg pain, ZCQ symptom severity, ZCQ pain, ZCQ neuroischemic symptoms, ZCQ physical function, COMI back, COMI pain intensity, COMI disability |
| Takenaka et al (2022)^36^ | “Cohen r was used to evaluate the magnitude of effect size, with r ≥ 0.1–0.3 considered to indicate a small effect size; r ≥ 0.3-0.5*, a medium effect size; and r ≥ 0.5, a large effect size.”  *r ≥ 0.3-0.5 we interpret to indicate r = 0.3-0.5, for example.  “The receiver operating characteristic (ROC) curve was used to estimate the sensitivity and specificity of changes in the 6MWD threshold. The probability that these scores would correctly differentiate between “responders” and non-responders” was depicted by the area under the receiver operating characteristic curve (AUC). This value ranged from 0.5 to 1.0. An AUC of 0.7–0.8 was considered adequate and that of 0.8–0.9 was considered excellent.” | No hypothesis reported regarding the expected magnitude of effect size.  “This study aimed to evaluate the responsiveness of the 6MWD and determine the threshold of the MCID in the 6MWD in patients who underwent LSS surgery.” | Responsiveness (construct approach): Unable to determine the authors hypothesized magnitude of expected effect size, and therefore unable to derive a hypothesis.  Responsiveness (criterion approach): Hypothesis derivation not required. AUC evaluated against COSMIN criteria for good measurement properties. |
| Takenaka et al (2023)^37^ | “Cohen r was used to evaluate the magnitude of the effect size; r ≥ 0.1 to 0.3, ≥ 0.3 to 0.5, and ≥ 0.5 was considered small, medium, and large effect sizes, respectively. Preoperative and postoperative scores were compared using these analyses.”  “A receiver-operating characteristic (ROC) curve was used to estimate the sensitivity and specificity of change in the 6MWD threshold. The probability that scores would correctly discriminate between responders and non-responders was depicted by the area under the ROC curve (AUC).” | No hypothesis reported regarding the expected magnitude of effect size.  “We used a sample size of at least 100 cases and a 12-month follow-up duration post-operatively and hypothesized that a larger sample size would allow the MCID calculation with better validity than that performed in previous efforts.” | Responsiveness (construct approach): Unable to determine the authors hypothesized magnitude of expected effect size, and therefore unable to derive a hypothesis.  Responsiveness (criterion approach): Hypothesis derivation not required. AUC evaluated against COSMIN criteria for good measurement properties. |
| Tomkins-Lane et al (2020)^39^ | “Correlations of change between the different pain and outcome measures were calculated using Spearman’s Rho.” | “Our hypothesis was that changes in pain measured following provocation (after a symptom-limited walking test) would be more highly related to other outcomes in LSS as compared to pain measured at rest or averaged pain over the past month. Therefore, the purpose of this pilot study was to examine the relationship between changes in pain, measured at a number of different time points, with changes in other common outcomes in LSS, including reported and measured markers." | Responsiveness: Unable to determine the authors hypothesized strength of correlation in change scores, and therefore unable to derive a hypothesis. |
| Voglis et al (2022)^30^ | “Pearson correlation coefficients (R) were calculated to quantify the direction and strength of the relationship between pre- and postoperative 6WT results and normalized daily distances for each patient.” | No hypothesis reported.  “The aim of this study was therefore to analyze the usability of retrospectively collected smartphone-based, real-life activity data for physical performance assessment and to compare it with a set of pre specified, prospectively collected subjective as well as objective outcome measures in a cohort of patients undergoing surgery for DLD." | Construct validity: Unable to determine the authors hypothesized direction and strength of correlation between measures, and therefore unable to derive a hypothesis. |
| Wada et al (2022)^17^ | “Pearson correlation coefficients were used to assess the association between PCS and each variable at each evaluation time point and the association between the change in PCS and the change in each variable from baseline to 12 months postoperatively.” | No hypothesis reported.  "In this study, we evaluated changes in pain catastrophizing and walking speed from the preoperative to postoperative periods in patients with LSS and aimed to investigate whether walking speed is related to postoperative changes in pain catastrophizing." | Construct validity: Unable to determine the authors hypothesized direction and strength of correlation between measures, and therefore unable to derive a hypothesis.  Responsiveness: Unable to determine the authors hypothesized strength of correlation in change scores, and therefore unable to derive a hypothesis. |
| Zeitlberger et al (2022)^15^ | Construct validity: “Pearson correlation coefficients (r) were used to define the relationship between pre- and postoperative 6WT results and subjective outcome measures (PROMs).”  *Abstract states assessed content validity, while results section states assessed convergent validity. In accordance with COSMIN measurement property taxonomy, we interpret this to be construct validity (hypothesis testing, comparison with other outcome measures) as content validity aspects of relevance, comprehensiveness and comprehensibility were not investigated.  “The internal responsiveness of the 6WT results was assessed using standardized effect size (standardized response mean (SRM) = mean score of change from baseline to follow-up, divided by the SD of the score change). In accordance with prior research, SRM values were deemed as small (> 0.20), moderate (> 0.50), or large (> 0.80).”  “The external responsiveness of 6WT results was evaluated using receiver operating characteristics (ROC) curves. A reference standard indicating successful versus unsuccessful treatment was created by grouping results of the ZCQ patient satisfaction subscale into a binary variable of satisfied versus dissatisfied... An area under the curve (AUC) of 0.5 indicates no discrimination (no better than chance), whereas an AUC of 1.0 indicates perfect discrimination” | Construct validity: No hypothesis reported for expected correlations between measures.  Internal responsiveness: No hypothesis reported for expected magnitude of effect size.  External responsiveness: “We hypothesize that the pre- to postoperative change in both measures may help to differentiate between treatment successes in patients with lumbar DLD and compare the responsiveness to the traditional 6WD outcome.” | Construct validity: Unable to determine the authors hypothesized direction and strength of correlation between measures, and therefore unable to derive a hypothesis.  Internal responsiveness: Unable to determine the authors hypothesized magnitude of expected effect size, and therefore unable to derive a hypothesis.  External responsiveness: Hypothesis derivation not required. AUC evaluated against COSMIN criteria for good measurement properties. |
| Ziga et al (2023)^16^ | Construct validity: “Pearson correlation coefficients were used to define the relationship between 6WT results and PROMs preoperatively as well as 6 weeks and at 3 months postoperatively.”  Responsiveness: “Internal responsiveness of the 6WT results as well as PROMs was calculated using standardized effect size (standardized response mean [SRM] = mean score of change from baseline to follow-up, divided by the SD of the score change). As stated in previous research, SRM values were deemed as small (>0.20), moderate (>0.50) ,or large (0.80).” | No hypothesis reported.  "This study assesses change in outcome measured with a set of commonly used PROMs 6-weeks as well as 3 monhs after surgery for DLD and compares results to changes in OFI as measured by the 6WT." | Construct validity: Unable to determine the authors hypothesized direction and strength of correlation between measures, and therefore unable to derive a hypothesis.  Responsiveness: Unable to determine the authors hypothesized magnitude of expected effect size, and therefore unable to derive a hypothesis. |

**S6 Appendix 4:** Elements of a comprehensive research question for reliability and measurement error of physical measures

| **Deen et al (2000)**^44^ | |
| --- | --- |
| **Elements** |  |
| **1. Type and name of instrument** | Performance-based physical outcome measure: Treadmill test |
| **2. Operationalization** | **Equipment:** Treadmill |
|  | **Personnel:** No details reported |
|  | **Test procedures:** Examinations conducted between 8am – 5pm. Patients instructed to walk with an upright posture and avoid using front or side handrails. Treadmill inclined at 0˚. Treadmill started at 1.2 mph and patients were instructed to tell the examiner when symptoms first appeared. This time was recorded as time to first symptoms. A time of zero was recorded when symptoms were present at the beginning of the test. The exam was stopped after 15 minutes or at the onset of severe symptoms, defined as the level of discomfort that would cause patients to stop walking in usual life situations. The test procedure was conducted again at the patient’s preferred walking speed. The two tests were conducted pre- and post-operatively. |
|  | **Variables reported:** Time to first symptoms and total ambulation time at 1.2 mph and preferred walking speed |
| **3. Construct** | Walking capacity |
| **4. Measurement property** | Reliability (test retest) |
| **5. Components that will be repeated** | The entire test procedure was repeated pre- and post-operatively |
| **6. Source of variation** | Time. Pre-operative: 9 patients retested on the same day, 16 within 1 day, and 3 within 2-4 days. Post-operative: Sample and timing of retest not reported |
| **7. Patient population** | Surgical procedure: Laminectomy for stenosis, n=28 pre-operative, n=18 post-operative, Age: mean: 73.8, range: 57 – 91, 39% women pre-operative, gender not reported post-operative. |
| **Comprehensive research question:** What is the test-retest reliability of time to first symptoms and total ambulation time conducted during a treadmill test at 1.2 mph and preferred walking speed pre- and post-operatively in patients undergoing laminectomy for stenosis? | |

| **Gilmore et al (2020)**^32^ | |
| --- | --- |
| **Elements** |  |
| **1. Type and name of instrument** | Activity in natural environment physical outcome measure: Step count |
| **2. Operationalization** | **Equipment:** ActivPAL3 accelerometer, Fitbit Flex and Jawbone UP Move |
|  | **Personnel:** All video recordings were reviewed by a single researcher, no further details reported |
|  | **Test procedures:** Testing took place on the second or third post-operative day. Each participant tested either two or three activity monitors: all 40 participants wore one Fitbit and one Jawbone on the wrist or thigh (20 trials per monitor per position), and 20 participants wore an ActivPAL3. Participants were instructed to walk around the hospital ward at a comfortable pace for 2 minutes, using their prescribed walking aid if required. ActivPAL3 data were downloaded using software provided by PAL Technologies. The number of steps detected by the ActivPAL3 device during the timed walk test was obtained from the downloaded data. On completing the timed walk the Fitbit and Jawbone were re-synced with the smartphone application, the step count over the 2-minute time period was recorded from the smartphone application. The timed walk was recorded and the number of steps taken over the 2 minutes was counted. One “step” was defined as lifting one foot and returning it to the floor. The number of steps on the video recording was repeated twice, if the step count over the two repetitions differed it was repeated until consensus was reached. |
|  | **Variables reported:** Step count |
| **3. Construct** | Step count |
| **4. Measurement property** | Measurement error |
| **5. Components that will be repeated** | 2-min walk with varying activity monitors |
| **6. Source of variation** | Mode of determining step count (3 different activity monitors and observation) |
| **7. Patient population** | Surgical procedure: 1 level fusion for degenerative disc disease, lumbar spinal stenosis and / or degenerative spondylolisthesis, n=40, Age: 65 (13), 65% female |
| **Comprehensive research question:** What is the measurement error of the ActivPAL3, Fitbit Flex and Jawbone UP Move accelerometer derived step count when compared to observed step count during 2 minutes of walking 2-3 days post-operative lumbar spinal fusion surgery. | |

| **Maldaner et al (2020)**^13^ | |
| --- | --- |
| **Elements** |  |
| **1. Type and name of instrument** | Performance-based physical outcome measure: 6-min walk test |
| **2. Operationalization** | **Equipment:** 6WT app, smartphone |
|  | **Personnel:** Not reported |
|  | **Test procedures:** Participants were scheduled for elective spine surgery for lumbar disc herniation, lumbar spinal stenosis, or lumbar degenerative disc disease with or without instability. They were instructed in download and use of app and instructions for testing environment (sufficiently long, straight, and level path with good GPS coverage) were given. Participants were evaluated pre-operatively and asked to perform the 6WT at this time point twice. The 6WT app determines the maximum walking distance in 6 minutes. |
|  | **Variables reported:** Distance walked during 6-min walk test |
| **3. Construct** | Walking capacity |
| **4. Measurement property** | Reliability (test retest) and measurement error |
| **5. Components that will be repeated** | The entire test procedure was repeated |
| **6. Source of variation** | Time (interval between tests not reported) |
| **7. Patient population** | Patients scheduled for elective spine surgery for lumbar disc herniation, lumbar spinal stenosis or lumbar degenerative disc disease with or without instability. n=70, Age: 55.9 (15.4), 39% female |
| **Comprehensive research question:** What is the test retest reliability and measurement error of the 6WT app in people scheduled for elective spine surgery for lumbar disc herniation, lumbar spinal stenosis, or lumbar degenerative disc disease with or without instability? | |

| **Staartjes et al (2018)**^12^ | |
| --- | --- |
| **Elements** |  |
| **1. Type and name of instrument** | Performance-based physical outcome measure: 5-repetition sit to stand test |
| **2. Operationalization** | **Equipment:** Armless chair of standard height (48cm), with a hard seat, firmly placed against a wall. Stopwatch to record time. |
|  | **Personnel:** Not reported |
|  | **Test procedures:** Pre-operatively, the participants were instructed to fold their arms across their chest and to keep their feet flat on the ground. Participants were required to wear stable shoes for the test. To become familiarized with the movement, the participants were asked to stand up fully and sit back down again once without using their upper limbs. If assistance was required, or if the maneuver could not be completed, the test was abandoned. Otherwise, the patients were asked to, starting on the command “go,” stand up fully and sit down again, landing on the seat firmly, 5 times as fast as possible. The participant’s score is the time from the initial command to the completed fifth stand. If the patient was unable to perform the test in 30 seconds, or not at all, this was noted and the test score was recorded as 30 seconds. |
|  | **Variables reported:** Time to complete |
| **3. Construct** | Functional strength and mobility |
| **4. Measurement property** | Reliability (test retest) and measurement error |
| **5. Components that will be repeated** | The entire test procedure was repeated |
| **6. Source of variation** | Time (second test performed after a 30-min interval) |
| **7. Patient population** | Participants were candidates for surgery for lumbar disc herniation, lumbar spinal stenosis, or lumbar degenerative disc disease with or without instability or synovial facet cysts. n=157, Age: 49.90 (14.10), 51% male |
| **Comprehensive research question:** What is the test retest reliability and measurement error of the 5 repetition sit to stand test in people scheduled for elective spine surgery for lumbar disc herniation, lumbar spinal stenosis, or lumbar degenerative disc disease with or without instability or synovial facet cysts? | |

| **Staartjes et al (2019)**^9^ | |
| --- | --- |
| **Elements** |  |
| **1. Type and name of instrument** | Performance-based physical outcome measure: 5-repetition sit to stand test |
| **2. Operationalization** | **Equipment:** Armless chair of standard height (48cm), with a hard seat, firmly placed against a wall. Proprietary video recording software on smartphone, digital timer. |
|  | **Personnel:** Two independent raters. Partner or relative to record unsupervised at home measurement. No further details reported. |
|  | **Test procedures:** The first measurement was obtained during the initial clinical visit (Supervised). Subsequently, patients received instructions for at-home assessment from a licensed physiotherapist. Patients were asked to independently perform two measurements during the 2 days following the initial clinical visit. For the unsupervised measurement, patients performed the 5R-STS as instructed and were timed by a partner or relative. For the tele-supervised measurement, patients were asked to again perform the test, but to produce a video recording of this performance, in place of being timed. Video recordings were obtained using the proprietary video recording software on patient´s smartphones, and the resulting video files were sent per email to a secure server managed by the research nurse and digitally timed. The tele-supervised measurements were timed independently by two raters for assessment of interrater reliability. For all other analyses, mean of the two raters’ test times was used. Patients were asked to wait approximately 1 h between the unsupervised and tele-supervised measurements. |
|  | **Variables reported:** Time to complete 5 repetitions of sit to stand |
| **3. Construct** | Functional strength and mobility |
| **4. Measurement property** | Reliability (1: between measurement tools / environment, 2: inter-rater for tele-supervised) and measurement error |
| **5. Components that will be repeated** | The entire test procedure was repeated at home (unsupervised and tele-supervised) |
| **6. Source of variation** | 1: Supervised in clinic vs Unsupervised at home vs Tele-supervised at home; 2: Two independent raters |
| **7. Patient population** | Patients were scheduled for surgery for lumbar disc herniation, lumbar spinal stenosis, lumbar spondylolisthesis, or degenerative disc disease. n=100, Age: 45.25 (11.90), 44% male. |
| **Comprehensive research question:** What is the test retest reliability of the 5 repetition sit to stand test when measured supervised in the clinic by a physiotherapist and unsupervised at home measured by family / friend in people scheduled for surgery for lumbar disc herniation, lumbar spinal stenosis, lumbar spondylolisthesis, or degenerative disc disease? What is the inter rater reliability and measurement error of the tele-supervised 5 repetition sit to stand test, measured by video recording in people scheduled for surgery for lumbar disc herniation, lumbar spinal stenosis, lumbar spondylolisthesis, or degenerative disc disease? | |

| **Takenaka et al (2023)**^37^ | |
| --- | --- |
| **Elements** |  |
| **1. Type and name of instrument** | Performance-based physical outcome measure: 6-minute walk test distance walked |
| **2. Operationalization** | **Equipment:** 30 m indoor walking path |
|  | **Personnel:** Assessments were performed by a physical therapist |
|  | **Test procedures:** Assessments were performed pre-operatively and post-operatively at 6 and 12 months. The 6-minute walking time was performed along a 30 m path according to established guidelines. The test ended when pain or numbness became too severe for the patient to walk. The patients did not use walking aids whenever possible and the use of a cane was permitted only if they were unable to walk. If a patient could not walk even with a cane, the 6MWD was measured as 0 m and the use of a walker was not permitted. A reliability coefficient of 0.95 was used for the 6MWD test in a previous study. |
|  | **Variables reported:** Distance walked during 6 minutes of walking |
| **3. Construct** | Walking capacity |
| **4. Measurement property** | Measurement error |
| **5. Components that will be repeated** | The entire test procedure was repeated. |
| **6. Source of variation** | Time (Pre-operative and 6 months post-operative) |
| **7. Patient population** | Fusion (56%) and / or decompression (70%) for lumbar spinal canal stenosis, n=126, Age: 72 (7), 59% male |
| **Comprehensive research question:** What is the measurement error of the 6-minute walk test distance when measured pre-operative and 6 months post-operative surgery for lumbar spinal canal stenosis? | |

| **Zeitlberger et al (2022)**^15^ | |
| --- | --- |
| **Elements** |  |
| **1. Type and name of instrument** | Performance-based physical outcome measure: 6-min walk test |
| **2. Operationalization** | **Equipment:** 6WT app, smartphone |
|  | **Personnel:** Not reported |
|  | **Test procedures:** The 6WT app measures the maximum distance (in m) walked within six minutes (6WD) using global positioning system (GPS) coordinates. Both distance walked and time elapsed are continuously displayed on the screen, while the 6WT is conducted. Patients were instructed to press a "flash" button on the app’s user interface in case of appearance and/or first-time significant aggravation in leg or back pain during the test. This marks their time (in sec) and walking distance (in m) to first symptoms. Patients were instructed to continue walking until the six minutes have elapsed, whenever possible. Completed measurements are saved on the patient’s smartphone device with a date and time stamp and may be transferred to a secure online database. |
|  | **Variables reported:** Distance walked, time to first symptoms and distance to first symptoms during 6-min walk test |
| **3. Construct** | Walking capacity |
| **4. Measurement property** | Reliability (test retest) and measurement error |
| **5. Components that will be repeated** | The entire test procedure was repeated |
| **6. Source of variation** | Time (Pre-operative and 6 weeks post-operative) |
| **7. Patient population** | Patients scheduled for elective surgery for lumbar disc herniation, lumbar spinal stenosis or degenerative lumbar disorders with or without instability. n=49, Age: 55.5 (15.8), 41% female |
| **Comprehensive research question:** What is the test retest reliability and measurement error of the 6WT app when measured pre-operative and 6 weeks post-operative surgery for lumbar disc herniation, lumbar spinal stenosis or degenerative lumbar disorders with or without instability? | |

**S6 Appendix 5:** Quality of evidence (GRADE)

| **Physical outcome measure / physical measure** | **Measurement property** | **Risk of Bias** | **Inconsistency** | **Imprecision** | **Indirectness** | **Overall quality of evidence** |
| --- | --- | --- | --- | --- | --- | --- |
| **Impairment-based physical outcome measures** | | | | | | |
| **Physical outcome measure: Active range of movement** | | | | | | |
| Physical measure: Lumbar extension (using Dualer goniometer) | Responsiveness (Construct approach)^33^ | Extremely serious (-3), one study of inadequate | No downgrade (only one study) | No downgrade (n=145) | No downgrade (100% population, fair comparator) | Very low |
| Physical measure: Lumbar, trunk and hip flexion and extension (using computer assisted electronic inclinometer) | Construct validity^2^ | Extremely serious (-3), one study of inadequate | No downgrade (only one study) | -2 (n=33) | No downgrade (100% population, fair comparators) | Very low |
|  | Responsiveness (Construct approach)^2^ | Extremely serious (-3), one study of inadequate | No downgrade (only one study) | -2 (n=33) | No downgrade (100% population, fair comparator) | Very low |
| Physical measure: Lumbopelvic flexion and extension (using Dual bubble inclinometers) | Construct validity^3^ | Extremely serious (-3), one study of inadequate | No downgrade (only one study) | -2 (n=16) | No downgrade (100% population, fair comparators) | Very low |
| Physical measure: Schober test | Responsiveness (Construct approach)^33^ | Extremely serious (-3), one study of inadequate | No downgrade (only one study) | No downgrade (n=145) | No downgrade (100% population, fair comparator) | Very low |
| **Physical outcome measure: Handgrip strength** | | | | | | |
| Physical measure: Handgrip maximum voluntary contraction (using handheld dynamometer) | Construct validity^4–6^ | Very serious (-2), multiple studies of inadequate | No downgrade (consistent rating) | No downgrade (n=474) | No downgrade (100% population, mostly fair comparators) | Low |
| **Physical outcome measure: Gait parameters** | | | | | | |
| Physical measure: Two-step test | Construct validity^7^ | Extremely serious (-3), one study of inadequate | No downgrade (only one study) | No downgrade (n=357) | No downgrade (100% population, fair comparators) | Low |
|  | Criterion validity^7^ | Very serious (-2), one study of doubtful | No downgrade (only one study) | No downgrade (n=357) | No downgrade (100% population, fair comparators) | Low |
| Physical measure: Asymmetry of double support (using RehabGait) | Responsiveness (Construct approach)^34^ | Extremely serious (-3), one study of inadequate | No downgrade (only one study) | -2 (n=29) | No downgrade (100% population, fair comparators) | Very low |
| Physical measure: Stride length (using RehabGait) | Responsiveness (Construct approach)^34^ | Extremely serious (-3), one study of inadequate | No downgrade (only one study) | -2 (n=20) | No downgrade (100% population, fair comparators) | Very low |
| **Performance-based physical outcome measures** | | | | | | |
| **Physical outcome measure: 1-min stair climb** | | | | | | |
| Physical measure: Number of stairs | Responsiveness (Construct approach)^35^ | No downgrade, one study of very good | No downgrade (only one study) | -1 (n=93) | No downgrade (100% population, fair comparators) | Moderate |
| **Physical outcome measure: 5 repetitions sit to stand** | | | | | | |
| Physical measure: Time to complete | Construct validity^8–12^ | Very serious (-2), multiple studies of inadequate | No downgrade (consistent rating) | No downgrade (n=1,003) | No downgrade (100% population, fair comparators) | Low |
|  | Measurement error^9,12^ | Very serious (-2), multiple studies of inadequate | No downgrade (consistent rating) | no downgrade (n=166) | no downgrade (100% population) | Low |
|  | Reliability^9,12^ | Very serious (-2), multiple studies of inadequate | No downgrade (consistent rating) | No downgrade (n=166) | no downgrade (100% population) | Low |
| **Physical outcome measure: 5-min walk test** | | | | | | |
| Physical measure: Distance walked | Responsiveness (Construct approach)^35^ | No downgrade, one study of very good | No downgrade (only one study) | -1 (n=93) | No downgrade (100% population, fair comparators) | Moderate |
| **Physical outcome measure: 6-min walk test** | | | | | | |
| Physical measure: Distance walked | Construct validity^13–16^ | Very serious (-2), multiple studies of inadequate | No downgrade (consistent rating) | No downgrade (n= 218) | No downgrade (100% population, fair comparators) | Low |
|  | Measurement error^13,15,37^ | Serious (-1), multiple studies of doubtful | Serious (-1), two studies indeterminant, one study adequate | no downgrade (n=245) | no downgrade (100% population) | Low |
|  | Reliability (test retest)^13,15^ | Serious (-1), multiple studies of doubtful | No downgrade (consistent rating) | no downgrade (n=119) | no downgrade (100% population) | Moderate |
|  | Responsiveness (Criterion approach)^14,15,36,37^ | Serious (-1), multiple studies of doubtful, one inadequate | No downgrade (consistent rating) | No downgrade (n=265) | No downgrade (100% population, fair comparators) | Low |
|  | Responsiveness (Construct approach)^14–16,36,37^ | Very serious (-2), multiple studies of inadequate | No downgrade (consistent rating) | No downgrade (n=315) | No downgrade (100% population) | Low |
|  | Responsiveness (Patient reported)^38^ | Extremely serious (-3), one study of inadequate | No downgrade (only one study) | -2 (n=49) | No downgrade (100% population, fair comparators) | Very low |
| Physical measure: Distance to first symptoms | Construct validity^15^ | Extremely serious (-3), one study of inadequate | No downgrade (only one study) | -2 (n=49) | No downgrade (100% population, fair comparators) | Very low |
|  | Measurement error^15^ | Very serious (-2), one study of doubtful | No downgrade (only one study) | -2 (n=49) | no downgrade (100% population) | Very low |
|  | Reliability (test retest)^15^ | Very serious (-2), one study of doubtful | No downgrade (only one study) | -2 (n=49) | no downgrade (100% population) | Very low |
|  | Responsiveness (Criterion approach)^15^ | Extremely serious (-3), one study of inadequate | No downgrade (only one study) | -2 (n=49) | No downgrade (100% population, fair comparators) | Very low |
|  | Responsiveness (Construct approach)^15^ | Extremely serious (-3), one study of inadequate | No downgrade (only one study) | -2 (n=49) | No downgrade (100% population, fair comparators) | Very low |
| Physical measure: Time to first symptoms | Construct validity^15^ | Extremely serious (-3), one study of inadequate | No downgrade (only one study) | -2 (n=49) | No downgrade (100% population, fair comparators) | Very low |
|  | Measurement error^15^ | Very serious (-2), one study of doubtful | No downgrade (only one study) | -2 (n=49) | no downgrade (100% population) | Very low |
|  | Reliability (test retest)^15^ | Very serious (-2), one study of doubtful | No downgrade (only one study) | -2 (n=49) | no downgrade (100% population) | Very low |
|  | Responsiveness (Criterion approach)^15^ | Extremely serious (-3), one study of inadequate | No downgrade (only one study) | -2 (n=49) | No downgrade (100% population, fair comparators) | Very low |
|  | Responsiveness (Construct approach)^15^ | Extremely serious (-3), one study of inadequate | No downgrade (only one study) | -2 (n=49) | No downgrade (100% population, fair comparators) | Very low |
| **Physical outcome measure: 10-meter walk test** | | | | | | |
| Physical measure: Walking speed | Construct validity^17^ | Extremely serious (-3), one study of inadequate | No downgrade (all consistent) | -1 (n=94) | No downgrade (100% population, fair comparator) | Very low |
|  | Responsiveness^17^ | Extremely serious (-3), one study of inadequate | No downgrade (only one study) | -1 (n=94) | No downgrade (100% population, fair comparator) | Very low |
| **Physical outcome measure: 50-foot walk test** | | | | | | |
| Physical measure: Time to complete | Responsiveness (Construct approach)^35^ | No downgrade, one study of very good | No downgrade (only one study) | -1 (n=93) | No downgrade (100% population, fair comparators) | Moderate |
| **Physical outcome measure: Modified Sorensen test** | | | | | | |
| Physical measure: Time to exhaustion | Construct validity^18,19^ | Very serious (-2), two studies of inadequate | No downgrade (consistent rating) | -1 (n=69) | No downgrade (100% population, fair comparators) | Very low |
| **Physical outcome measure: Self-paced walking test** | | | | | | |
| Physical measure: Distance walked | Responsiveness (Construct approach)^39^ | Extremely serious (-3), one study of inadequate | No downgrade (only one study) | -2 (n=21) | No downgrade (100% population, fair comparator) | Very low |
| Physical measure: Time walked | Responsiveness (Construct approach)^39^ | Extremely serious (-3), one study of inadequate | No downgrade (only one study) | -2 (n=21) | No downgrade (100% population, fair comparator) | Very low |
| **Physical outcome measure: Timed up and go** | | | | | | |
| Physical measure: Time to complete | Construct validity^8,14,20–24^ | Very serious (-2), multiple studies of inadequate and one doubtful | Serious (-1), one study of sufficient, six studies of indeterminant) | No downgrade (n= 1654) | No downgrade (100% population, fair comparators) | Very low |
|  | Responsiveness (Construct approach)^14,35,40^ | Serious (-1), two studies inadequate, one very good | Serious (-1), two studies of indeterminant, one study of sufficient | No downgrade (n=278) | No downgrade (100% population, fair comparators) | Low |
|  | Responsiveness (Criterion approach)^14^ | Very serious (-2), one study doubtful | No downgrade (only one study) | -2 (n=49) | No downgrade (100% population, fair comparator) | Very low |
| **Physical outcome measure: Treadmill test** | | | | | | |
| Physical measure: Maximum walking distance | Construct validity^25,26^ | Very serious (-2), multiple studies of inadequate | No downgrade (consistent rating) | No downgrade (n=104) | No downgrade (100% population, fair comparators) | Low |
| Physical measure: Maximum walking time | Construct validity^26^ | Extremely serious (-3), one study of inadequate | No downgrade (only one study) | -2 (n=48) | No downgrade (100% population, fair comparator) | Very low |
|  | Reliability (Pre-operative)^44^ | Extremely serious (-3), one study of inadequate | No downgrade (only one study) | -2 (n=28) | no downgrade (100% population) | Very low |
|  | Reliability (Post-operative)^44^ | Extremely serious (-3), one study of inadequate | No downgrade (only one study) | -2 (n=18) | no downgrade (100% population) | Very low |
| Physical measure: Time to first symptoms | Construct validity^26^ | Extremely serious (-3), one study of inadequate | No downgrade (only one study) | -2 (n=48) | No downgrade (100% population, fair comparators) | Very low |
|  | Reliability (Pre-operative)^44^ | Extremely serious (-3), one study of inadequate | No downgrade (only one study) | -2 (n=28) | no downgrade (100% population) | Very low |
|  | Reliability (Post-operative)^44^ | Extremely serious (-3), one study of inadequate | No downgrade (only one study) | -2 (n=18) | no downgrade (100% population) | Very low |
| Physical measure: Distance to first symptoms | Construct validity^26^ | Extremely serious (-3), one study of inadequate | No downgrade (only one study) | -2 (n=48) | No downgrade (100% population, fair comparator) | Very low |
| **Physical outcome measure: Trunk muscle endurance** | | | | | | |
| Physical measure: Repetitive arch-ups until exhaustion | Responsiveness (Construct approach)^33^ | Extremely serious (-3), one study of inadequate | No downgrade (only one study) | No downgrade (n=145) | No downgrade (100% population, fair comparators) | Very low |
| Physical measure: Repetitive sit-ups until exhaustion | Responsiveness (Construct approach)^33^ | Extremely serious (-3), one study of inadequate | No downgrade (only one study) | No downgrade (n=145) | No downgrade (100% population, fair comparators) | Very low |
| **Activity in a natural environment physical outcome measures** | | | | | | |
| **Physical outcome measure: Step count** | | | | | | |
| Physical measure: Steps per day (using Fitbit Flex 2,^29^ Fitbit Charge,^27^ Fitbit Zip,^41^ Mi Band^28^) | Construct validity^27–29^ | Very serious (-2), multiple studies of inadequate | Serious (-1), one study of insufficient, two studies of indeterminant | -1 (n=54-60) | No downgrade (100% population, fair comparators) | Very low |
|  | Responsiveness (Construct approach)^41^ | Extremely serious (-3), one study of inadequate | No downgrade (only one study) | -2 (n=28) | No downgrade (100% population, fair comparators) | Very low |
| Physical measure: Steps detected at thigh (using ActivPAL3, Fitbit Flex, Jawbone UP Move) | Criterion validity^32^ | Serious (-1), multiple studies of doubtful | Serious (-1), two studies of sufficient, one study of insufficient | -2 (n=40) | No downgrade (100%, good comparator) | Very low |
|  | Measurement error^32^ | Very serious (-2), one study of doubtful | No downgrade (only one study) | -2 (n=40) | no downgrade (100% population) | Very low |
| Physical measure: Steps detected at wrist (using Fitbit Flex, Jawbone UP Move) | Criterion validity^32^ | Serious (-1), two studies of doubtful | No downgrade (consistent rating) | -2 (n=40) | No downgrade (100%, good comparator) | Very low |
|  | Measurement error^32^ | Very serious (-2), one study of doubtful | No downgrade (only one study) | -2 (n=40) | no downgrade (100% population) | Very low |
| **Physical outcome measure: Gait Posture Index** | | | | | | |
| Physical measure: Gait Posture Index (using personal device e.g., Apple watch, Garmin or Mi Band 2^42,43^) | Responsiveness (Construct approach)^42,43^ | Very serious (-2), multiple studies of inadequate | No downgrade (consistent rating) | -2 (n=37) | No downgrade (100% population, fair comparator) | Very low |
| **Physical outcome measure: Distance per day** | | | | | | |
| Physical measure: Distance per day (km / day using Fibit Zip^41^ and miles / day using Apple iOS Apple Health activity data from personal device^30^) | Construct validity^30^ | Extremely serious (-3), one study of inadequate | No downgrade (all consistent) | -2 (n=8) | No downgrade (100%, fair comparator) | Very low |
|  | Responsiveness (Construct approach)^41^ | Extremely serious (-3), one study of inadequate | No downgrade (only one study) | -2 (n=28) | No downgrade (100% population, fair comparators) | Very low |
| **Physical outcome measure: Gait cycles** | | | | | | |
| Physical measure: Gait cycles per day (using StepWatch 3) | Construct validity^31^ | Extremely serious (-3), one study of inadequate | No downgrade (only one study) | -2 (n=47) | No downgrade (100% population, fair comparators) | Very low |
| Physical measure: Gait cycles per hour (using StepWatch 3) | Construct validity^31^ | Extremely serious (-3), one study of inadequate | No downgrade (only one study) | -2 (n=47) | No downgrade (100% population, fair comparators) | Very low |
| Physical measure: Gait intensities per day (using StepWatch 3) | Construct validity^31^ | Extremely serious (-3), one study of inadequate | No downgrade (only one study) | -2 (n=47) | No downgrade (100% population, fair comparators) | Very low |

**S6 Appendix 6:** Interpretability

| **Physical outcome measures / physical measures** | **Authors (year)** | **Distribution of scores in study population, Mean (SD) unless otherwise noted** | **% missing items and total scores** | **Floor and ceiling effects** | **Score and change scores for relevant subgroups** | **Minimum important change or difference^a^** |
| --- | --- | --- | --- | --- | --- | --- |
| **Impairment-based physical outcome measures** | | | | | | |
| **Physical outcome measure: Active range of movement** | | | | | | |
| Physical measure:  Lumbar extension (using Dualer goniometer) | Häkkinen et al (2005)^33^ | Goniometer degrees, Med (IQR):  2 months post-operative: 10 (7-13)  14 months post-operative: 12 (10-18) | 2 months: 0%  14 months: 16% due to lost to follow-up | NR | NR | NR |
| Physical measure:  Schober test |  | Cm, Med (IQR):  2 month post-operative: 4 (3-5)  14 months post-operative: 5 (4-5) | 2 months: 0%  14 months: 16% due to lost to follow-up | NR | NR | NR |
| Physical measure:  Lumbar, trunk and hip flexion and extension (using computer-assisted electronic inclinometer) | Mannion et al (2005)^2^ | Pre-operative (Inclinometer degrees):  Hip flexion: 42.1 (18.9)  Trunk flexion: 84.7 (27.1)  Lumbar flexion: 42.8 (15.9)  Hip extension: 13.4 (8.9)  Trunk extension: 22.8 (10.3)  Lumbar extension: 6.9 (5.6)  2 months post-operative:  Hip flexion: 47.2 (16.6)  Trunk flexion: 85.9 (29.0)  Lumbar flexion: 36.7 (13.1)  Hip extension: 13.8 (8.6)  Trunk extension: 21.9 (11.3)  Lumbar extension: 6.7 (6.7) | 0% | NR  Pre-operative Lumbar extension: 6.9 (5.6)  2 months post-operative Lumbar extension: 6.7 (6.7) | Change in lumbar flexion reported in figure format. Others NR | NR |
| Physical measure:  Lumbopelvic flexion and extension (using Dual bubble inclinometer) | Pitino (2000)^3^ | Lumbar flexion (degrees, mean (SD), Range):  Pre-operative: 9.86 (8.84), 0-27  Post-operative: 14.19 (7.55), 0-27  Discharge: 15.87 (7.24), 2.0-30.0  Lumbar extension:  Pre-operative: 12.6 (8.97), 0-30  Post-operative: 12.31 (5.65), 4-25  Discharge: 12.00 (4.36), 5-19 | Pre-operative: 12.5%  Post-operative: 0%  Discharge: 6%  due to poor therapist and patient compliance, and loss to follow-up | NR  Lumbar flexion  Pre-operative: 9.86 (8.84), 0-27  Pre-operative: 12.6 (8.97), 0-30  Lumbar extension  Pre-operative: 12.6 (8.97), 0-30 | NR | NR |
| **Physical outcome measure: Handgrip strength** | | | | | | |
| Physical measure:  Handgrip maximum voluntary contraction (using handheld dynamometer) | Inoue et al (2020)^6^ | 30.2 (9.1) kg | 0% | NR | NA | NR |
|  | Kwon et al (2020)^4^ | 17.3 kg (unclear if pre- or post-operative measurement) | 0% | NR | NR | NR |
|  | Kwon et al (2020)^5^ | NR | NR | NR | Low hand grip: <26 kg for men, < 18 kg for women | NR |
| **Physical outcome measure: Gait parameters** | | | | | | |
| Physical measure:  Two-step test | Fujita et al (2019)^7^ | 1.04 (0.22) | 0% | NR | NA | NR MIC/MID; Results identified Two-step test score of 0.93 as cut-off value for short stride length |
| Physical measure:  Asymmetry of double support (using RehabGait system) | Loske et al (2018)^34^ | Mean (SD), Range  Pre-op: 14.3 (13.5), 0.2 - 55.4  10 weeks post-operative: 11.4 (10.2), 0.1 - 39.9  12 months post-operative: 15.2 (12.9), 1.9 - 62.1 | 10 weeks: 17% due to intolerance of sensor straps, insufficient walking distance, other reasons for study withdrawal  12 months: 43% due to other pathology, persistent pain, lost to follow-up | NR  Pre-op: 14.3 (13.5), 0.2 - 55.4  10 weeks post-operative: 11.4 (10.2), 0.1 - 39.9  12 months post-operative: 15.2 (12.9), 1.9 - 62.1 | Control Group: 13.1 (11.1), Range: 0.9 - 53.1  Change scores: NR | NR |
| Physical measure:  Stride length (using RehabGait system) |  | Mean (SD), Range (m)  Pre-operative: 1.18 (0.26), 0.60 - 1.78  10 weeks post-operative: 1.21 (0.20), 0.78 - 1.72  12 months post-operative: 1.20 (0.22), 0.78 -1.82 | 10 weeks: 17% due to intolerance of sensor straps, insufficient walking distance, other reasons for study withdrawal  12 months: 43% due to other pathology, persistent pain, lost to follow-up | NR | Control group: 1.34 (0.26), Range: 0.70 - 1.61  Change scores: NR | NR |
| **Performance-based physical outcome measures** | | | | | | |
| **Physical outcome measure: 1-min stair climb** | | | | | | |
| Physical measure:  Number of stairs | Jakobsson et al (2019)^35^ | Pre-operative (# steps):  Completed 6-month physical tests: 105.3 (24.8)  Improved: 95.3 (26.9) Unchanged: 114.1 (20.7)  6 months:  Completed 6-month physical tests: NR  Improved: 125.0 (24.3)  Unchanged: 127.1 (22.3) | 21% for responsiveness due to no surgery, study withdrawal, scheduling, pain in other areas, lost to follow-up  53% for MIC analysis due to deterioration on GPE, did not complete GPE | NR | Change score (6 months – pre-operative scores):  Improved: 29.7 (23.6)  Unchanged: 13.0 (19.4) | Results identified: MIC absolute (95% CI): 20.0 steps (10.5 to 48.0)  MIC relative (95% CI): 12.5% (7.2 to 48.4) |
| **Physical outcome measure: 5 repetitions sit to stand** | | | | | | |
| Physical measure:  Time to complete | Klukowska et al (2020)^11^ | NR | 17% due to incomplete PROM questionnaires | NR | No OFI (≤10.4 sec): 42%  Mild OFI (10.5 - 15.2 sec): 33%  Moderate OFI (15.3 -22.0 sec): 14%  Severe OFI (>22.0 sec): 10% | NR |
|  | Master et al (2020)^8^ | 34.8 (22.5) sec | 0% | NR | Change Score  Mean (95% CI): -12.5 (-17.3 to -7.8) | Reported MCID for 5-chair stand test is 2.3 sec in patients with vestibular conditions |
|  | Staartjes et al (2018)^12^ | 13.32 (7.87) sec  Retest: NR for surgical cohort | 2% due to inability to preform 5 repetition sit to stand test independently | NR | Control: 6.44 (1.68)  Not significant OFI (≤10.4 sec): 50%  Mild OFI (10.5 - 15.2 sec): 25%  Moderate OFI (15.3 - 22.0 sec): 15%  Severe OFI (>22.0 sec): 10% | NR MIC/MID; Results identified upper limit of normal to distinguish those with and without OFI as 10.35 sec |
|  | Staartjes et al (2019)^9^ | Clinic: 13.75 (6.84) sec  Unsupervised: 13.54 (7.11) sec  Tele-supervised: 13.25 (5.66) sec | 2% for clinic assessment, 27% for unsupervised assessment, 47% for tele-supervised assessment due to missing scores | NR | NA | NR |
|  | Staartjes et al (2020)^10^ | 13.4 (6.4) sec | 10% due to missing data | NR | OFI (>10.5 sec): 59% Severe OFI (>22.0 sec): 12% | NR |
| **Physical outcome measure: 5-min walk test** | | | | | | |
| Physical measure:  Distance walked | Jakobsson et al (2019)^35^ | Pre-operative (m):  Completed 6-month physical tests: 422.3 (82.4)  Improved: 403.2 (94.2)  Unchanged: 445.5 (73.3)  6 months:  Completed 6-month physical tests: NR  Improved: 484.9 (66.7)  Unchanged: 474.7 (93.0) | 21% for responsiveness due to no surgery, study withdrawal, scheduling, pain in other areas, lost to follow-up  53% for MIC analysis due to deterioration on GPE, did not complete GPE | NR | Change score (6 months – pre-operative scores):  Improved: 81.7 (75.1)  Unchanged: 29.2 (59.9) | Results identified: MIC absolute (95% CI): 45.5m (8.5 to 62.0)  MIC relative (95% CI): 9.0% (4.5 to 11.8) |
| **Physical outcome measure: 6-min walk test** | | | | | | |
| Physical measure:  Distance walked | Maldaner et al (2020)^13^ | 370 (137) m | 0% | NR | Control group: 586 (103) m  No OFI (z-score >-1): 31%  Mild OFI (z-score -1 to -1.9): 17%  Moderate OFI (z-score -2 to -2.9): 23%  Severe OFI (z-score ≤ -3): 29% | NR |
|  | Maldaner et al (2021)^14^ | Pre-operative: 401 (129) m  6 weeks post-operative: 495 (129) m | 2% due to no follow up | Authors reported: 6-min walk test subject to a certain floor effect because a considerable proportion of patients (approximately 50%–60%) score within the 95^th^ percentile of the normal population. | Change from Pre-operative to Post-operative:  Distance: 94 (109) m  z-score: 0.95 (1.2) | NR MIC/MID; results identified cut off value of 1.06 in z-score change as threshold to discriminate patient satisfaction with treatment |
|  | Sosnova et al (2021)^38^ | NR | 2% due to incomplete baseline and follow-up | NR | NR | NR |
|  | Takenaka et al (2022)^36^ | Pre-operative: 341.6 (170.8) m  6 months post-operative: 482.7 (92.4) m | 69% due to exclusion criteria, other reasons, cancelled appointments, other surgeries or illnesses during follow-up period | NR | Change mean (SD):  Overall: 141.1 (151.4)  Women: 174.6 (141.9)  Men: 119 (156.1) Fusion: 42.5 (37.9)  Decompression: 51.5 (23.9)  Change in ODI (>12.8): 159.6 (158.8)  No change in ODI: 51.4 (52.5) | Results identified distribution-based MDC for 6-min walk distance: 105.9m  Anchor-based approach (ODI change >12.8): AUC cut point: 50m to discriminate responders vs non-responders |
|  | Takenaka et al (2023)^37^ | Pre-operative: 311.5 (154.4) m  6 months post-operative: 449.0 (86.5) m  12 months post-operative: 443.6 (97.1) m | 171 enrolled, 45 excluded (26%, lost to follow-up, treated for other diseases, unknown diagnoses, incorrectly marked ODI, treated at other hospitals, refusal to respond. 6 month response rate: 68.5% (85/124), 12 month response rate: 71.4% (90/126) | Authors reported a ceiling effect may have occurred in cases with a high pre-operative 6-min walk distance | 6 month change: Total: 138.7 (144.0) m  ODI responders: 173.1 (148.7) m  ODI non-responders: 61.9 (97.1) m  12 month change:  Total: 132.2 (141.7) m  ODI responders: 168.4 (146.3) m  ODI non-responders: 39.1 (69.7) m | Results identified distribution-based MDC for 6-min walk distance at 6 months: 95.7m  Anchor-based approach (ODI >12.8): AUC cut point: 102.3m at 6 months and 57.5m at 12 months to discriminate responders vs non-responders. Propose 100m as MCID for 6-minute walk test after LSS surgery. |
|  | Zeitlberger et al (2022)^15^ | Pre-operative: 401 (129) m  6 weeks post-operative: 495 (129) m | 2% due to incomplete follow-up assessment | NR | NR | NR |
|  | Ziga et al (2023)^16^ | Pre-operative: 378 (137) m  6 weeks post-operative: 490 (126) m  3 months post-operative: 518 (112) m | 71% with complete data at 6 weeks and 3 months post-operative | Detailed analysis of floor and ceiling effects of patient reported measures, NR for 6-min walk | Change between pre-operative and 6 weeks post-operative: 112 (119).  Change between 6 weeks and 3 months post-operative: 28 (80) | NR |
| Physical measure:  Distance to first symptoms | Zeitlberger et al (2022)^15^ | Pre-operative: 227 (161) m  6 weeks post-operative: 431 (194) m | 2% due to incomplete follow-up assessment | Authors reported possible ceiling effects as 29% pre-operatively and 69% post-operatively did not experience symptoms during 6-min walk test, which was assigned 6-min walk distance as score | NR | NR |
| Physical measure:  Time to first symptoms | Zeitlberger et al (2022)^15^ | Pre-operative: 192 (127) sec  Post-operative: 304 (102) sec | 2% due to incomplete follow-up assessment | Authors reported possible ceiling effects as 29% pre-operatively and 69% post-operatively did not experience symptoms during 6-min walk test which was assigned 360 sec as score. | NR | NR |
| **Physical outcome measure: 10-meter walk test** | | | | | | |
| Physical measure: Walking speed | Wada et al (2022)^17^ | Pre-operative: 0.97 (0.27) m/s  12 months post-operative: 1.16 (0.26) m/s | 15% at 12 months due to paralyzed after surgery, developed cancer, did not attend follow-up outpatient visits | NR | NR | NR |
| **Physical outcome measure: 50-foot walk test** | | | | | | |
| Physical measure:  Time to complete | Jakobsson et al (2019)^35^ | Pre-operative (sec):  Completed 6-month physical tests: 9.2 (2.8)  Improved: 9.9 (3.8)  Unchanged: 8.6 (2.4)  6 months:  Completed 6-month physical tests: NR  Improved: 7.7 (1.5)  Unchanged: 8.3 (2.4) | 21% for responsiveness due to no surgery, study withdrawal, scheduling, pain in other areas, lost to follow-up  53% for MIC analysis due to deterioration on GPE, did not complete GPE | NR | Change score (6 months – pre-operative scores):  Improved: −2.2 (3.3)  Unchanged: −0.3 (1.5) | Results identified: MIC absolute (95% CI): −0.6 s (− 0.7 to − 0.2)  MIC relative (95% CI): −6.1% (−7.1 to −3.4) |
| **Physical outcome measure: Modified Sorensen test** | | | | | | |
| Physical measure:  Time to exhaustion | Dedering et al (2006)^18^ | Pre-operative (sec):  All: 175 (72)  Men: 201 (58)  Women: 117 (68)  4 weeks post-operative:  All: 187 (86)  Men: 216 (78)  Women: 121 (70) | Pre-operative: 5% due to inability to complete test because of severe back / leg pain.  Post-operative: 12% due to inability to complete test because of back pain or other health conditions precluded follow up | NR | Reported control data from previous study:  M: 385 (127)  W: 380 (144) | NR |
|  | Dedering (2012)^19^ | All: 231 (86) sec  Men: 245 (84) sec  Women: 197 (87) sec | 40% due to not wanting to participate, lack of time, presence of co-morbidities, location | NR | NR | NR |
| **Physical outcome measure: Self-paced walking test** | | | | | | |
| Physical measure:  Walking distance | Tomkins-Lane et al (2020)^39^ | Median (IQR) (m)  Pre-operative: 361.5 (135.4 - 736.1)  Post-Operative: 1911.1 (431.1 - 2210.4) | 0% | NR | NR | NR |
| Physical measure:  Walking time | Tomkins-Lane et al (2020)^39^ | Median (IQR) (sec)  Pre-operative: 383.0 (200.5 - 699.0)  Post-operative: 1800.0 (506.0 - 1800.0) | 0% | NR  Test limited to 1800 sec  Post-operative: 1800.0 (506.0 - 1800.0) sec | NR | NR |
| **Physical outcome measure: Timed up and go** | | | | | | |
| Physical measure:  Time to complete | Corniola et al (2016)^22^ | NR | 0% | NR | No TUG impairment: 60%  Mild TUG impairment:  13%  Moderate TUG impairment: 14%  Severe TUG impairment: 13% | NR |
|  | Gautschi et al (2016)^23^ | 11.3 (5.8) sec | 0% | NR | Control group: 6.03 (2.36) sec | NR MIC/MID; Results identified upper limit of normal as 11.52 sec |
|  | Gautschi et al (2016)^40^ | Pre-operative: 10.3 (6.3) sec  3 days post-operative: 9.5 (4.3) sec  6 weeks post-operative: 6.5 (2.8) sec | 0% | NR | At 6 weeks, 2% without OFI pre-operatively had mild OFI; 100% with mild OFI pre-operatively remained unchanged; of those with moderate OFI pre-operatively, 84% had no OFI, 5% had mild, moderate and severe OFI; of those with severe OFI pre-operatively, 57% had severe OFI, 29% had mild OFI, 14% had moderate OFI. | Reported MCID for TUG is 3.4 sec |
|  | Jakobsson et al (2019)^35^ | Pre-operative (sec):  Completed 6-month physical tests: 7.8 (3.0)  Improved chair rise: 9.1 (4.4)  Unchanged chair rise: 7.1 (1.6)  Improved walking: 9.0 (SD NR)  Unchanged walking: 6.9 (1.6)  6 months:  Completed 6-month physical tests: Not reported  Improved chair rise: 5.7 (1.2)  Unchanged chair rise: 6.4 (1.9)  Improved walking: 5.8 (SD NR)  Unchanged walking: 6.4 (1.9) | 21% for responsiveness due to no surgery, study withdrawal, scheduling, pain in other areas, lost to follow-up  53% for MIC analysis due to deterioration on GPE, did not complete GPE | NR | Change score (6 months – pre-operative scores):  GPEwalking  Improved: −3.2 (4.1)  Unchanged: −0.5 (1.6)  GPEchair rise Improved: −3.4 (4.0)  Unchanged: −0.7 (1.6) | Results identified: GPEwalking:  MIC absolute (95% CI): −1.3 s (−2.4 to − 0.5)  MIC relative (95% CI): −17.3% (−29.4 to − 10.2)  GPEchair rise:  MIC absolute (95% CI): −1.3 s (− 2.4 to − 0.3)  MIC relative (95% CI): −17.6% (− 20.7 to − 10.2) |
|  | Maldaner et al (2021)^14^ | Pre-operative: 10.44 (4.37) sec  6 weeks post-operative: 8.47 (3.38) sec | 2% due to no follow up | Timed up and go is subject to a certain floor effect because a considerable proportion of patients (approximately 50%–60%) score within the 95^th^ percentile of the normal population. | Change from Pre-operative to Post-operative:  Test time: −2.07 (3.07) sec  z-score: 1.37 (2.43) | Reported clinically meaningful change for TUG is 3.4 sec.  Results identified cut off value of  -1.90 in z-score change as threshold to determine patient satisfaction with treatment |
|  | Master et al (2020)^8^ | 15.5 (8.1) sec | 0% | NR | Change Score  Mean (95% CI): -4.4 (-5.9 to -2.8) | Reported MCID for TUG is between 1.2-1.4 sec in adults with osteoarthritis and MIC is reduction in 1.3 sec for patients undergoing lumbar fusion |
|  | Staartjes et al (2022)^21^ | TUG test time (sec):  CCI 0: 10.62 (5.94)  CCI 1: 15.15 (15.65)  CCI 2: 10.75 (4.58)  CCI 3: 12.22 (6.86)  CCI 4: 13.27 (6.39)  ASA 1: 11.25 (8.68)  ASA 2: 10.82 (5.22)  ASA 3: 15.53 (18.10) | 0% | NR | Change scores NA as cross-sectional analysis | NR |
|  | Stienen et al (2017)^24^ | TUG t-score:  No OFI: 109.1 (8.2)  Mild OFI: 130.8 (5.0)  Moderate OFI: 147.8 (9.0)  Severe OFI: 221.3 (158.0) | 0.8% due to incomplete data | A ceiling effect of the OFI BSS could prevent discriminating between patients with high and extremely high disability. | No OFI (≤11.5 sec): 61%  Mild OFI (11.5 - <13.4 sec): 13%  Moderate OFI (13.5-18.4 sec): 15%  Severe OFI (>18.4 sec):  11% | Reported MCID for TUG is 3.4 sec in patients undergoing surgical treatment for lumbar degenerative disc disease |
|  | Stienen et al (2021)^20^ | 10.8 (4.4) sec, Range: 4.8 - 26.7 | 0% | When applying the OFI baseline severity stratification, 35 patients (50%) had TUG test results within the 99th percentile of the normal population (no OFI). | No OFI: 50%  Mild OFI: 17%  Moderate OFI: 20%  Severe OFI: 13% | Reported MCID for TUG is 3.4 sec in patients undergoing surgical treatment for lumbar degenerative disc disease |
| **Physical outcome measure: Treadmill test** | | | | | | |
| Physical measure:  Maximum walking distance | Herno et al (1999)^25^ | No stenosis: 515 m  Stenosis: 470 m | 10% due to inability to perform test secondary to coexisting diseases | NR | NR | NR |
|  | Prasad et al (2016)^26^ | Pre-operative: 230.21 (68.14) m  Post-operative: 446.88 (85.53) m | 4% due to co-morbidities following surgery | NR | NR | NR |
| Physical measure:  Distance to first symptoms | Prasad et al (2016)^26^ | Pre-operative: 66.75 (47.08) m  Post-operative: 291.74 (75.39) m | 4% due to co-morbidities following surgery | NR  Pre-operative: 66.75 (47.08) m | NR | NR |
| Physical measure:  Maximum walking time | Deen et al (2000)^44^ | Median (IQR) (min):  Pre-operative:  1.2 mph: 5.3 (2.0 - 9.3)  Preferred speed: 3.5 (1.7 - 8.5)  Post-operative:  1.2 mph: 15 (15 - 15)  Preferred speed: 5* (10.1 - 15)  *As reported | Pre-operatively, 14% at 1.2 mph, 4% at preferred speed.  Post-operatively: 17% at 1.2 mph, 0% at preferred speed.  All due to inability to perform test | NR  Pre-operative at 1.2 mph: 14% unable to perform test  Post-operative: 78% (1.2 mph) and 72% (preferred speed) reached end of test time (15 min) | Pre-operative test retest mean (SD) difference:  1.2 mph: - 0.72 (2.1)  Preferred speed: -0.08 (1.4)  Post-operative: NR | NR |
|  | Prasad et al (2016)^26^ | Pre-operative: 408.96 (121.68) sec  Post-operative: 802.32 (152.42) sec | 4% due to co-morbidities following surgery | NR | NR | NR |
| Physical measure:  Time to first symptoms | Deen et al (2000)^44^ | Median (IQR) (min):  Pre-operative:  1.2 mph: 0 (0 - 1.6)  Preferred speed: 0 (0 - 1.3)  Post-operative:  1.2 mph: 15 (10.8, 15)  Preferred sped: 15 (1.5, 15) | Pre-operatively, 14% missing at 1.2 mph, 4% missing at preferred speed.  Post-operatively: 17% missing at 1.2 mph and 0% missing at preferred speed.  All due to inability to perform test | NR  Pre-operative: At 1.2 mph, 14% unable to perform. 46% (1.2 mph) and 64% (preferred speed) had symptoms at start of test.  Post-operative: 61% (1.2 mph and preferred speed) reached end of test without symptoms. | Pre-operative test retest mean (SD) difference:  1.2 mph -0.42 (1.6)  Preferred speed: 0.04 (0.8)  Post-operative; NR | NR |
|  | Prasad et al (2016)^26^ | Pre-operative: 117.67 sec (79.71)  Post-operative: 517 sec (153.3) | 4% due to co-morbidities following surgery | NR | NR | NR |
| **Physical outcome measure: Trunk muscle endurance (tests of repetition)** | | | | | | |
| Physical measure:  Repetitive arch-ups until exhaustion | Häkkinen et al (2005)^33^ | Median (IQR) (repetitions):  2 months post-operative: 30 (12 - 41)  14 months post-operative: 37 (23 - 53) | Missing scores:  2 months: 0%  14 months: 16% due to lost to follow-up | NR | NR | NR |
| Physical measure:  Repetitive sit-ups until exhaustion | Häkkinen et al (2005)^33^ | Median (IQR) (repetitions):  2 months post-operative: 20 (2 - 31)  14 months post-operative: 27 (17 - 39) | Missing scores:  2 months: 0%  14 months: 16% due to lost to follow-up | NR | NR | NR |
| **Activity in a natural environment physical outcome measures** | | | | | | |
| **Physical outcome measure: Step count** | | | | | | |
| Physical measure:  Steps per day (using Fitbit Flex 2,^29^ Fitbit Charge,^27^ Fitbit Zip,^41^ Mi Band^28^) | Bienstock et al (2022)^29^ | Pre-operative: 5536 (1314)  NR Steps per day post-operatively | Missing scores:  2 weeks: 15%  1 month: 10%  3 months: 20%  6 months: 30%  due to discharge to rehabilitation facility, death and COVID-19 pandemic | NR | NR | NR |
|  | Kim et al (2019)^27^ | Post-operative day 1: 773 (1199.49)  Post-operative day 2: 1524.27 (2232.13)  Post-operative day 3: 2040.73 (1498.26)  Post-operative day 4: 3081.82 (1982.68)  Post-operative day 5: 3790.68 (2006.05)  Post-operative day 6: 4032.64 (2334.21)  Post-operative day 7: 5412 (2712.44) | 19% due to non-compliance, malfunctioning monitor, and lost to follow-up | NR  Post-operative day 1: 773 (1199.49)  Post-operative day 2: 1524.27 (2232.13) | NR | NR |
|  | Mobbs et al (2016)^41^ | Pre-operative: 5255 (2883)  1 month: 4574 (2186)  2 months: 7135 (3112)  3 months: 8312 (4218) | 7% due to losing monitor | NR | NR | NR |
|  | Stienen et al (2020)^28^ | Pre-operative: 4403 (1936)  3 months: 4679 (2265)  12 months: 3312 (2228) | 19% due to non-compliance with study protocol (NR sample in lumbar cohort) | NR | NR | NR |
| Physical measure:  Steps detected at thigh (using ActivPAL3, Fitbit Flex, Jawbone UP Move) | Gilmore et al (2020)^32^ | ActivPAL3: 145 (54)  Fitbit Flex: 110 (82)  Jawbone: 36 (58) | 0% | NR  Jawbone: 36 (58) | NA | NR |
| Physical measure:  Steps detected at wrist (using Fitbit Flex, Jawbone UP Move) | Gilmore et al (2020)^32^ | Fitbit Flex:  Wrist total: 46 (69)  No gait aid: 75 (84)  Gait aid: 17 (44)  Jawbone:  Wrist total: 38 (81)  No gait aid: 95 (107)  Gait aid: No steps detected | 0% | NR  Fitbit Flex:  Wrist total: 46 (69)  No gait aid: 75 (84)  Gait aid: 17 (44)  Jawbone:  Wrist total: 38 (81)  No gait aid: 95 (107) | NA | NR |
| **Physical outcome measure: Gait Posture Index** | | | | | | |
| Physical measure:  Gait Posture Index (using personal device e.g., Apple watch, Garmin or Mi Band 2^42,43^) | Ghent et al (2020)^42^ | Pre-operative: 59.33 (16.06), Range: 30-96  Post-operative: 85.75 (9.22), Range: 63-100 | 0% | NR  Post-operative: 85.75 (9.22), Range: 63-100 | Mean (SD) difference: 26.42 (14.21), CI 95%: 20.42 to 32.42 | NR |
|  | Mobbs et al (2019)^43^ | Pre-operative: 55.1754 (17.44204), Range: 28.12 - 82.33  Post-operative: 75.9608 (11.10504), Range: 52.91 - 87.91 | 0% | NR | Mean (SD) difference: 20.78538 (17.44097) | NR |
| **Physical outcome measure: Distance per day** | | | | | | |
| Physical measure: Distance per day (km / day using Fibit Zip^41^ and miles / day using Apple iOS Apple Health activity data from personal device^30^) | Mobbs et al (2016)^41^ | Kilometers / day:  Pre-operative: 3.8 (2.2)  1 month: 3.4 (1.7)  2 month: 5.3 (2.5)  3 month: 6.2 (3.6) | 7% due to losing monitor | NR | NR | NR |
|  | Voglis et al (2022)^30^ | Data only presented in figure format | 2% due to incomplete data | NR | Data only presented in figure format | NR |
| **Physical outcome measure: Gait cycles** | | | | | | |
| Physical measure:  Gait cycles per day (using StepWatch 3) | Schulte et al (2010)^31^ | Mean (SD provided only in figure):  Pre-operative: 3578  3 months post-operative: 4145  12 months post-operative: 4335 | 6% due to lost to follow-up | NR | 3 months change: +15.8%  12 months change: +21.2% | NR |
| Physical measure:  Gait cycles per hour (using StepWatch 3) | Schulte et al (2010)^31^ | Pre-operative: 277 (127)  3 months post-operative: 323 (144)  12 month post-operative s: 336 (137) | 6% due to lost to follow-up | NR | NR | NR |
| Physical measure:  Gait intensities per day (using StepWatch 3) | Schulte et al (2010)^31^ | Pre-operative: 3.2 (3.8) %  3 months post-operative: 5.9 (6.4) %  12 months post-operative: 4.9 (3.9) % | 66% due to lost to follow-up | NR | Pre-operative: 3.2 (3.8%)  3 months post-operative: 5.9 (6.4%) | NR |

^a^ “Results identified” indicates result of MIC / MID analysis in research study. “Reported” indicates study reporting of MIC / MID, based on prior research, and used for analysis / interpretation of data.

Response shift not relevant for physical outcome measures.

GPE, Global perceived effect; IQR, Interquartile range; MIC, Minimum important change; MID, Minimum important difference; MCID, Minimal clinically important difference; NA, Not applicable; NR, Not reported; OFI, Objective functional impairment; SD, Standard deviation.

**S6 Appendix 7:** Feasibility

| **Physical outcome measure / physical measure** | **Authors (year)** | **Ease of administration ^a^** | **Time to complete ^b^** | **Ease of standardization ^c^** | **Ease of score calculation ^d^** | **Required equipment and cost ^e^** |
| --- | --- | --- | --- | --- | --- | --- |
| **Impairment-based physical outcome measures** | | | | | | |
| **Physical outcome measure: Active range of movement** | | | | | | |
| Physical measure: Lumbar extension | Häkkinen et al (2005)^33^ | Subjects were in the supine position and passively arched their back by extending the arms. (As reported by authors) | NR | NR | Dualer goniometer placed on same marks used in Schober test and were 10cm apart | Dualer goniometer Cost: NR |
| Physical measure: Schober test | Häkkinen et al (2005)^33^ | NR | NR | NR | NR | NR |
| Physical measure: Lumbar, trunk and hip flexion and extension | Mannion et al (2005)^2^ | All tests of spinal mobility were carried out at least 2 hr after the getting up in the morning. Bony landmarks first determined by palpation and marked on the skin. The device is guided along the spine, slightly paravertebrally, from a start position at the spinous process of C7 to an end position at S3. | NR | The positions were described and demonstrated by the investigator, then performed 3 times by patient. Patient was instructed to move at a speed of their choosing, move as far as they possibly could without causing intolerable pain and hold the end position for about 3 seconds. | Distance and angle measures sent to base station on personal computer to calculate the relative positions of the sacrum and vertebral bodies. The best of 2 trials were averaged for analysis. | Spinal Mouse system (computer-assisted electronic inclinometer).  Cost: NR |
| Physical measure: Lumbopelvic flexion and extension | Pitino (2000)^3^ | Skin markings made at midpoint between PSISs, 10cm above (T12) and 5 cm below (S2). | NR | Participants instructed in warm-up including 2 repetitions of lumbar flexion, extension, sidebend and rotation bilaterally within pain-free range of motion. They were then asked to forward bend until pain was first experienced or increase in level of resting pain. | Bubble inclinometers placed at T12 and S2 with measurements at S2 subtracted from measurement at T12 | Dual bubble inclinometers  Cost: NR |
| **Physical outcome measure: Handgrip strength** | | | | | | |
| Physical measure: Handgrip maximum voluntary contraction | Inoue et al (2020)^6^ | Two tests were performed on both right and left hands with a brief rest between trials. | NR | Participants gripped dynamometer as hard as possible for 3 sec without pressing instrument against body or bending at the elbow with the arm straight by their side in the standing position. | Best performance was used for the analysis. | Handheld dynamometer  Cost: NR |
|  | Kwon et al (2020a)^4^ | Test repeated 3 times on both hands. | NR | Instructed to squeeze handle as hard as possible for 3 seconds. | Maximum contractile force was recorded in kilograms. Highest value used in analysis. | JAMAR plus+ handgrip dynamometer  Cost: NR |
|  | Kwon et al (2020b)^5^ | Test was performed 3 times on both hands. | NR | Instructed to squeeze handle as hard as possible for 3 seconds. | Maximum contractile force was recorded pre-operatively. Highest value of 3 measurements used in analysis. | JAMAR plus+ handgrip dynamometer  Cost: NR |
| **Physical outcome measure: Gait parameters** | | | | | | |
| Physical measure: Two-step test | Fujita et al (2019)^7^ | Two trials performed | NR | Patient starts from standing line and moves two strides forward with maximum stride. If the patient succeeds in keeping the final standing position longer than 3 seconds without any additional steps, the test is defined as complete. | Distance is measured by Physical Therapist then normalized by dividing it by the patient’s height. Best result of 2 trials is recorded. | NR |
| Physical measure: Asymmetry of double support | Loske et al (2018)^34^ | Inertial sensors were attached to lateral aspect of shoe, lower and upper legs and on the pelvis overlying L5. Gait parameters measured during a 6-min walk test conducted on a 3-m wide and 30-m long well-illuminated flat hallway. | Set up: NR Administer: 6 min | Participants instructed to walk as far as possible back and forth along the course for 6 minutes. | Manufacturer’s software was used to calculate gait phases for each leg for all measurement intervals. Gait asymmetry was defined as 100*\|right-left\| / (0.5 * (\|right+left\|)) | RehabGait system, comprising 7 inertial sensors each containing a triaxial accelerometer, gyroscope and magnetometer  Cost: NR |
| Physical measure: Stride length | Loske et al (2018)^34^ | Inertial sensors were attached to lateral aspect of shoe, lower and upper legs and on the pelvis overlying L5. Gait parameters measured during a 6-min walk test conducted on a 3-m wide and 30-m long well-illuminated flat hallway. | Set up: NR Administer: 6 min | Participants instructed to walk as far as possible back and forth along the course for 6 minutes. | Manufacturer’s software was used to calculate the spatiotemporal gait parameters. | RehabGait system, comprising 7 inertial sensors each containing a triaxial accelerometer, gyroscope and magnetometer  Cost: NR |
| **Performance-based physical outcome measures** | | | | | | |
| **Physical outcome measure: 1-min stair climb** | | | | | | |
| Physical measure: Number of stairs | Jakobsson et al (2019)^35^ | The staircase was straight with 10 steps (16cm high) and handrails on both sides (patient allowed to use). Handrails were too far apart to be used at same time. | Set up: NR Administer: 1 min | The patient was asked to climb up and down a flight of stairs for one minute. | Total number of steps was recorded. | Staircase  Cost: NR |
| **Physical outcome measure: 5 repetitions sit to stand** | | | | | | |
| Physical measure: Time to complete | Klukowska et al (2020)^11^ | Participants (wearing stable shoes) sat on an armless chair of standard height (48 cm) with a hard seat, firmly placed against a wall. If assistance was required, the test was abandoned. | NR | Participants were instructed to fold their arms across their chest and to keep their feet flat on the ground. To become familiar with the maneuver, participants were asked to once stand up fully and sit back down again without any assistance from their upper limbs. Participants were asked to stand up fully and sit down again, landing on the seat firmly, 5 times as fast as possible, starting on the command “go.” | Using a stopwatch, the examiner timed the 5 repetitions from the initial command to the completed fifth stand. This time was recorded as the participant’s score. If the patient performed the test in 30 seconds or more, this timing was captured, and the test score was recorded as 30 sec. | Chair, stopwatch  Cost: NR |
|  | Master et al (2020)^8^ | NR | Set up: NR  Administer: Mean (SD) scores: 34.8 (22.5) sec | Patients sit down and stand up from a chair 5 times with arms folded. | Test recorded in seconds | Chair  Cost: NR |
|  | Staartjes et al (2018)^12^ | Participants sat on an armless chair of standard height (48 cm) and with a hard seat, firmly placed against a wall. Participants were required to wear stable shoes. If assistance was required, or if the maneuver could not be completed, the test was abandoned. | Set up: NR  Administer: Mean (SD) scores: 13.32 (7.87) sec | Participants were instructed to fold their arms across their chest and to keep their feet flat on the ground. To become familiarized with the movement, the participants were asked to stand up fully and sit back down again once without using their upper limbs. Otherwise, patients were asked to, starting on the command “go,” stand up fully and sit down again, landing on the seat firmly, 5 times as fast as possible. | Using a stopwatch, the 5 repetitions from the initial command to the completed fifth stand was timed. This time was recorded as the participant’s score. If the patient was unable to perform the test in 30 sec, or not at all, this was noted and the test score was recorded as 30 sec. | Chair, Stopwatch  Cost: NR |
|  | Staartjes et al (2019)^9^ | NR | Set up: NR  Administer: Mean (SD) scores in clinic: 13.75 (6.84) sec | Test performed according to protocol described by Jones et al (2013). Patients received instructions for at-home assessment from a licensed Physiotherapist. | Clinic: NR  Supervised: Timed by partner  Tele-supervised: Video recordings were obtained using the proprietary video recording software on patient’s smartphones. Video files were sent per email to a secure server and digitally timed. | Chair, smartphone with proprietary video recording software, digital timer  Cost: NR |
|  | Staartjes et al (2020)^10^ | Participants sat on an armless chair of standard height (48 cm) with a hard seat, firmly placed against a wall, and were required to wear stable shoes. If assistance was required, or if the maneuver could not be completed, the test was abandoned. | Set up: NR  Administer: Mean (SD) scores: 13.4 (6.4) sec | The participants were instructed to fold their arms across their chest, and to keep their feet flat on the ground. To become familiar with the maneuver, participants were asked to stand up fully and sit back down again once without using their upper limbs. The patients were asked to stand up fully and sit down again, landing on the seat firmly, five times as fast as possible, starting on the command “go.” | Using a stopwatch, the 5 repetitions were timed from the initial command to the completed fifth stand. This time was recorded as the participant’s score. If the patient was unable to perform the test in 30 sec, or not at all, this was captured, and the test score was recorded as 30 sec. | Chair, stopwatch  Cost: NR |
| **Physical outcome measure: 5-min walk test** | | | | | | |
| Physical measure: Distance walked | Jakobsson et al (2019)^35^ | The circuit was 30m long and octagonal. | Set up: NR Administer: 5 min | The patient was asked to walk as fast as possible (without running) for a 5-min period. | The distance covered was recorded in meters. | Walking circuit  Cost: NR |
| **Physical outcome measure: 6-min walk test** | | | | | | |
| Physical measure: Distance walked | Maldaner et al (2020)^13^ | 6WT app can be downloaded in the Apple iOS App Store and Google Android Play Store | Set up: NR Administer: 6 min | Participants were instructed on how to download and use the 6WT app. Instructions regarding usage  and the testing environment (a sufficiently long, straight, and level path with good GPS coverage) were given. A standardized information sheet was provided. | The 6WT app determines the maximum walking distance in 6 minutes. Calculated age and sex standardized scores. | Smartphone, 6WT app  Cost: 6WT app free |
|  | Maldaner et al (2021)^14^ | All trials were self-performed by the patient using the 6WT app | Set up: NR Administer: 6 min | Patients were instructed how to download and use the 6WT app during the first consultation. | NR | Smartphone, 6WT app  Cost: 6WT app free |
|  | Sosnova et al (2021)^38^ | Patient preference survey results indicated patients strongly agreed the 6WT App was convenient to perform. | Set up: NR Administer: 6 min | Patients were instructed how to download and use the 6WT App. Patient were then asked to perform the 6WT on a level area outside in their typical (home) environment or in the vicinity of the hospital. Detailed written instructions regarding usage and testing environment were handed out to the patient. They were instructed to walk as fast and as far as possible, aiming at a maximum 6-min walk distance. | 6WT App measures the distanced walks within 6 minutes based on global positioning system coordinates. | Smartphone, 6WT app  Cost: 6WT app free |
|  | Takenaka et al (2022)^36^ | Test was conducted on a straight 30-m indoor track, marked at 10m intervals for accurate measurement of distance. | Set up: NR Administer: 6 min | This test measures the distance that a patient can quickly walk on a flat, hard surface in 6 minutes. Patients were informed that they could rest if necessary. | A physiotherapist measured the distance walked. The test was ended when pain or numbness became too severe for the patient to walk. | Indoor track  Cost: NR |
|  | Takenaka et al (2023)^37^ | Test was conducted on a 30 m indoor path. | Set up: NR  Administer: 6 min | Patients did not use walking aids whenever possible. Use of a cane was permitted only if they were unable to walk. Use of a walker was not permitted. | Assessments were performed by a physical therapist. The test ended when pain or numbness became too severe for the patient to walk. If a patient could not walk even with a cane, the 6MWD was measured as 0 m. | Indoor track  Cost: NR |
|  | Zeitlberger et al (2022)^15^ | Test conducted using 6WT app with distance walked and time elapsed continuously displayed on screen. | Set up: NR Administer: 6 min | Patients were instructed to continue walking until 6 minutes elapsed, whenever possible. | 6WT app measures the maximum distance (in m) walked within six minutes using global positioning system coordinates. Completed measurements are saved on patient’s smartphone with a date and time stamp and may be transferred to a secure online database. | Smartphone, 6WT app  Cost: NR |
|  | Ziga et al (2023)^16^ | Test conducted using 6WT app, with distance walked and time elapses are continuously displayed on screen. | Set up: NR  Administer: 6 min | Patients were instructed how to download and use the 6WT-app. Instructions for how to perform the 6WT included to walk on a level and straight path in their typical (home) environment. | 6WT app measures the maximum distance walked within 6 minutes using global positioning system coordinates. | Smartphone, 6WT app.  Cost: 6WT app free |
| Physical measure: Distance to first symptoms | Zeitlberger et al (2022)^15^ | Test conducted using 6WT app with distance walked and time elapsed continuously displayed on screen. | Set up: NR Administer: 6 min | Patients were instructed to continue walking until 6 minutes elapsed, whenever possible. Patients were instructed to press a "flash" button on the app’s user interface in case of appearance and/or first-time  significant aggravation in leg or back pain during the test. This marks their walking distance to first symptoms. | Pressing the flash button on the 6WT app marks their distance to first symptoms. Measurements are saved on patient’s smartphone with a date and time stamp and may be transferred to a secure online database. | Smartphone, 6WT app  Cost: NR |
| Physical measure: Time to first symptoms | Zeitlberger et al (2022)^15^ | Test conducted using 6WT app with distance walked and time elapsed continuously displayed on screen. | Set up: NR Administer: 6 min | Patients were instructed to continue walking until 6 minutes elapsed, whenever possible. Patients were instructed to press a "flash" button on the app’s user interface in case of appearance and/or first-time  significant aggravation in leg or back pain during the test. This marks their time to first symptoms. | Pressing the flash button on the 6WT app marks their time to first symptoms. Measurements are saved on patient’s smartphone with a date and time stamp and may be transferred to a secure online database. | Smartphone, 6WT app  Cost: NR |
| **Physical outcome measure: 10-meter walk test** | | | | | | |
| Physical measure: Walking speed | Wada et al (2022)^17^ | Walking speed was evaluated using a 10-m walking test | Set up: NR  Administer: NR | NR | Walking speed was calculated from the time required to walk 10 m at a normal speed | Walking circuit  Cost: NR |
| **Physical outcome measure: 50-foot walk test** | | | | | | |
| Physical measure: Time to complete | Jakobsson et al (2019)^35^ | The circuit was 15m (approx. 50 ft) long and figure of 8 shaped. | Set up: NR  Administer: Mean (SD) scores pre-operative: 9.2 (2.8) sec | The patient was instructed to walk as fast as possible (without running) until they came back to the starting point. | The time needed to complete the test was rounded to the nearest 0.1 sec. | Walking circuit  Cost: NR |
| **Physical outcome measure: Modified Sorensen test** | | | | | | |
| Physical measure: Time to exhaustion | Dedering et al (2006)^18^ | The subjects lay prone on a bench with the lower extremities tightly secured with straps at hips, knees and ankles. Hips were flexed at 40°. | Set up: NR  Administer: Mean (SD) scores pre-operative: 175 (72) sec; post-operative: 187 (86) sec | During the test, the patients held the upper trunk horizontal and unsupported for as long as possible. They were unaware of the time that passed, and no verbal encouragement was given. | Endurance times measured were rounded down to the closest 5 s, but times less than 15 sec were rounded down to the closest second. | Bench and straps at hips, knees, ankles.  Cost: NR |
|  | Dedering (2012)^19^ | The patients lay prone with the lower extremities secured with belts to a bench at a pre-set hip angle of 40° and the arms were held crossed at the chest. | Set up: NR  Administer: Mean (SD) scores: 231 (86) sec | The patients were instructed to hold the unsupported trunk horizontal to exhaustion. | The test ended when the trunk changed vertical position more than 2 cm, as indicated by a light-activated sensor. | Bench, straps  Cost: NR |
| **Physical outcome measure: Self-paced walking test** | | | | | | |
| Physical measure: Distance walked | Tomkins-Lane et al (2020)^39^ | NR | Set up: NR Administer: ≤30 min | Subjects walked continuously at their own pace around the outer lane of a track until they felt they had to stop due to symptoms of lumbar spinal stenosis or until a time limit of 30 minutes had been reached. During the test, subjects were asked to alert the research staff when symptoms of LSS first began. | The distance of symptom onset and test completion were documented by research staff. | Track  Cost: NR |
| Physical measure: Time walked | Tomkins-Lane et al (2020)^39^ | NR | Set up: NR Administer: ≤30 min | Subjects walked continuously at their own pace around the outer lane of a track until they felt they had to stop due to symptoms of lumbar spinal stenosis or until a time limit of 30 minutes had been reached. During the test, subjects were asked to alert the research staff when symptoms of LSS first began. | The time of symptom onset and test completion were documented by research staff. | Track  Cost: NR |
| **Physical outcome measure: Timed up and go** | | | | | | |
| Physical measure: Time to complete | Corniola et al (2016)^22^ | NR | NR | NR | NR | NR |
|  | Gautschi et al (2016a)^23^ | Patients were allowed to wear their regular shoes and use a walking aid, if required. | Set up: NR  Administer: Mean (SD) scores: 11.3 (5.8) sec | Patients were asked to sit and lean back on an armchair with arms resting on the armrests. On command of the examiner, the patients had to get up and walk as fast as possible (without running) to a marked line on the floor at a 3-m distance from the chair. Once they reached the line, they made a 180° turn, returned to the chair, and sat down as quickly as possible. | Examiner recorded time (in sec). Time was stopped when the participant sat down again. Calculated z and t-scores. | Chair  Cost: NR |
|  | Gautschi et al (2016b)^40^ | Patients were allowed to wear regular shoes and use a walking aid, if required. | Set up: NR  Administer: Mean (SD) scores pre-operative: 10.3 (6.3) sec | Patients were asked to sit and lean back on an armchair with arms resting on the armrests. The patient was instructed to get up and walk as fast as possible (without running) to a marked line on the floor 3 m distance from the chair. Once the patient reached the line, they had to make a 180° turn, return to the chair, and sit down as quickly as possible. | The time was started when the patient got up and stopped when the patient sat down again. Measurements were performed using a regular stopwatch. Age and sex-adjusted t-scores were calculated. | Chair, stopwatch  Cost: NR |
|  | Jakobsson et al (2019)^35^ | Chair had seat 45 cm high, without armrests. | Set up: NR  Administer: Mean (SD) scores pre-operative: 7.8 (3.0) sec | The patient was asked to rise up  from a chair as fast as possible, walk (without running) 3m to a marked line on the floor, turn around, and walk back to the chair and sit down. | The time needed to  complete the test was rounded to the nearest 0.1 s. | Chair  Cost: NR |
|  | Maldaner et al (2021)^14^ | Test performance assessed using the TUG app, available for download from the Apple App Store and Google Play Store. | Set up: NR  Administer: Mean (SD) scores pre-operative: 10.44 (4.37) sec | The TUG test was performed using the TUG app. Patients were asked to sit on a chair with their arms resting on the armrests. On command, patients had to get up and walk as fast as possible (without running) to a marked line at a 3-m distance. Once the patient reached the marked line on the floor, he/she had to make a turn, return to the chair, and sit back down again. | The examiner recorded the time (sec) between sitting up and sitting down with the TUG app. | Chair, Smartphone, TUG app  Cost: TUG app free |
|  | Master et al (2020)^8^ | NR | Set up: NR  Administer: Mean (SD) scores: 15.5 (8.1) sec | This test has patients get up from a chair, walk 3 m away, walk back, then sit down. | NR | Chair  Cost: NR |
|  | Staartjes et al (2022)^21^ | Test performed using the TUG app. Patients were encouraged to wear their regular shoes and use a walking aid, if required. | Set up: NR  Administer: Ranges from CCI O: 10.62 (5.94) sec to ASA 3: 15.53 (18.10) sec | On the words “Three, Two, One—Go!” patients got up from a chair with an arm rest, walked as fast as possible (without running) to a line in 3-m distance. Then, they would turn around by 180 and return—again, as fast as possible—to the chair and sit down. | The time between getting up from the chair 10 and sitting back down was recorded using the TUG app. Raw TUG test times (in seconds) were transformed into OFI T-scores, based on age- and sex-adjusted > normative data, with T-scores 123 representing values that exceed the 99th percentile of the normal population and are .2 thus indicative of OFI. T-scores indicate how much a numerical value deviates from the population mean. As a transformation of Z-scores—the number of standard deviations that a value deviates from the population mean—T-scores are easier to compare with other tests, and were derived according to Gautschi et al. | Chair, Smartphone, TUG app  Cost: TUG app free |
|  | Stienen et al (2017)^24^ | NR | Set up: NR  Administer: Mean (SD) scores: Severe OFI: 221.3 (158.0) | On “Go,”’ patients got up and walked as fast as possible (no running) to a marked line on the floor at 3 m distance. At the line, they would turn around (180°) and return to the chair and sit down as quickly as possible. | The time between getting up and sitting down again was recorded in seconds using a stopwatch. Raw TUG test times were transformed into T-scores, and the presence and severity of OFI was determined using  age- and sex-adjusted norms. | NR |
|  | Stienen et al (2021)^20^ | Participants wore regular shoes. Walking aid (cane, walker) were allowed, if required. | Set up: NR  Administer: Mean (SD) scores: 10.8 (4.4) sec | Patients were asked to sit and lean back on an armchair. On command, patients had to get up and walk as fast as possible (without running) to a marked line on the floor at a 3-m distance from the chair. Once they reached the line, they made a 180° turn, returned to the chair, and sat down as quickly as possible. | The time required to perform the TUG test in sec was stopped when the participant sat down again. The free TUG app calculated the age- and sex-adjusted OFI z-scores from raw TUG test results. | Armchair, TUG app  Cost: free TUG app |
| **Physical outcome measure: Treadmill test** | | | | | | |
| Physical measure: Maximum walking distance | Herno et al (1999)^25^ | Treadmill test conducted at speed of 3600 m/h. | Set up: NR Administer: ≤15 min | NR | Maximum walking time of 15 min (i.e., 900 m). | Tunturi Treadmill  Cost: NR |
|  | Prasad et al (2016)^26^ | Evaluation with the treadmill at a speed of 2 km/h with a 0° slope was performed after 10 min of resting. | Set up: NR Administer: ≤20 min | The treadmill test was continued for the maximum amount time the patient could walk. | Distance was recorded at maximum time. The treadmill test was aborted if the patient had walked for 20 min or if cardiovascular symptoms appeared. | AEROFIT HK, 3.8 HP Treadmill  Cost: NR |
| Physical measure: Distance to first symptoms | Prasad et al (2016)^26^ | Evaluation with the treadmill at a speed of 2 km/h with a 0° slope was performed after 10 min of resting. | Set up: NR Administer: ≤20 min | The treadmill test was continued for the maximum amount time the patient could walk. | Distance at first symptom (cardiovascular or claudication symptom) was recorded. The treadmill test was aborted if the patient had walked for 20 min or if cardiovascular symptoms appeared. | AEROFIT HK, 3.8 HP Treadmill  Cost: NR |
| Physical measure: Maximum walking time | Deen et al (2000)^44^ | Treadmill test began at a walking speed of 1.2 mph and then at the patient’s preferred walking speed. A ramp incline of 0° was used for all examinations. All examinations were conducted during normal working hours, between 8am – 5pm. | Set up: NR Administer: ≤15 min | Patients were instructed to walk with an upright posture, to avoid using the front or side handrails, and tell the examiner when severe became severe, defined as the level of discomfort that would case patients to stop walking in usual life situations. | A time of zero was recorded when symptoms were present at the beginning of the test. The examination was stopped after 15 minutes or at the onset of severe symptoms and time was recorded. | Treadmill  Cost: NR |
|  | Prasad et al (2016)^26^ | Evaluation with the treadmill at a speed of 2 km/h with a 0° slope was performed after 10 min of resting. | Set up: NR Administer: ≤20 min | The treadmill test was continued for the maximum amount time the patient could walk. | Maximum amount time the patient could walk was recorded. The treadmill test was aborted if the patient had walked for 20 min or if cardiovascular symptoms appeared. | AEROFIT HK, 3.8 HP Treadmill  Cost: NR |
| Physical measure: Time to first symptoms | Deen et al (2000)^44^ | Treadmill test began at a walking speed of 1.2 mph and then at the patient’s preferred walking speed. A ramp incline of 0° was used for all examinations. All examinations were conducted during normal working hours, between 8am – 5pm. | Set up: NR Administer: ≤15 min | Patients were instructed to walk with an upright posture, to avoid using the front or side handrails, and tell the examiner when symptoms first appeared. | A time of zero was recorded when symptoms were present at the beginning of the test. The examination was stopped after 15 minutes if no symptoms were experienced. Time was recorded at first symptoms. | Treadmill  Cost: NR |
|  | Prasad et al (2016)^26^ | Evaluation with the treadmill at a speed of 2 km/h with a 0° slope was performed after 10 min of resting. | Set up: NR Administer: ≤20 min | The treadmill test was continued for the maximum amount time the patient could walk. | The first symptom time (time for first cardiovascular or claudication symptom to appear) was recorded. The treadmill test was aborted if the patient had walked for 20 min or if cardiovascular symptoms appeared. | AEROFIT HK, 3.8 HP Treadmill  Cost: NR |
| **Physical outcome measure: Trunk muscle endurance** | | | | | | |
| Physical measure: Repetitive arch-ups until exhaustion | Häkkinen et al (2005)^33^ | The subject was in the prone position with the arms along the sides, the inguinal region at the edge of the test bench, the upper trunk flexed downward at 45° and the feet fixed from the ankle region. | NR | The subject moved the upper trunk up to the horizontal position and back down. | Endurance strength measured by calculating the repetition maximum up to 100. | Bench  Cost: NR |
| Physical measure: Repetitive sit-ups until exhaustion | Häkkinen et al (2005)^33^ | The subject was in the supine position with the knees flexed at 90° and was held fixed by the tester in the ankle region. | NR | The subjects did sit-ups touching kneecaps with the thenar region. | Endurance strength measured by calculating the repetition maximum up to 100. | NA |
| **Activity in a natural environment physical outcome measures** | | | | | | |
| **Physical outcome measure: Step count** | | | | | | |
| Physical measure: Steps per day | Bienstock et al (2022)^29^ | Wrist-worn accelerometer and consumer-grade device. | Set up: NR Administer: Continuous daily assessment | Instructed to wear device on non-dominant wrist daily but not required to wear while sleeping. | NR | Fitbit Flex 2  Cost: NR |
|  | Kim et al (2019)^27^ | Small, lightweight and simple waist-worn device with 7-10 day battery life. | Set up: NR Administer: Continuous daily assessment | Instructed to wear on elastic band around waist at hip level on one side (left or right) and remove device when risk of contact with water. | Device has inbuilt algorithm to estimate number of steps taken and synchronizes wirelessly when placed within 6-m of active USB dongle | Fitbit Charge  Cost: NR |
|  | Mobbs et al (2016)^41^ | Small, lightweight commercially available waist-worn device with 6 month battery life. | NR | Device was clipped to the patient’s belt or waistband or worn in pant pockets. | Fitbit synced to patients’ smartphone or computer and estimated the number of steps taken | Fitbit Zip  Cost: NR |
|  | Stienen et al (2020)^28^ | NR | Set up: NR Administer: Wear as much as possible daily | Patients educated on use of Mi Band by a study physician, including how to properly wear / maintain the device and sync data. Patients encouraged to wear the device as much as possible. | Mi Band was interrogated by study staff for pre-operative data extraction. | Mi Band  Low cost |
| Physical measure: Steps detected at thigh | Gilmore et al (2020)^32^ | ActivPAL3 was fixed to thigh and does not have features that could irritate lumbar wound. Requires minimal input from the user.  Fitbit Flex and Jawbone UP Move are commercially available, low-cost monitors taped to the thigh, using off the shelf specifications. | Set up: NR  Administer: 2 min | Participants were instructed to walk around the hospital warm at a comfortable pace for 2 minutes, using their prescribed walking aid if required. | ActivPAL3 data were downloaded using software provided by PAL Technologies and number of steps detected was obtained. On completing the timed walk, the Fitbit and Jawbone were re-synced with the smartphone application and the step count over the 2-minute time period was recorded from the smartphone application. | ActivPAL3, Fitbit Flex, Jawbone UP Move  Low cost for Fitbit Flex and Jawbone UP Move |
| Physical measure: Steps detected at wrist | Gilmore et al (2020)^32^ | Fitbit Flex and Jawbone UP Move are commercially available, low cost monitors worn on the wrist and taped to the thigh, using off the shelf specifications. | Set up: NR  Administer: 2 min | Participants were instructed to walk around the hospital warm at a comfortable pace for 2 minutes, using their prescribed walking aid if required. | On completing the timed walk, the Fitbit and Jawbone were re-synced with the smartphone application and the step count over the 2-minute time period was recorded from the smartphone application. | Fitbit Flex, Jawbone UP Move  Low cost |
| **Physical outcome measure: Gait Posture Index** | | | | | | |
| Physical measure: Gait Posture Index | Ghent et al (2020)^42^ | NR | Set up: NR Administer: Continuous daily assessment for gait metrics. NR for posture. | Enrollment staff explained importance of continuous data collection pre- and post-operatively. | GPI scored 0 – 100 based on 4 metrics (3 gait, 1 posture), each weighted equally. Scoring criteria is established for all components and summed for total score. Accelerometers were paired to patients’ smart phones, and recorded data (step count, gait velocity, step length) were transferred to the clinician / researcher. Posture score based on measurement of the degree of trunk angulation in the coronal or sagittal planes at pre- and post-operative consultations. | Personal device (Apple Watch or Garmin device) or Mi Band 2  Cost: NR |
|  | Mobbs et al (2019)^43^ | NR | Set up: NR Administer: Continuous daily assessment for step count. All others NR | Daily step count: NR. Gait velocity and step length: patients instructed to walk unobstructed on 120 m course, or 30 m course if unable to complete the full distance, or at risk of falls. Posture: A picture was taken of the patient during walking on the flat. | GPI scored 0 – 100 based on 4 metrics (3 gait, 1 posture), each weighted equally. Scoring criteria is established for all components and summed for total score. For step count, accelerometer assessed and synced to patients’ smartphone which was recorded by researcher on presentation to clinic. For gait velocity and step length, time of walking test completion and step length were recorded. For posture, the picture was scored by observation. | Personal device or Mi Band 2 |
| **Physical outcome measure: Distance per day** | | | | | | |
| Physical measure: Distance per day (km / day using Fibit Zip^41^ and miles / day using Apple iOS Apple Health activity data from personal device^30^) | Mobbs et al (2016)^41^ | Small, lightweight commercially available waist-worn device with 6 month battery life | NR | Device was clipped to the patient’s belt or waistband or worn in pant pockets. | Fitbit synced to patients’ smartphone or computer and estimated the distance traveled per day | Fitbit Zip  Cost: NR |
|  | Voglis et al (2022)^30^ | Participant’s Apple iOS Smartphones were used to quantify daily walking distance using Apple Health activity data. The QS Access app enabled exporting Apple Health data and sent it via secure mail to researchers. | Set up: NR  Continuous daily assessment | Participants were instructed to download the “QS Access” app via the Apple iOS App Store. No instructions provided for walking distance. | Raw smartphone hourly mile counts of all patients were extracted and used to derive summed daily distances. Each patient’s daily distances were normalized by his/her mean daily distance over the entire observation period. | Apple iOS Smartphone, QS Access app, Apple Health activity data  Cost: NR |
| **Physical outcome measure: Gait cycles** | | | | | | |
| Physical measure: Gait cycles per day | Schulte et al (2010)^31^ | Robust, small, lightweight, unobtrusive ankle-worn monitor | Set up: NR Continuous daily assessment, except at night | Patients were asked to wear the monitor for 7 consecutive days from getting up in the morning until bed rest in the evening and to document individual wearing times in a log list. | Data downloaded to a computer via an infrared interface and the software identified and provided the number of gait cycles | StepWatch 3 Activity Monitor  Cost: NR |
| Physical measure: Gait cycles per hour | Schulte et al (2010)^31^ | Robust, small, lightweight, unobtrusive ankle-worn monitor | Set up: NR Continuous daily assessment, except at night | Patients were asked to wear the monitor for 7 consecutive days from getting up in the morning until bed rest in the evening and to document individual wearing times in a log list. | Data downloaded to a computer via an infrared interface and the software identified and provided the number of gait cycles | StepWatch 3 Activity Monitor  Cost: NR |
| Physical measure: Gait intensities per day | Schulte et al (2010)^31^ | Robust, small, lightweight, unobtrusive ankle-worn monitor | Set up: NR Continuous daily assessment, except at night | Patients were asked to wear the monitor for 7 consecutive days from getting up in the morning until bed rest in the evening and to document individual wearing times in a log list. | Data downloaded to a computer via an infrared interface and the software identified and provided the number of gait cycles. Gait intensities were measured as gait cycles per minute | StepWatch 3 Activity Monitor  Cost: NR |

^a^ Ease of administration refers to procedures and methods to administer physical outcome measure.^45^

^b^ Time to complete refers to time to set up and administer physical outcome measure.

^c^ Ease of standardization refers to specific instructions given to participants.^45^

^d^ Ease of score calculation refers to methods for obtaining result of physical outcome measure.

^e^ Required equipment and cost refers to equipment reported by study authors for collecting physical outcome measures.

No study assessed: Patient comprehensibility, Clinician comprehensibility, Patient’s required mental and physical ability level, Copywrite, or Regulatory agency’s requirement for approval. Per systematic review eligibility criteria: All physical outcome measures are practical within clinical, hospital or community Physical Therapy settings.

Km, Kilometers; hr, Hour; NA, Not applicable; NR, Not reported; m, Meters; min, Minutes sec, Seconds

**References**

1. Prinsen CAC, Mokkink LB, Bouter LM, et al. COSMIN guideline for systematic reviews of patient-reported outcome measures. *Quality of Life Research*. 2018;27:1147-1157. doi:10.1007/S11136-018-1798-3

2. Mannion AF, Dvorak J, Müntener M, Grob D. A prospective study of the interrelationship between subjective and objective measures of disability before and 2 months after lumbar decompression surgery for disc herniation. *European Spine Journal*. 2005;14(5):454-465. doi:10.1007/s00586-004-0787-0

3. Pitino T. *The Impairment and Disability Relationship in Patients Who Have Undergone Lumbar Discectomy*. Northeastern University; 2000.

4. Kwon JW, Lee BH, Sung S, et al. Hand grip strength as a surrogate marker for postoperative changes in spinopelvic alignment in patients with lumbar spinal stenosis. *Scientific Reports 2020 10:1*. 2020;10(1):1-6. doi:10.1038/s41598-020-70357-8

5. Kwon JW, Lee BH, Lee S bin, et al. Hand grip strength can predict clinical outcomes and risk of falls after decompression and instrumented posterolateral fusion for lumbar spinal stenosis. *Spine Journal*. 2020;20(12):1960-1967. doi:10.1016/j.spinee.2020.06.022

6. Inoue H, Watanabe H, Okami H, Shiraishi Y, Kimura A, Takeshita K. Handgrip strength correlates with walking in lumbar spinal stenosis. *European Spine Journal*. 2020;29(9):2198-2204. doi:10.1007/S00586-020-06525-1/TABLES/2

7. Fujita N, Sakurai A, Miyamoto A, et al. Stride length of elderly patients with lumbar spinal stenosis: Multi-center study using the Two-Step test. *Journal of Orthopaedic Science*. 2019;24(5):787-792. doi:10.1016/J.JOS.2019.01.006

8. Master H, Pennings JS, Coronado RA, et al. Physical Performance Tests Provide Distinct Information in Both Predicting and Assessing Patient-Reported Outcomes Following Lumbar Spine Surgery. *Spine (Phila Pa 1976)*. 2020;45(23):E1556-E1563. doi:10.1097/BRS.0000000000003665

9. Staartjes VE, Beusekamp F, Schröder ML. Can objective functional impairment in lumbar degenerative disease be reliably assessed at home using the five-repetition sit-to-stand test? A prospective study. *European Spine Journal*. 2019;28(4):665-673. doi:10.1007/s00586-019-05897-3

10. Staartjes VE, Klukowska AM, Schröder ML. Association of maximum back and leg pain severity with objective functional impairment as assessed by five-repetition sit-to-stand testing: analysis of two prospective studies. *Neurosurg Rev*. 2020;43(5):1331-1338. doi:10.1007/S10143-019-01168-3/TABLES/4

11. Klukowska AM, Schröder ML, Stienen MN, Staartjes VE. Objective functional impairment in lumbar degenerative disease: concurrent validity of the baseline severity stratification for the five-repetition sit-to-stand test. *J Neurosurg Spine*. 2020;33(1):4-11. doi:10.3171/2019.12.SPINE191124

12. Staartjes VE, Schröder ML. The five-repetition sit-to-stand test: Evaluation of a simple and objective tool for the assessment of degenerative pathologies of the lumbar spine. *J Neurosurg Spine*. 2018;29(4):380-387. doi:10.3171/2018.2.SPINE171416

13. Maldaner N, Sosnova M, Zeitlberger AM, et al. Evaluation of the 6-minute walking test as a smartphone app-based self-measurement of objective functional impairment in patients with lumbar degenerative disc disease. *J Neurosurg Spine*. 2020;33(6):779-788. doi:10.3171/2020.5.SPINE20547

14. Maldaner N, Sosnova M, Zeitlberger AM, et al. Responsiveness of the self-measured 6-minute walking test and the Timed up and Go test in patients with degenerative lumbar disorders. *J Neurosurg Spine*. 2021;35(1):52-59. doi:10.3171/2020.11.SPINE201621

15. Zeitlberger AM, Sosnova M, Ziga M, et al. Distance to first symptoms measured by the 6-min walking test differentiates between treatment success and failure in patients with degenerative lumbar disorders. *European Spine Journal*. 2022;31(3):596-603. doi:10.1007/S00586-021-07103-9/FIGURES/2

16. Ziga M, Sosnova M, Zeitlberger AM, et al. Objective outcome measures may demonstrate continued change in functional recovery in patients with ceiling effects of subjective patient-reported outcome measures after surgery for lumbar degenerative disorders. *The Spine Journal*. Published online 2023. doi:10.1016/j.spinee.2023.05.002

17. Wada T, Tanishima S, Kitsuda Y, et al. Walking speed is associated with postoperative pain catastrophizing in patients with lumbar spinal stenosis: a prospective observational study. *BMC Musculoskelet Disord*. 2022;23(1). doi:10.1186/s12891-022-06086-y

18. Dedering Å, Harms-Ringdahl K, Nèmeth G. Back extensor muscle fatigue in patients with lumbar disc herniation: Pre-operative and post-operative analysis of electromyography, endurance time and subjective factors. *European Spine Journal*. 2006;15(5):559-569. doi:10.1007/s00586-005-0934-2

19. Dedering Å. Lumbar muscle fatigue and subjective health measurements in patients with lumbar disc herniation 2 years after surgery. *European Spine Journal*. 2012;21(4):646-654. doi:10.1007/S00586-011-2109-7/FIGURES/2

20. Stienen MN, Maldaner N, Sosnova M, et al. External Validation of the Timed up and Go Test as Measure of Objective Functional Impairment in Patients with Lumbar Degenerative Disc Disease. *Neurosurgery*. 2021;88(2):E142-E149. doi:10.1093/NEUROS/NYAA441

21. Staartjes VE, Joswig H, Corniola M V., Schaller K, Gautschi OP, Stienen MN. Association of Medical Comorbidities With Objective Functional Impairment in Lumbar Degenerative Disc Disease. *Global Spine J*. 2022;12(6). doi:10.1177/2192568220979120

22. Corniola M v., Stienen MN, Joswig H, et al. Correlation of pain, functional impairment, and health-related quality of life with radiological grading scales of lumbar degenerative disc disease. *Acta Neurochir (Wien)*. 2016;158(3):499-505. doi:10.1007/S00701-015-2700-5/TABLES/8

23. Gautschi OP, Smoll NR, Corniola M v., et al. Validity and reliability of a measurement of objective functional impairment in lumbar degenerative disc disease: The Timed Up and Go (TUG) test. *Neurosurgery*. 2016;79(2):270-278. doi:10.1227/NEU.0000000000001195

24. Stienen MN, Smoll NR, Joswig H, et al. Validation of the baseline severity stratification of objective functional impairment in lumbar degenerative disc disease. *J Neurosurg Spine*. 2017;26(5). doi:10.3171/2016.11.SPINE16683

25. Herno A, Partanen K, Talaslahti T, et al. Long-term clinical and magnetic resonance imaging follow-up assessment of patients with lumbar spinal stenosis after laminectomy. *Spine (Phila Pa 1976)*. 1999;24(15). doi:10.1097/00007632-199908010-00006

26. Prasad BCM, Chandra VVR, Devi BV, Chivukula SS, Pundarikakshaiah K. Clinical, radiological, and functional evaluation of surgical treatment in degenerative lumbar canal stenosis. *Neurol India*. 2016;64(4):683. doi:10.4103/0028-3886.185378

27. Kim DH, Nam KH, Choi BK, Han IH, Jeon TJ, Park SY. The Usefulness of a Wearable Device in Daily Physical Activity Monitoring for the Hospitalized Patients Undergo-ing Lumbar Surgery. *J Korean Neurosurg Soc*. 2019;62(5):561-566. doi:10.3340/jkns.2018.0131

28. Stienen MN, Rezaii PG, Ho AL, et al. Objective activity tracking in spine surgery: a prospective feasibility study with a low-cost consumer grade wearable accelerometer. *Sci Rep*. 2020;10(1):1-11. doi:10.1038/s41598-020-61893-4

29. Bienstock DM, Shankar D, Kim J, et al. Accelerometry Data Delineate Phases of Recovery and Supplement Patient-Reported Outcome Measures Following Lumbar Laminectomy. *World Neurosurg*. 2022;160:e608-e615. doi:10.1016/J.WNEU.2022.01.097

30. Voglis S, Ziga M, Zeitlberger AM, et al. Smartphone-based real-life activity data for physical performance outcome in comparison to conventional subjective and objective outcome measures after degenerative lumbar spine surgery. *Brain and Spine*. 2022;2. doi:10.1016/j.bas.2022.100881

31. Schulte TL, Schubert T, Winter C, et al. Step activity monitoring in lumbar stenosis patients undergoing decompressive surgery. *European Spine Journal*. 2010;19(11):1855-1864. doi:10.1007/S00586-010-1324-Y/TABLES/2

32. Gilmore SJ, Davidson M, Hahne AJ, McClelland JA. The validity of using activity monitors to detect step count after lumbar fusion surgery. *Disabil Rehabil*. 2020;42(6):863-868. doi:10.1080/09638288.2018.1509140

33. Häkkinen A, Kautiainen H, Sintonen H, Ylinen J. Health related quality of life after lumbar disc surgery: A prospective study of 145 patients. *Disabil Rehabil*. 2005;27(3):94-100. doi:10.1080/09638280400007430

34. Loske S, Nüesch C, Byrnes KS, et al. Decompression surgery improves gait quality in patients with symptomatic lumbar spinal stenosis. *Spine Journal*. 2018;18(12):2195-2204. doi:10.1016/j.spinee.2018.04.016

35. Jakobsson M, Brisby H, Gutke A, Lundberg M, Smeets R. One-minute stair climbing, 50-foot walk, and timed up-and-go were responsive measures for patients with chronic low back pain undergoing lumbar fusion surgery. *BMC Musculoskelet Disord*. 2019;20:137. doi:10.1186/s12891-019-2512-5

36. Takenaka H, Kamiya M, Sugiura H, et al. Responsiveness and Minimal Clinically Important Difference of the 6-minute Walk Distance in Patients Undergoing Lumbar Spinal Canal Stenosis Surgery. *Clin Spine Surg*. 2022;35(3):E345-E350. doi:10.1097/BSD.0000000000001196

37. Takenaka H, Kamiya M, Sugiura H, Nishihama K, Suzuki J, Hanamura S. Minimal Clinically Important Difference of the 6-Minute Walk Distance in Patients Undergoing Lumbar Spinal Canal Stenosis Surgery: 12 Months Follow-Up. *Spine (Phila Pa 1976)*. 2023;48(8). doi:10.1097/BRS.0000000000004566

38. Sosnova M, Zeitlberger AM, Ziga M, et al. Patients undergoing surgery for lumbar degenerative spinal disorders favor smartphone-based objective self-assessment over paper-based patient-reported outcome measures. *The Spine Journal*. 2021;21(4):610-617. doi:10.1016/J.SPINEE.2020.11.013

39. Tomkins-Lane C, Zheng PZ, Sun R, Smuck M. Variability among methods and timing of pain assessment tools for tracking improvement of lumbar stenosis patients after surgery. *Spine Journal*. 2020;20(11):1826-1831. doi:10.1016/j.spinee.2020.06.003

40. Gautschi OP, Joswig H, Corniola M v., et al. Pre- and postoperative correlation of patient-reported outcome measures with standardized Timed Up and Go (TUG) test results in lumbar degenerative disc disease. *Acta Neurochir (Wien)*. 2016;158(10):1875-1881. doi:10.1007/S00701-016-2899-9/FIGURES/2

41. Mobbs RJ, Phan K, Maharaj M, Rao PJ. Physical Activity Measured with Accelerometer and Self-Rated Disability in Lumbar Spine Surgery: A Prospective Study. *Global Spine J*. 2016;6(5):459-464. doi:10.1055/S-0035-1565259

42. Ghent F, Mobbs RJ, Mobbs RR, Sy L, Betteridge C, Choy WJ. Assessment and Post-Intervention Recovery After Surgery for Lumbar Disk Herniation Based on Objective Gait Metrics from Wearable Devices Using the Gait Posture Index. *World Neurosurg*. 2020;142:e111-e116. doi:10.1016/J.WNEU.2020.06.104

43. Mobbs RJ, Mobbs RR, Choy WJ. Proposed objective scoring algorithm for assessment and intervention recovery following surgery for lumbar spinal stenosis based on relevant gait metrics from wearable devices: the Gait Posture index (GPi). *Journal of Spine Surgery*. 2019;5(3):300-309. doi:10.21037/JSS.2019.09.06

44. Deen HGJ, Zimmerman RS, Lyons MK, McPhee MC, Verheijde JL, Lemens SM. Test-retest reproducibility of the exercise treadmill examination in lumbar spinal stenosis. *Mayo Clin Proc*. 2000;75:1002-1007.

45. Hoglund LT, Scalzitti DA, Bolgla LA, Jayaseelan DJ, Wainwright SF. Patient-Reported Outcome Measures for Adults and Adolescents with Patellofemoral Pain: A Systematic Review of Content Validity and Feasibility Using the COSMIN Methodology. *J Orthop Sports Phys Ther*. 2023;53(1):23-39. doi:10.2519/jospt.2022.11317
