# Supplementary material for: Physical functioning in the lumbar spinal surgery population: A systematic review and narrative synthesis of outcome measures and measurement properties of the physical measures
Source: PLoS One. 2024 Aug 29;19(8):e0307004. doi: 10.1371/journal.pone.0307004 (PMC11361614; doi:10.1371/journal.pone.0307004)
Supplement: Attachment — Submitted filename: Kowalski et al Point-by-point response to reviewers.docx [file pone.0307004.s008.docx]

June 3, 2024

**Author’s point-by-point response to reviews**

**Title:** Physical functioning in the lumbar spinal surgery population: a systematic review and narrative synthesis of outcome measures and measurement properties of the physical measures

**Authors:**

Katie Kowalski ([kkowals7@uwo.ca](mailto:kkowals7@uwo.ca))

Jai Mistry ([jmistr22@uwo.ca](mailto:jmistr22@uwo.ca))

Anthony Beilin (abeilin3@uwo.ca)

Maren Goodman ([maren.goodman@uwo.ca](mailto:maren.goodman@uwo.ca))

Michael Lukacs ([mlukacs2@uwo.ca](mailto:mlukacs2@uwo.ca))

Alison Rushton (arushto3@uwo.ca)

| **Reviewer 1 comments** | **Author’s Response** |
| --- | --- |
| The review is relevant and professionally written. To avoid confusing study design with current study design, it is advised to change the sub-heading "study design" to "design of included study". Page 7, line 127. | Thank you for this feedback.  We have clarified this eligibility criteria sub-heading to reflect study design of included studies. |

| **Reviewer 2 comments** | **Author’s Response** |
| --- | --- |
| figure 1: is not readable, please replace it with a high-resolution figure. | A new Fig 1 file has been uploaded with the following file size properties: 5760x7811 pixels, 768 dpi |
| Page 4 / Line 54/55: First line therapy for LBP is conservative treatment, Please state why you concentrated your review on surgigal intervention. Does outcome measures differ between conservativ and surgical interventions? | Thank you for this suggestion. We have added a statement starting on line 55 highlighting how population-specific outcome measures are recommended for use when measuring treatment outcomes for specific clinical populations and when focusing on the individual, an important component of providing patient-centered care. |
| objectives: - the first objective includes patient reported and physical outcome measures. The sencond objective only physical outcomes. Why did you include PROMs in the first step but not in the second step? | Thank you for pointing this out. There are 2 reasons for this decision. 1: Systematic reviews of PROM measurement properties exist, and we have added this to the end of the intro (Line 93), yet there is still no contemporary comprehensive resource outlining all PROMs of physical functioning. 2: This is also a pragmatic decision, as the systematic review would have been very large had evaluations of PROMs and physical measures been included in objective 2. |
| I- In addition to the measurement properities, you also assessed interpretability and feasibility. Pleas add this part to your objectives, | We have added to objective 2 that interpretability and feasibility were described (Line 100 and 27-28 in abstract). We have also added to the introduction that COSMIN recommends considering interpretability and feasibility when selecting outcome measures, as further support to describing interpretability and feasibility as one step in the process of conducting a systematic review of measurement properties (Line 89). |
| Page 9 / 184/185: Why are studies using a criterion approach considered responsivenss. This is not in accordance with COSMIN. Pleas refer to the definition of COSMIN (for validity and responsiveness) | We used the terminology of criterion approach for responsiveness as this is how it is described in the COSMIN risk of bias checklist for responsiveness (Box 10a), for example in studies where area under the receiver operating characteristic curve was used to evaluate responsiveness. This terminology is also useful to differentiate this approach from hypothesis testing construct approaches for responsiveness. We have added the reference for the COSMIN risk of bias checklist to support use of this terminology. |
| Page 10 / Line 190: what is meant by "study authors" (authors of this review or of the included studies)? Why would formulating hypothesis elevate ROB? Pleas explaain this connection. | We have clarified ‘study authors’ as ‘authors of included studies’ and that lack of a priori hypotheses introduces threats to internal validity of included studies and therefore this systematic review would have provided an inaccurate representation of the quality of the literature (Lines 196-199). |
| Page 14 / Line261-264: What is the difference between physical measures and physical outcone measures? Pleas define these two terms. | We have added definitions for physical measures and physical outcome measures to supplement the examples already provided (Lines 268-270). |
| Table 1: please indicate what sort of reliability was evaluated (intra, inter, test-retest)  Table 4: dito table 1 | In Tables 1 and 4, we have further described the type of reliability being evaluated. |
